# Most People Keep Their Word Rather Than Their Money

**DOI:** 10.1162/opmi_a_00027

**Published:** 2019-07-01

**Authors:** Jan K. Woike, Patricia Kanngiesser

**Affiliations:** Center for Adaptive Rationality (ARC), Max Planck Institute for Human Development, Berlin, Germany; Faculty of Education and Psychology, Freie Universität Berlin, Berlin, Germany

**Keywords:** cooperation, social norms, promises, agreements, contracts

## Abstract

Promises are crucial for human cooperation because they allow people to enter into voluntary commitments about future behavior. Here we present a novel, fully incentivized paradigm to measure voluntary and costly promise-keeping in the absence of external sanctions. We found across three studies (*N* = 4,453) that the majority of participants (61%–98%) kept their promises to pay back a specified amount of a monetary endowment, and most justified their decisions by referring to obligations and norms. Varying promise elicitation methods (Study 1a) and manipulating stake sizes (Study 2a) had negligible effects. Simultaneously, when others estimated promise-keeping rates (using two different estimation methods), they systematically underestimated promise-keeping by up to 40% (Studies 1b and 2b). Additional robustness checks to reduce potential reputational concerns and possible demand effects revealed that the majority of people still kept their word (Study 3). Promises have a strong normative power and binding effect on behavior. Nevertheless, people appear to pessimistically underestimate the power of others’ promises. This behavior–estimation gap may prevent efficient coordination and cooperation.

## INTRODUCTION

Large-scale human cooperation depends on commitments, made in the present and to be honored in the future. Promises create such commitments: By simply saying “I promise to do *X*” (or an equivalent phrase), under the appropriate conditions one is obligated to do *X* (Austin, 1975; Searle, 1969). Some have argued that promises without threats of sanctions are just empty words with no binding power (Hobbes, 2016). Yet research has shown that promises and verbal agreements increase cooperation rates in social dilemma games (Bicchieri, 2002; Charness & Dufwenberg, 2006; Ellingsen & Johannesson, 2004; Orbell, van de Kragt, & Dawes, 1988; Ostrom, Walker, & Gardner, 1992; Sally, 1995; van den Assem, van Dolder, & Thaler, 2012) and are more effective than punishment or threats (Ellingsen & Johannesson, 2004; Ostrom et al., 1992).

While the effect of communication on cooperation is well established, it is unclear whether promises in social dilemmas are effective because of the act of promising or because people use promises to signal their intentions to other players when given the opportunity to communicate (Ismayilov & Potters, 2016; van den Assem et al., 2012). That is, in social dilemmas, promises may be used strategically to gain a partner’s trust and cooperation and to reduce uncertainty about future behavior. There have been some recent attempts to measure promise-keeping with paradigms that did not involve strategic interactions: For example, Mischkowski, Stone, and Stremitzer (2016) used vignettes to ask participants about the likelihood of buying a product from a seller after a promise and Conrads and Reggiani (2017) elicited nonincentivized promises to take part in a survey. However, neither promise-keeping nor promise-breaking in these studies resulted in a cost for participants; it remains unclear how people will react when keeping a promise comes at a monetary cost. Here, we propose a novel, fully incentivized minimal promising paradigm, in which promises are clearly defined and consequential and can be studied outside of strategic interactions.

In a series of experiments (Studies 1a, 2a, and 3), workers in an online labor market could choose between (i) a small sum of money (e.g., $0.50; henceforth, all amounts in USD) or (ii) a larger sum of money (e.g., $2) under the condition that they make a commitment to pay back a fixed sum (e.g., $1) at a later point. Choosing the commitment option (with or without paying back the fixed amount) thus always yielded a higher payoff than the no-commitment option. These incentives were set so that payoffs for cooperators dominated payoffs for noncommitment, giving even potentially uncooperative and untrustworthy participants a reason to make a promise (and an incentive to break it). We ensured that participants could decide and were incentivized to enter the commitment but were not forced to do so (see Kataria & Winter, 2013; Shu, Mazar, Gino, Ariely, & Bazerman, 2012, for forced oaths) for two reasons: (i) because promising is by definition a voluntary act (Searle, 1969), and (ii) to respect individual autonomy and decision making (Hertwig & Grüne-Yanoff, 2017). This paradigm has several unique features: In contrast to the free-form communication used in previous studies, our promises were clearly marked as speech acts of commitment (e.g., they contained the word “promise”) and it was unambiguous for both promisor and promisee that a promise had been made. Furthermore, our paradigm did not involve any uncertainty about the consequence of the promise and its payoffs.

To get insight into people’s motives for keeping their promises (Bhattacharya & Sengupta, 2016; Charness & Dufwenberg, 2006, 2010; Di Bartolomeo, Dufwenberg, Papa, & Passarelli, 2018; Ederer & Stremitzer, 2017; Ellingsen & Johannesson, 2004; Mischkowski et al., 2016; Vanberg, 2008) and their experience of the process, we asked different groups of participants in Studies 1a and 2a to justify their decisions and assessed their positive and negative affect using the Positive and Negative Affect Schedule (PANAS) scale (Crawford & Henry, 2004). Moreover, to find out whether promise-keeping behavior would be accurately predicted (Belot, Bhaskar, & van de Ven, 2012; Chen & Houser, 2017), we asked new groups of participants to retrodict promise-keeping rates in our paradigm using two different incentivized methods (Studies 1b and 2b).

## STUDY 1A

### Methods

#### Participants

All participants were located in the United States and recruited through Amazon’s Mechanical Turk (MTurk). Qualifications and exclusion criteria (double IP addresses, failed attention checks, etc.) for all studies are described in the Supplemental Material (Woike & Kanngiesser, 2019b). Double participation was prevented by a combination of using the Unique Turker script, checking against exclusion lists at the beginning of the survey, and carrying out manual checks of MTurk IDs and IP addresses. After these steps, there were *n*_1*a*_ = 568 participants in Study 1a (*M*_*age*_ = 36.1, 55.6% female, 44.4% male). All participants gave their informed consent and the study was approved by the Institutional Review Board (IRB) at the Max Planck Institute for Human Development.

#### Procedure

Workers were offered a choice between (a) $0.05 and (b) $0.15 under the condition that they commit to paying back $0.05 (henceforth **15–5**). Since there have been few systematic studies on the effect of different commitment formats on promise-keeping (Charness & Dufwenberg, 2010; Turmunkh, van den Assem, & van Dolder, 2019), we decided to address this question by implementing the following commitment variants in a between-subjects design: (1) promise (write): clicking on a radio button with the promise spelled out and typing “I promise” into a text box next to it (*n* = 147), (2) promise (click): only clicking on a radio button to promise (*n* = 146), and (3) *ask*: being asked to pay back a suggested amount of money with no mention of the word “promise” (*n* = 145). We also included (4) a noncommitment *control* condition, in which participants had the option to select (a) $0.05 or (b) $0.15 with the choice to pay back $0.05 or a different amount (*n* = 130). Workers received the final amount (i.e., the chosen amount minus any money they decided to return) as part of their bonus payment a few days after the study had finished.

To illustrate the wording we used when asking participants to choose between the lower and the higher payoff, we take the promise (click) condition as an example (for the wording in the other conditions, see the Supplemental Materials, Woike & Kanngiesser, 2019b): “You have two options that have consequences for your bonus payment: 1. You can receive 5 cents without any further consequences. 2. You can receive 15 cents. In this case we would ask you to promise that you will give back 5 cents at the end of the survey. (We would ask you to pay back this money at the end).” Participants were then offered two options and had to choose one using a click button: “I take 5 cents” or “I take 15 cents and I promise to pay back 5 cents when asked to at the end of the survey.”

Participants then completed a series of unrelated tasks. After the tasks, those who had accepted $0.15 were reminded of their choice (in two conditions, also of their promise) and given a chance to pay back $0.00–$0.10. We decided to remind participants of their choice or promise to ensure that a decision against paying back money was not due to memory errors. Moreover, participants were informed that there would be no negative consequences no matter which amount they chose. In the promise conditions, we used the following wording (the wording in the other conditions was similar; for details see the Supplemental Materials, Woike & Kanngiesser, 2019b): “We gave you a choice at the beginning of the survey between taking 5 cents or 15 cents. You decided to take 15 cents and promised to pay back 5 cents. We now ask you whether you want to pay back 5 cents. We will not force you to pay any money back. How much money (in cents) do you pay back?” Participants could then enter the payback amount in a text box.

Subsequently, participants completed the PANAS scale (Crawford & Henry, 2004) with items in randomized order. Finally, they were asked to provide reasons for their decision in an open-ended format. Open-ended answers were categorized by two independent coders based on a coding scheme developed by the authors (for details, see the Supplemental Materials, Woike & Kanngiesser, 2019b). As the dependent variables included proportions, we aimed for sample sizes well beyond 100 participants for each condition in order to obtain measures with reasonable precision.

#### Survey and Analysis Software

The survey was implemented in Qualtrics. Allocation to conditions used the Qualtrics randomizer with samples sizes kept constant. Statistical analyses were conducted with IBM SPSS 24 and the Exploratory Software for Confidence Intervals (ESCI), downloaded from Cumming (2014; see Cumming, 2012).

### Results

#### Acceptance Decision

**Figure 1.**
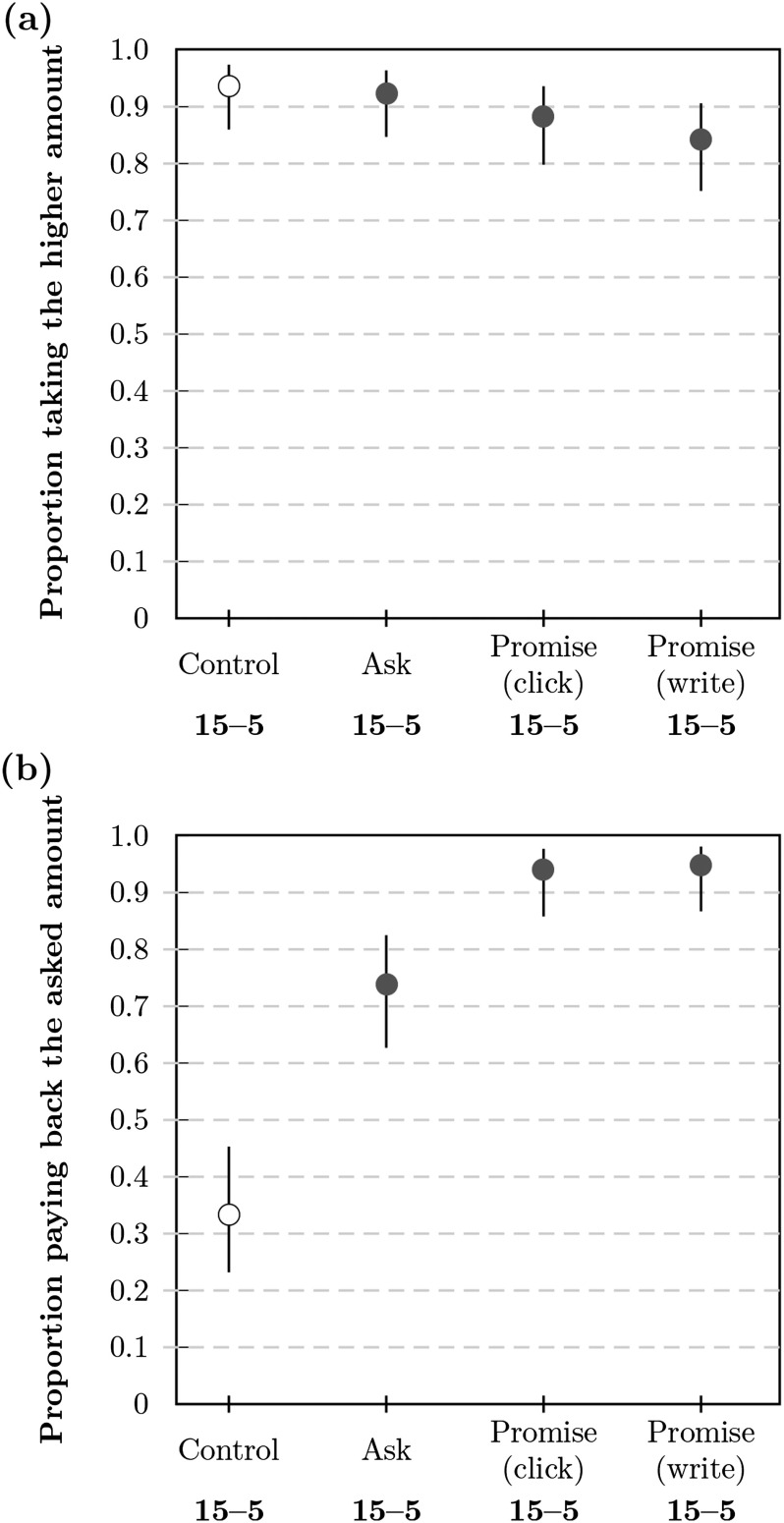
**Proportion of participants in Study 1a (a) accepting the higher amount of $0.15 in the beginning and (b) repaying ≥$0.05 at the end.** Proportions are shown as markers for each condition with bars corresponding to the 99% CI (confidence interval). The payback proportion is relative to the number of participants accepting the higher amount.

Across conditions the majority of participants accepted the higher amount (see Figure 1a). Specifically, the acceptance rate was *a*_*pw*_ = 0.84 (99% CI [confidence interval] = [0.75, 0.91]) in the promise (write) condition, *a*_*pc*_ = 0.88 ( 99% CI = [0.80, 0.94]) in the promise (click) condition, and *a*_*a*_ = 0.92 (99% CI [0.85, 0.96]) in the ask condition. The highest acceptance rate was observed in the control condition with *a*_*c*_ = 0.94 (99% CI = [0.86, 0.97]).

#### Payback Decision

Next, we analyzed payback rates among those who had accepted the higher payment of $0.15, with payback defined as returning at least the promised or suggested amount of $0.05 (see Figure 1b). The vast majority of participants repaid, as promised, in the promise (write) condition (*b*_*pw*_ = 0.95, 99% CI = [0.87, 0.98], *n* = 116) and the promise (click) condition (*b*_*pc*_ = 0.94, 99% CI = [0.86, 0.98], *n* = 118). Payback rates were lower in the ask condition (*b*_*a*_ = 0.74, 99% CI = [0.63, 0.83], *n* = 126) and very low in the control condition (*b*_*c*_ = 0.33, 99% CI = [0.23, 0.45], *n* = 114). Details of the distribution of payback amounts can be found in the Supplemental Materials (Woike & Kanngiesser, 2019b; see Figure S33).

#### Affect

We analyzed the positive and negative PANAS subscales with two separate ANOVAs, entering the payback above threshold (yes/no) as one factor and condition as a second factor. For positive affect, there were no significant main effects of condition [*F*(3, 465) = 2.18, *p* = .09, partial η^2^ = .01] and payback [*F*(1, 465) = 0.07, *p* = .80, partial η^2^ = .00], or their two-way interaction [*F*(3, 465) = 1.50, *p* = .22, partial η^2^ = .01]. For negative affect, we found a significant main effect of payback [*F*(1, 465) = 19.60, *p* < .001, partial η^2^ = .04], but no significant main effect of condition [*F*(3, 465) = 0.24, *p* = .87, partial η^2^ = .00] nor a significant two-way-interaction [*F*(3, 465) = 0.88, *p* = .45, partial η^2^ = .01]. Participants who repaid below threshold experienced significantly more negative affect (*M* = 3.89, 99% CI = [2.40, 5.38]) than those who repaid above threshold (*M* = 1.15, 99% CI = [0.56, 1.74]).

#### Reasons

Justifications were scored separately for participants who (1) took the lower payment (i.e., rejected $0.15), (2) paid back less than $0.05, and (3) paid back $0.05 or more (see Table 1 and the Supplemental Materials, Woike & Kanngiesser, 2019b). In the first group (64 comments), participants viewed the lower payment as the simpler option and as being free of potentially negative consequences, and in the promise conditions, some wanted to avoid a commitment. In the second group (120 comments), participants in all conditions referred to self-regarding preferences and free choice when justifying payback of less than $0.05; participants in the control condition saw no reason to repay money. In the third group (381 comments), the majority of participants in the promise or ask conditions referred to norms or the entered covenant when justifying paying back ≥$0.05; some also referred to moral considerations or personal values. Participants in the control condition referred to monetary reasons, norms, and morals.

**Table 1.** Percentages of justifications for decisions in Study 1a.

**Group**	**Reason**	**Promise**		**Ask**	**Control**
		**(write)**	**(click)**		
Rejected 15	Avoid consequences	48	47	82	71
	Simpler option	39	35	9	43
	No commitment	4	29	0	0
	Other	13	6	9	14
Paid back <$0.05	Self-regard	33	56	36	44
	Free choice	50	33	45	8
	No reason	0	0	9	35
	Other	0	0	9	9
Paid back ≥$0.05	Norms	70	74	85	18
	Morals	14	14	12	24
	Monetary reason	8	9	7	32
	Personal values	20	13	9	6
	Other	5	4	7	21

*Note*. Justifications could be scored in more than one category. Percentages are relative to the number of valid comments.

## STUDY 1B

In this study, participants were asked to retrodict the acceptance and the payback decisions in Study 1a in an incentivized paradigm.

### Methods

#### Participants

All participants were located in the United States and recruited through MTurk. Double participation was prevented by a combination of using the Unique Turker script, checking against exclusion lists at the beginning of the survey, and carrying out manual checks of MTurk IDs and IP addresses. After these steps, there were *n*_1*b*_ = 397 participants in Study 1b (*M*_*age*_ = 33.9, 53.9% female, 45.3% male, 0.8% other). All participants gave their informed consent and the study was approved by the IRB at the Max Planck Institute for Human Development.

#### Procedure

We asked participants to retrodict the observed rates for the acceptance and the payback decisions in Study 1a. In a between-subjects design, participants estimated decisions for either the promise (write) condition (*n* = 203) or the control condition (*n* = 193). Participants were incentivized: They were told that estimates close to the observed values would lead to an increase in bonus money (see the Supplemental Materials, Woike & Kanngiesser, 2019b).

### Results

#### Estimation: Acceptance Decision

**Figure 2.**
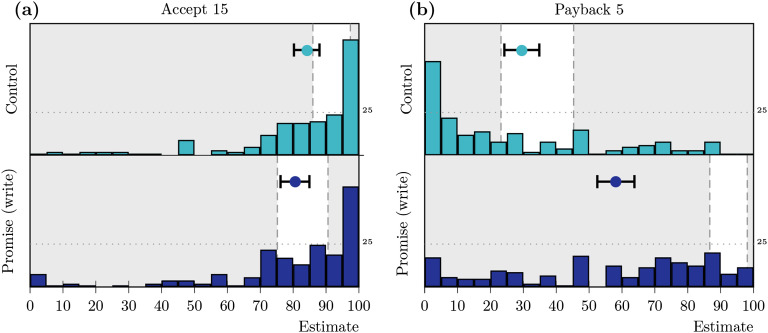
**Distribution, means, and confidence intervals (CIs) for the (a) estimated acceptance and (b) estimated payback rates in Study 1b.** The histograms show the distributions of individual estimates, the markers represent mean estimates, and whiskers correspond to the 99% CI for the mean. The nonshaded areas mark the CIs for the observed rates in Study 1a.

Estimations of the acceptance rates are summarized in Figure 2a. In the promise (write) condition, participants estimated, on average, acceptance rates of *â*_*pw*_ = 0.81 (99% CI = [0.76, 0.85]), *n* = 203) and in the control condition of *â*_*c*_ = 0.84 (99% CI = [0.80, 0.88]), *n* = 193). Average expectations thus aligned well with the observed behavioral data in Study 1a.

#### Estimation: Payback Decision

On average, participants estimated a payback rate of ${\hat{b}}_{c}$ = 0.30 (99% CI = [0.24, 0.35], *n* = 192) in the control condition, which aligned well with our results in Study 1a (see Figure 2b, top). In contrast, participants underestimated the observed payback rates in the promise (write) condition by more than 30%, with ${\hat{b}}_{pw}$ = 0.58 (99% CI = [0.53, 0.64], *n* = 192); many participants gave low estimates for promise-keeping rates (see Figure 2b, bottom).

## STUDY 2A

We conducted a study with a new group of participants to replicate and extend the findings of Study 1a. Specifically, we manipulated the stakes by a factor of 10 for both conditions: Participants were offered a (low-stake) choice between $0.05 and $0.20 with a possible payback of $0.10 (**20–10**) or a (high-stake) choice between $0.50 and $2.00 with a possible payback of $1.00 (**200–100**). Previous work has shown that stake sizes had no effect on promise-keeping rates in a social dilemma (van den Assem et al., 2012); here we investigated whether this would also be the case for promises in our novel, minimal promising paradigm. We used the same general setup as in Study 1a, but included only the promise (write) condition (henceforth: promise condition) and the control condition.

### Methods

#### Participants

All participants were located in the United States and recruited through MTurk. Double participation was prevented by a combination of using the Unique Turker script, checking against exclusion lists at the beginning of the survey, and carrying out manual checks of MTurk IDs and IP addresses. After these steps, there were *n*_2*a*_ = 846 participants in Study 2a (*M*_*age*_ = 33.1, 52.6% female, 47.4% male). All participants gave their informed consent and the study was approved by the IRB at the Max Planck Institute for Human Development.

#### Procedure

To test for effects of stake size, we manipulated the stakes by a factor of 10 for both conditions: Participants were offered a (low-stake) choice between $0.05 and $0.20 with a possible payback of $0.10 (**20–10**) or a (high-stake) choice between $0.50 and $2.00 with a possible payback of $1.00 (**200–100**). This resulted in a 2 (control vs. promise) × 2 (low-stake vs. high-stake) between-subject design. Sample sizes were again set for reasonably accurate estimates; they ranged from 209 to 214. We added a short postquestionnaire to test comprehension of the task and elicited justifications through a set of questions. Participants responded to the PANAS scale (Crawford & Henry, 2004) with items in randomized order.

### Results

#### Acceptance Decision

**Figure 3.**
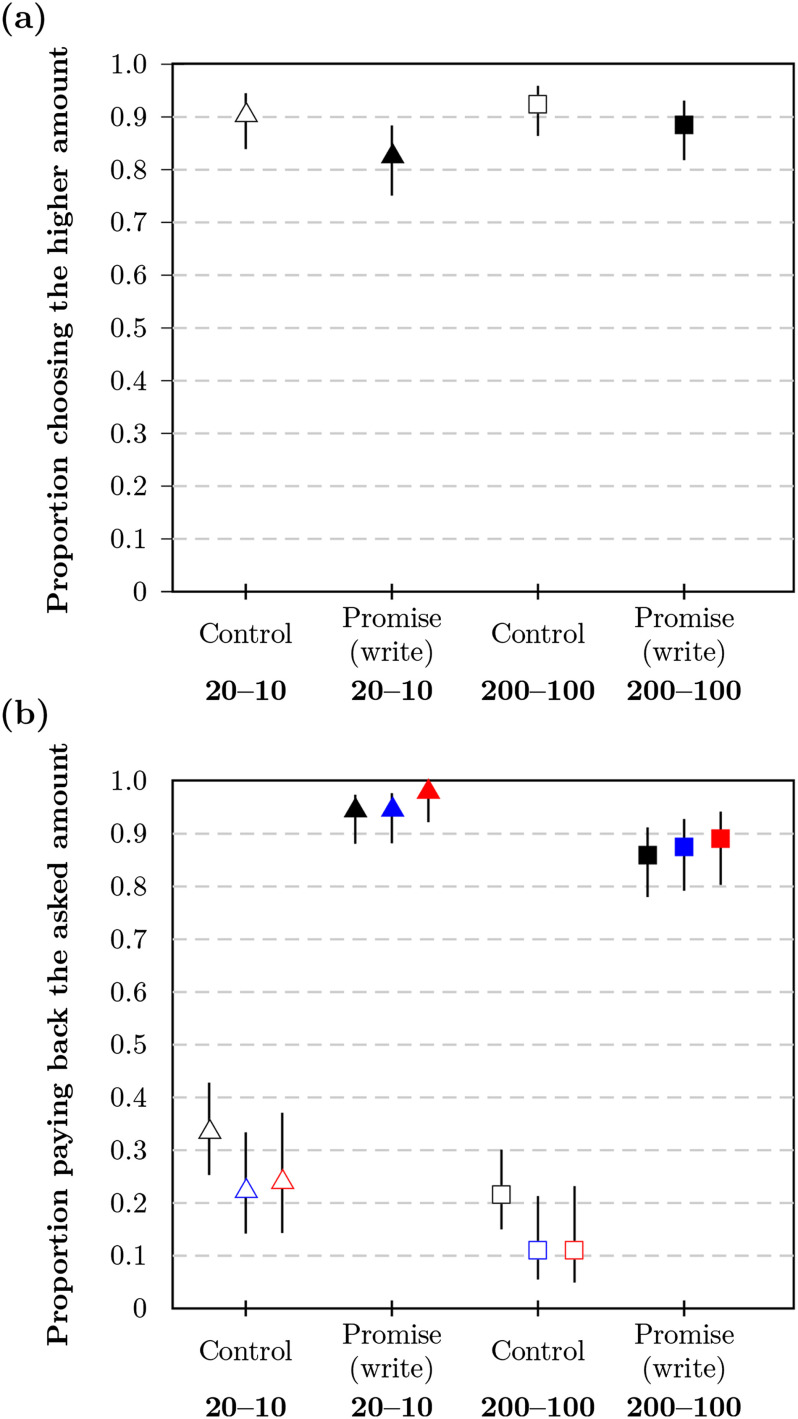
**Proportion of participants (a) accepting the higher payment and (b) paying back at the end in Study 2a.** Proportions are shown as markers for each condition with bars corresponding to the 99% CI (confidence interval). The payback proportion is relative to the number of participants accepting the higher amount. Payback rates are presented with different filters applied based on comprehension check performance: no filter (black), lenient filter (blue), and strict filter (red).

In the promise condition, acceptance rates were *a*_*pl*_ = 0.83 (99% CI = [0.75, 0.88], *n* = 214) for low stakes and *a*_*ph*_ = 0.89 (99% CI = [0.82, 0.93], *n* = 211) for high stakes (see Figure 3a). In the control condition, acceptance rates were *a*_*cl*_ = 0.90 (99% CI = [0.84, 0.95], *n* = 209) for low stakes and *a*_*ch*_ = 0.93 (99% CI = [0.86, 0.96], *n* = 212) for high stakes. Overall, these rates were comparable to the respective rates in Study 1a.

#### Comprehension Checks

Participants who had accepted the higher amount were asked three comprehension questions to assess whether they (1) correctly recalled if they had promised or not, (2) correctly calculated the consequences of their decisions for their bonus payment, and (3) understood that real money was at stake and paid back to the requester, not taken from or given to another participant. The first three rows of Table 2 show the percentage of correct responses per condition; the most common error was to regard the money that was paid back as imaginary. As there is some genuine ambiguity as to whether virtual transactions involve “real” money (Houser & Xiao, 2015), we report three variants of the payback results: (a) using a strict filter excluding all participants with false answers, (b) using a lenient filter allowing participants who viewed money as imaginary (but had answered questions 1–2 correctly), and (c) unfiltered results.

**Table 2.** Percentage of correct answers to the comprehension questions in Study 2a

Check	**Control**		**Promise**	
	$0.20	$2.00	$0.20	$2.00
(1) promise	71.1	68.6	100.0	100.0
(2) own bonus	94.7	82.0	97.2	89.7
(3) real money	62.6	64.4	84.2	80.4
(1–2) correct	64.7	55.7	94.9	87.0
(1–3) correct	47.1	41.8	81.9	74.5

#### Payback Decision

Results for the three filter methods were similar (note that filtering led to somewhat lower payback rates for controls and to somewhat higher payback rates for promises; see Figure 3b). Here, we report the results for the strict filter. In the promise conditions, the majority of participants paid back the money; payback rates were 9% higher in the low-stake condition (*b*_*pl*_ = 0.98, 99% CI = [0.92, 0.99], *n* = 145) than in the high-stake condition (*b*_*ph*_ = 0.89, 99% CI = [0.80, 0.94], *n* = 137) with *δ*_*hl*_ = 0.09 (99% CI = [0.01, 0.18]). In the control conditions, participants’ payback rates were low; payback rates were 13% higher in the low-stake condition (*b*_*cl*_ = 0.24, 99% CI = [0.14, 0.37], *n* = 88) than in the high-stake condition (*b*_*ch*_ = 0.11, 99% CI = [0.05, 0.23], *n* = 81) with *δ*_*hl*_ = 0.13 (99% CI = [−0.03, 0.27]). Details of the distribution of paid back amounts can be found in the Supplemental Materials (Woike & Kanngiesser, 2019b; Figure S33).

#### Affect

To preserve power, we report the analysis with the lenient filter applied (see the Supplemental Materials, Woike & Kanngiesser, 2019b, for further analyses). For negative affect, we observed significant main effects of condition [*F*(1, 549) = 12.16, *p* < .001, partial η^2^ = .02] and payback (yes/no) [*F*(1, 549) = 33.96, *p* < .001, partial η^2^ = .06], but not of stakes [*F*(1, 549) = 0.10, *p* = .76, partial η^2^ = .00]. The only significant interaction was between condition and payback [*F*(1, 549) = 3.97, *p* = .047, partial η^2^ = .01]. Average scale values are about 2 points higher in the promise versus control condition, and 3.3 points higher for participants who failed to pay money back. This difference was higher in the promise (*δ* = 4.5) than in the control condition (*δ* = 2.2). For positive affect, we observed a significant main effect of payback [*F*(1, 549) = 10.35, *p* = .001, partial η^2^ = .02], but no significant effects of condition [*F*(1, 549) = 0.05, *p* = .83, partial η^2^ = .00] or stakes [*F*(1, 549) = 0.76, *p* = .38, partial η^2^ = .00]. There was no significant interaction, although the interaction between condition and payback was similar to the one for negative affect [*F*(1, 549) = 3.55, *p* = .06, partial η^2^ = .01]. Participant who paid money back had a scale value about 4.4 points higher, which was more pronounced in the promise (*δ* = 6.9) than in the control condition (*δ* = 1.8).

#### Reasons

Participants answered a set of quantitative questions on the reasons for their decisions, based on condition and decision type (see the Supplemental Materials, Woike & Kanngiesser, 2019b, for details). (1) Participants who rejected the higher payment, affirmed, on average, that they preferred a certain and simpler option, and to some degree that they feared negative consequences of claiming more. (2) Participants who did not pay money back in the control conditions indicated that they saw no reason for doing so, and felt to some degree entitled because they had worked on the survey. (3) Participants who broke their promise similarly referred to a lack of a reason for paying money back, as well as to the lack of enforcement. They also felt their hard work entitled them to the money and affirmed a selfish motivation. (4) Participants who kept their promise affirmed, on average, that there is an obligation to keep promises, that they saw themselves as someone who keeps promises, and that they wanted to do the right thing. They rejected, on average, that they kept their promise because the amounts were too small. (5) Participants who paid money back in the control conditions affirmed that they wanted to be nice to the requester or considered themselves to be generous. They did not expect to earn more money as a consequence.

## STUDY 2B

We asked a separate group of participants to estimate payback rates for Study 2a by specifying their subjective probability distribution over possible intervals. Responses were incentivized using the quadratic scoring rule (Harrison, Martínez-Correa, Swarthout, & Ulm, 2017; Matheson & Winkler, 1976).

### Methods

#### Participants

All participants were located in the United States and recruited through MTurk. Double participation was prevented by a combination of using the Unique Turker script, checking against exclusion lists at the beginning of the survey, and carrying out manual checks of MTurk IDs and IP addresses. After these steps, there were *n*_2*b*_ = 1,384 participants in Study 2b (*M*_*age*_ = 35.6, 56.9% female, 43.0% male, 0.1% other). All participants gave their informed consent and the study was approved by the IRB at the Max Planck Institute for Human Development.

#### Procedure

Participants were asked to retrodict the proportion of participants paying back at least the requested amount in the four conditions of Study 2a, that is, the low-stake (**20–10**) and high-stake (**200–100**) conditions in both the promise and control variants. In contrast to Study 1b, participants were incentivized using the quadratic scoring rule: Participants allocated 100 points across 22 intervals (all 5% intervals between 5% and 95% and a split of the smallest and highest intervals into 4.99% and the extreme 0.01%), resulting in relative allocation of *r*_1_ to *r*_22_ with ∑_*i*_ *r*_*i*_ = 1. Participants received bonus money based on their allocations to the correct interval *r*_*c*_ and false intervals *r*_*i*≠*c*_, with a payoff of *o* = (2*r*_*c*_ − ∑_*i*≠*c*_*r*_*i*_^2^) ⋅ $0.50. Procedure and payment consequences were explained to participants in a multistep tutorial (see the Supplemental Materials, Woike & Kanngiesser, 2019b, also for demonstrations of the consequences).

#### Data Analyses

We considered participants with intermittent gaps in their allocation to intervals as inconsistent and analyzed the data with and without this group.

### Results

#### Expectations

**Figure 4.**
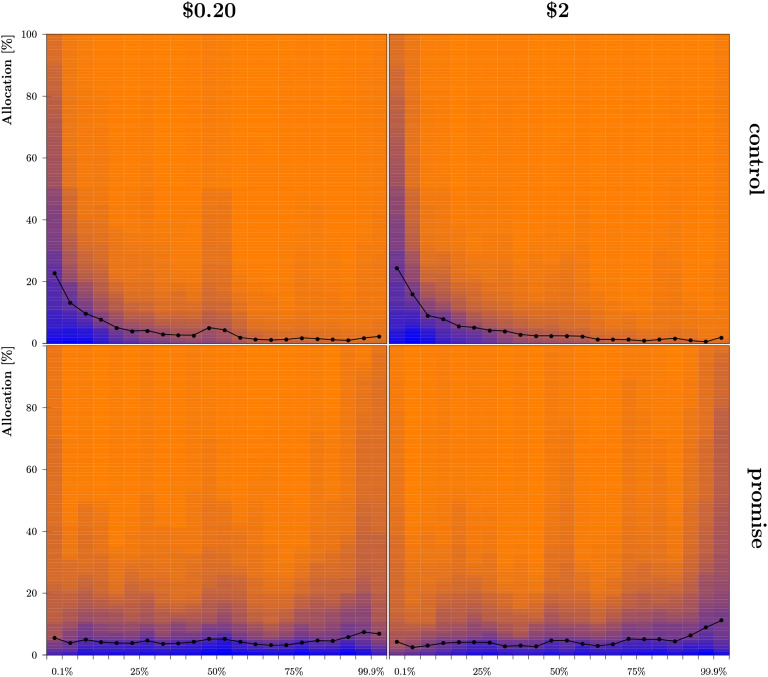
**Distribution and means for individual subjective probability distributions in Study 2b for low stakes (left) and high stakes (right) crossed with control (top) and promise (bottom) conditions.** Markers correspond to the average allocations to each interval; blue shading corresponds to the number of participants who committed at least the shaded amount of points to the respective interval.

**Figure 5.**
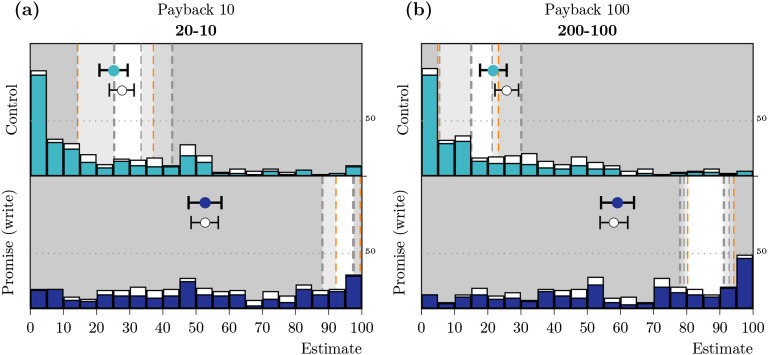
**Distribution, means, and confidence intervals (CIs) for estimated payback rates in Study 2b.** The histogram shows the distribution of individual estimates for both promise conditions (top and bottom) for (a) low stakes and (b) high stakes. Histograms represent the distributions of calculated individual point estimates. The markers show the mean estimate and whiskers correspond to the 99% CI for the mean (the filled marker for consistent participants, the unfilled marker for all participants). Different degrees of shading delimit the CIs for the corresponding rates observed in Study 2a, with the interval marked by orange lines for the strict filter, black for the lenient filter, and gray for no filter.

Most participants’ allocations were classified as consistent (*π*_*all*_ = 0.78, with 0.76 < *π*_*i*_ < 0.81 across conditions). Distributions and average allocations to intervals are summarized in Figure 4. Individual estimates were calculated by summing the products of relative allocations and interval midpoints, resulting in the distribution and means summarized in Figure 5. Filtered results were similar to unfiltered results (see the Supplemental Materials, Woike & Kanngiesser, 2019b). Participants estimated payback rates in the control conditions as ${\hat{b}}_{cl}$ = 0.25 (99% CI = [0.21, 0.30], *n* = 278) for low stakes and ${\hat{b}}_{ch}$ = 0.22 (99% CI = [0.17, 0.26], *n* = 265) for high stakes. The average estimates for the control conditions corresponded well to the observed rates in Study 2a, similar to Study 1. In the promise conditions, participants again underestimated payback rates by about 40% for low stakes, ${\hat{b}}_{pl}$ = 0.53 (99% CI = [0.48, 0.57], *n* = 267), and by about 20% for high stakes, ${\hat{b}}_{ph}$ = 0.59 (99% CI = [0.55, 0.64], *n* = 267). The estimated rate was higher for promise high stakes than for promise low stakes (*δ* = 0.06, 99% CI = [0.02, 0.11]), with the observed rates differing in the opposed direction (see Figure 3).

## STUDY 3

We conducted a further study with a new group of participants to test the robustness of the promising-keeping rates in Studies 1a and 2a. In this study, we manipulated two new factors and included additional robustness checks. First, we manipulated the visibility of promise-keeping or promise-breaking. Participants were divided evenly between a clear condition and an obfuscation condition. In the obfuscation condition, participants played a guessing game similar to the task employed in Jiang (2013): They were asked to guess a number between 0 and 9 (without indicating their guess) and were then shown a randomly generated number. Participants whose guess matched the number were instructed to pay back zero cents in their final decision. Participants in the obfuscation condition thus knew that a payback of zero was ambiguous for the experimenter: There was no way of knowing whether they had guessed correctly. The manipulation made it possible to assess the impact of potential concerns about reputation and negative consequences for future work opportunities on MTurk on payback decisions. Second, we varied whether the target of the payback was the experimenter (as in Studies 1a and 2a) or another participant (another MTurk worker). Specifically, in the experimenter condition participants were told that the money would be paid back to the experimenter; in the peer condition, they were told the money they paid back would go to a future participant. This allowed us to test whether the experimenter’s authority had any influence on participants’ payback decisions. All manipulations were run in a promise and a control condition (without a promise). This resulted in a 2 (obfuscation vs. clear) × 2 (experimenter vs. peer) × 2 (promise vs. control) between-subject design.

We used the same payment amounts as in the low-stake conditions of Study 2a (**20–10**) and presented the promise option in the (weaker) click-only variant from Study 1a. In addition, we introduced the following changes: (i) To reduce possible demand effects, we eliminated the reminder of the choice and promised/suggested amounts of payback before participants made a decision about paying money back. We also clearly stated that there would be no further changes to the bonus payment (e.g., surprise payments) after their decision, and explained in all conditions that our analysis would focus on the group level, not on particular individuals. (ii) To increase the sense of endowment for the chosen amount of money, we displayed how much bonus money participants currently owned on each page of the survey. We preregistered the study before data collection on the OSF platform (Woike & Kanngiesser, 2019a).

### Methods

#### Participants

All participants were located in the United States and recruited through Amazon’s Mechanical Turk. In reaction to current data quality concerns (Kennedy, Clifford, Burleigh, Waggoner, & Jewell, 2018), we followed the procedures preventing the circumvention of location requirements suggested by Burleigh, Kennedy, and Clifford (2018) and also included specific tests (Moss & Litman, 2018) of language competence (see the Supplemental Materials, Woike & Kanngiesser, 2019b). Double participation was prevented by a combination of using the Unique Turker service, adding qualifications to exclude previous participants, and carrying out manual checks to ensure the uniqueness of MTurk IDs and IP addresses. After these steps, there were *n*_3_ = 1,001 participants in Study 3 (*M*_*age*_ = 36.6, 46.6% female, 53.3% male, 0.1% other). A separate group of *n*_3*p*_ = 257 participants were passive recipients of bonus transfers in the peer conditions. All participants gave their informed consent and the study was approved by the IRB at the Max Planck Institute for Human development.

#### Procedure

All participants were offered a choice between $0.05 and $0.20 with a suggested/promised payback of $0.10 (**20–10**). We varied three factors in this study (see the Supplemental Materials, Woike & Kanngiesser, 2019b). First, half of the participants played a guessing game (obfuscated condition), the other half did not (clear condition). Participants in the obfuscated condition mentally guessed a number between 0 and 9 and were then shown a randomly drawn number. In case of a match (labeled “winning the game”), participants were instructed to not pay back any money at the end of the survey. The procedure ensured that it was public knowledge that only the participants themselves could know whether a match had occurred. Second, the payback target was either the experimenter (as in all previous studies) or a peer. In the peer condition, participants were paired with future participants on MTurk (members of a separate group) and their decisions had consequences for both participants’ payoffs. Choosing the lower payoff resulted in $0.05 for both participants. Choosing the higher payoff resulted in a total bonus amount of $0.20 for the current participant with a chance to pay back any amount at the end the study. Participants were informed that the future participants would receive a protocol of their decisions (including promises made and, in the obfuscated condition, the existence and possible consequences of the guessing game). Third, participants were assigned to a control condition (the same as in Study 2a) or a promise condition (the promise [click] condition in Study 1a). This resulted in a 2 (obfuscation vs. clear) × 2 (experimenter vs. peer) × 2 (promise vs. control) between-subject design.

There were 123 to 126 participants per cell of the design. In contrast to the previous studies, there was no reminder of promises or suggested/promised payback amounts at the end of the study, and a participant’s current bonus amount was displayed at the top of their screen throughout the study. We added a short postquestionnaire to test the success of the promise and target manipulations (see the Supplemental Materials, Woike & Kanngiesser, 2019b).

### Results

#### Acceptance Decision

**Figure 6.**
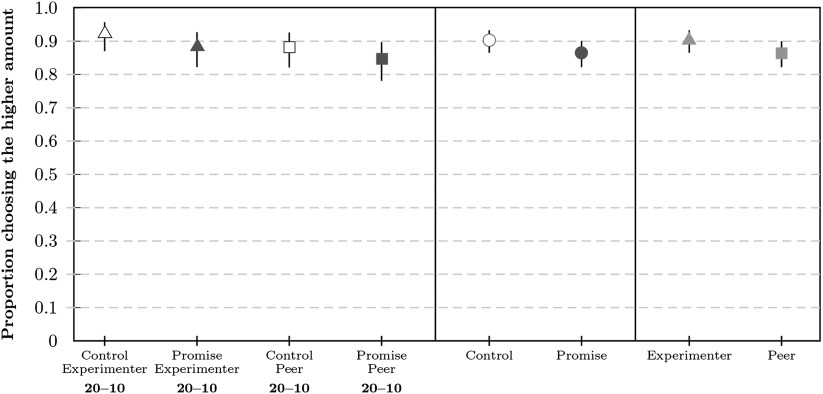
**Proportion of participants in Study 3 accepting the higher amount of $0.20 in the beginning, split by individual conditions and factor levels.** Proportions are shown as markers for each condition with bars corresponding to the 99% CI (confidence interval).

Since the guessing game occurred after the acceptance decision, acceptance rates were collapsed for the obfuscation/clear condition. In the promise condition, acceptance rates were *a*_*pe*_ = 0.88 (99% CI = [0.82, 0.93], *n* = 251) for the experimenter target and *a*_*pp*_ = 0.85 (99% CI = [0.78, 0.90], *n* = 250) for the peer target. In the control condition, acceptance rates were *a*_*ce*_ = 0.92 (99% CI = [0.87, 0.96], *n* = 251) for the experimenter target and *a*_*cp*_ = 0.88 (99% CI = [0.82, 0.93], *n* = 249) for the peer target (see Figure 6).

#### Manipulation Checks

Participants who had accepted the higher amount were asked two manipulation questions to assess whether they (1) correctly recalled if they had promised or not, and (2) understood that real money was at stake and remembered who the target of the payback decision was. Among the 886 participants who had accepted the higher amount, 741 participants (74%) correctly answered the first question and 757 participants (75.6%) correctly answered the second question; 639 participants (63.8%) correctly answered both questions. Results for the payback decision were calculated for the unfiltered and filtered sample (restricted to the group with two correct answers), respectively.

#### Payback Decision

**Figure 7.**
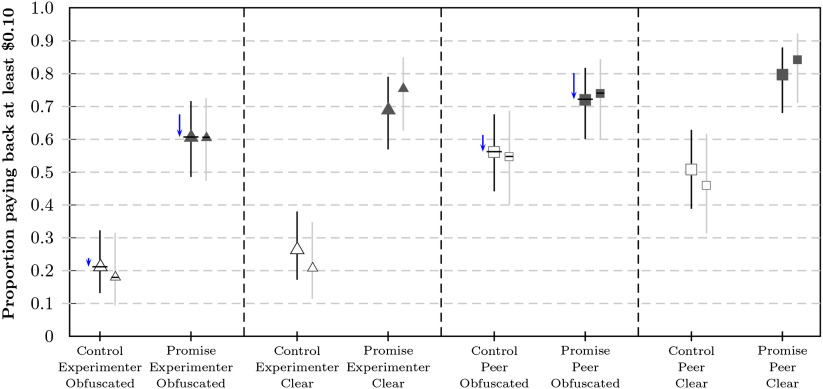
**Proportion of participants paying back at least $0.10 in Study 3 for all conditions.** Proportions are shown as markers for each condition with bars corresponding to the 99% CI (confidence interval). The payback proportion is relative to the number of participants accepting the higher amount. Payback rates are presented unfiltered (larger markers) and filtered (smaller markers). Arrowheads mark the estimated rates of payback when replacing expected zero payments (10% of participants) with observed rates from the corresponding clear conditions.

**Figure 8.**
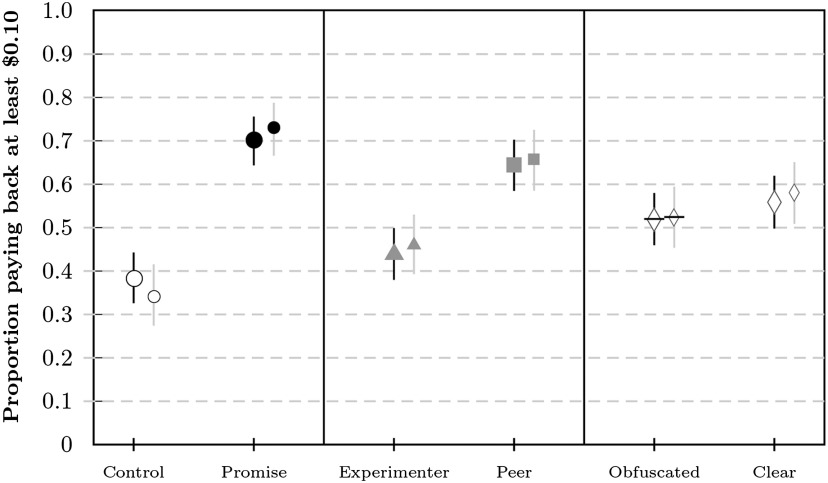
**Proportion of participants paying back at least $0.10 in Study 3 for factor levels.** Proportions are shown as markers for each condition with bars corresponding to the 99% CI (confidence interval). The payback proportion is relative to the number of participants accepting the higher amount. Payback rates are presented unfiltered (larger markers) and filtered (smaller markers).

Results for the filtered and unfiltered sample were similar (note that filtering resulted again in somewhat lower payback rates in the control and somewhat higher payback rates in the promise conditions; see Figure 7 and Figure 8). Here we report the results for the filtered sample split by factor levels (for details on the distribution of paid-back amounts, see the Supplemental Materials (Woike & Kanngiesser, 2019b; Figure S33)), and higher in the promise conditions (*b*_*p*_ = 0.73, 99% CI = [0.67, 0.79], *n* = 346) than in the control conditions (*b*_*c*_ = 0.34, 99% CI = [0.27, 0.42], *n* = 293), with *δ*_*pc*_ = 0.39 (99% CI = [0.29, 0.48]). Payback rates were higher in the peer conditions (*b*_*pe*_ = 0.66, 99% CI = [0.59, 0.73], *n* = 296) than in the experimenter conditions (*b*_*ex*_ = 0.46, 99% CI = [0.39, 0.53], *n* = 343), with *δ*_*peex*_ = 0.20 (99% CI = [0.10, 0.29]), with a larger difference in the control conditions. Payback rates in the control conditions were higher than average donation rates in dictator games (about 29.8% in the meta-analysis conducted by Engel (2011), which might be due to our framing of taking bonus money from other participants (Oxoby & Spraggon, 2008).

Payback rates in the clear conditions (*b*_*cl*_ = 0.58, 99% CI = [0.51, 0.65], *n* = 313) were slightly higher than those in the obfuscated conditions (*b*_*ob*_ = 0.52, 99% CI = [0.45, 0.59], *n* = 326), with *δ*_*clob*_ = 0.06 (99% CI = [−0.04, 0.16]). However, an analysis of individual conditions showed that the difference between clear and obfuscated conditions deviated only marginally from the expected difference—that is, if those participants who had won the guessing game returned zero and everyone else returned at the average rate observed in the corresponding clear conditions (see Figure 7). Interestingly, some participants voiced tensions in reconciling their promise to pay back with the instructions not to pay back after winning the guessing game in their comments at the end of the survey (see the Supplemental Materials, Woike & Kanngiesser, 2019b).

## GENERAL DISCUSSION

We tested a minimal, fully incentivized method to measure promise-keeping and found across three studies that the majority of participants (61%–98%) in an online labor market kept their promises at a financial cost to themselves. Promises thus exert a strong normative force, even in the face of monetary temptations. Surprisingly, we found that different groups of participants, asked to estimate promise-keeping rates in Studies 1b and 2b, systematically underestimated promise-keeping by 20%–40%. This behavior–estimation gap could mean that people lose out on chances to cooperate and coordinate with others because they are overly pessimistic about the power of others’ words.

The effect of promises on behavior has been studied in a wide range of situations and paradigms (Bicchieri, 2002; Carlsson et al., 2013; Carlsson & Kataria, 2018; Charness & Dufwenberg, 2006; Ellingsen & Johannesson, 2004; Heyman, Fu, Lin, Qian, & Lee, 2015; Jacquemet, Luchini, Rosaz, & Shogren, 2019; Kanngiesser, Köymen, & Tomasello, 2017; Kataria & Winter, 2013; Orbell et al., 1988; Ostrom et al., 1992; Sally, 1995; van den Assem et al., 2012; Wang & Katzev, 1990). Previous studies have found that the context of promising matters: Promises are more effective in dyadic than in group settings (Lev-On, Chavez, & Bicchieri, 2010) and more effective in face-to-face communication than in computer-mediated interactions (Bicchieri & Lev-On, 2007; but see Conrads & Reggiani, 2017), and are interpreted differently in competitive contexts (Casella, Kartik, Sanchez, & Turban, 2018). We found in Study 3 that the type of target of a promise had an impact as well: Promise-keeping rates were higher for an unidentified peer (another MTurk participant) than for a presumed authority figure (the experimenter)—though it should be noted that participants in the control conditions also shifted toward more generous paybacks for the peer. Importantly, we can consistently show across three studies that promises were highly effective in a minimal setting where neither the promisor nor the promisee knew each other, had face-to-face contact, or exchanged personal messages. Even in situations in which participants were not reminded of their promise and in which breaking a promise was obfuscated through a guessing game (Study 3), the majority of participants (61%–74%) kept their word. In light of this, our minimal promise situations can be considered a conservative test of people’s promise-keeping.

We found in Study 1a that the commitment format did not affect promise-keeping: Promise-keeping rates were equally high for participants who simply clicked on a prewritten promise (promise [click]: 94%) and for participants who wrote a minimal promise themselves (writing “I promise”: 95%). It was only when the word “promise” was omitted altogether (ask condition) that payback rates decreased. Previous research has found that prewritten promises had less of an effect on increasing cooperation than free-form messages (Charness & Dufwenberg, 2006, 2010). However, sample sizes in these two studies were considerably smaller than in our study.^1^ Also, the two studies were not run simultaneously and rates of promise-making differed substantially between these groups,^2^ making it likely that different pools of participants were compared. Another study found that unconditional and explicit statements were associated with higher cooperation rates in a TV show than conditional or implicit statements (Turmunkh et al., 2019). Yet the types of statements varied considerably and included wishes and desires (e.g., “I want us to do X”), which we would not count as promises. It is, in our view, perfectly intelligible to say: ”I intend to do X, but I cannot promise it.” Future research could use our novel paradigm to measure promise-keeping for different promise formats such as conditional or unconditional promises, or to compare promises and mere statements of intent.

Varying stake sizes in Study 2a ($0.20 vs. $2.00) did not result in noticeable differences in promise-keeping rates. A recent meta-analysis on stake sizes in economic games found that these effects were game-specific and somewhat inconsistent across studies—for example, small average effects were found for dictator games, but not for ultimatum games (Larney, Rotella, & Barclay, 2019). Research with participant samples similar to the ones in our studies (U.S. participants on MTurk) showed clear differences between small stakes and no stakes in dictator games (Amir, Rand, & Gal, 2012), but virtually no differences when raising stakes above $1.00 (Raihani, Mace, & Lamba, 2013). Our choice of stake sizes of up to $2.00 appears appropriate to produce generalizable findings with MTurk participants.

Participants justified their promise-keeping by referring to promise norms and the obligation to keep one’s word. As one participant in Study 1a stated: “Mutual agreements are the basis of social contracts. A social agreement, once agreed to, ought to be binding regardless of the circumstances—with very rare exceptions indeed. Once I had agreed, it’s true that there was no force upon me to bind me, except my own moral values–but because I believe in the necessity of living up to one’s word, and I wanted to remain consistent within myself, I chose to honor the agreement that I had made.” These types of justifications appear more consistent with accounts of promise-keeping that assume a preference for promise-keeping (Ellingsen & Johannesson, 2004; Vanberg, 2008) than with accounts that assume an aversion to disappoint others’ expectations (Charness & Dufwenberg, 2006; Scanlon, 1990). While participants’ justifications indicate that promise-keeping mattered to them, we cannot rule out that an aversion to (or guilt about) disappointing others’ expectations may have also played a role in participants’ decisions to honor their promise. In fact, recent work has shown that promise-keeping could be driven by both a preference to keep one’s promise and expectation-based guilt aversion (Bhattacharya & Sengupta, 2016; Di Bartolomeo et al., 2018; Ederer & Stremitzer, 2017; Kolodny & Wallace, 2003; Mischkowski et al., 2016). In line with both explanations, paying money back was on average accompanied by more positive and less negative affect than keeping it.

Participants in our study systematically underestimated payback rates in the promise conditions, but accurately estimated them in the control conditions, indicating that participants were, in principle, able to accurately predict others’ behavior. Estimations of promise-keeping rates were characterized by a high degree of variability, demonstrating that a vast majority of participants did not have an accurate expectation of promise-keeping; this is surprising given the observed self-binding power of promises in Studies 1a and 2a and the fact that estimators had access to the full description of conditions. A positive bias toward one’s own versus others’ morality (Pronin, 2008) may only partially explain why people were pessimistic about others’ promise-keeping. Moreover, our findings cannot be explained by the “false consensus bias” that has been observed for beliefs about dictator game contributions (Iriberri & Rey-Biel, 2013), as this bias would lead only the small minority that breaks promises to underestimate promise-keeping rates. Other studies have produced inconsistent results regarding participants’ estimations of others’ trustworthiness or promise-keeping rates: Participants underestimated the general trustworthiness of trustees in trust games without promises (Fetchenhauer & Dunning, 2010) and underestimated the effect of voluntary promises on cooperation in game show videos by a magnitude (Belot et al., 2012), but overestimated the trustworthiness of written promises or statements of intent in a trust game (Chen & Houser, 2017). However, estimations in all these studies were based on small samples. Moreover, participants were asked to judge specific interactions or messages and it is thus possible that situational cues (e.g., nonverbal cues, wording) affected estimation rates.

In Study 3 we tested the robustness of promise-keeping rates. We found that promise-keeping rates were lower overall than in Studies 1a and 2a, which included a reminder of the initial promise. It is possible that the reminder, which was included to rule out memory errors, increased promise-keeping rates—either because it reminded people who would have otherwise forgotten or because it highlighted the commitment, or both. We further found relatively high amounts of payback in the control conditions with peer targets in Study 3. Participants on MTurk have been observed to show an in-group bias toward other members of crowdsourced platforms (Almaatouq, Krafft, Dunham, Rand, & Pentland, 2018) that is not extended to researchers on the platforms (Semuels, 2018). In addition, our framing of taking bonus money from peers may have increased payback rates in the control conditions (see e.g., Oxoby & Spraggon, 2008). Interestingly, promise-keeping rates were similar in the obfuscation and the clear conditions in Study 3, even though potential recipients in the obfuscation condition were aware of the possibility of a chance-based elimination of payments and neither the recipient nor the experimenter could know the result of the guessing game. This strengthens the argument that promises have a self-binding function that goes beyond conforming to the expectations of others (Ellingsen & Johannesson, 2004; Ismayilov & Potters, 2017; Vanberg, 2008).

Across studies, we incentivized the choice of the promise options in order to measure promise-keeping rates for a large proportion of participants and reduce self-selection of, for example, more cooperative participants. It should be noted that participants who opted out of the promise incurred a loss of money, which is an indication of taking promises seriously rather than a propensity for promise-breaking. Restricting the analysis to participants who did choose to make a promise is therefore likely to yield a conservative measure of promise-keeping. In addition, our paradigm is straightforward and simple to implement and opens many avenues for future research on promise-keeping, such as using promises to induce more ethical behaviors.

In conclusion, we found that most people chose the integrity of their word over easy personal financial gain and did so more often than others would expect. Thus, there may well exist a yet-untapped potential for promises to establish and facilitate coordination and cooperation.

## ACKNOWLEDGMENTS

We thank two anonymous reviewers, Yaakov Kareev, Ryan Murphy, Zoe Rahwan, and members of the Center of Adaptive Rationality at the Max Planck Institute for Human Development for constructive criticism and feedback on the project, and we thank Anna Dania Esch and Manuel Canella for coding the open answers in Study 1a. We thank Deborah Ain for editing this manuscript.

## FUNDING INFORMATION

Patricia Kanngiesser, Volkswagen Foundation, Award ID: 89611.

## AUTHOR CONTRIBUTIONS

JKW: Conceptualization: Equal; Data curation: Lead; Formal analysis: Lead; Methodology: Equal; Software: Lead; Visualization: Lead; Writing – Original Draft: Equal. PK: Conceptualization: Equal; Methodology: Equal; Writing – Original Draft: Equal.

## Notes

^1^ Based on the reported sample sizes, the 99% CI for cooperative choices after bare promises in Charness and Dufwenberg (2010) was [0.41, 0.78] (compared to [0.27, 0.63] in the control condition). Messages containing statements of intent in Charness and Dufwenberg (2006) resulted in a cooperation rate with a 99% CI of [0.61, 0.90], such that Δ_*bm*_ = 0.18, 99% CI = [−0.41, 0.07]), which could be considered limited evidence for the claim that “bare promises had substantially less effect on behavior” (Charness & Dufwenberg, 2010, p. 283).^2^ More than 30% of participants self-selected to make a promise in the bare messages condition as compared to participants classified as stating an intent in the free-form condition.
