# Supplementary material for: Most People Keep Their Word Rather Than Their Money
Source: Open Mind (Camb). 2019 Jul 1;3:68–88. doi: 10.1162/opmi_a_00027 (PMC8412196; doi:10.1162/opmi_a_00027)
Supplement: Supplementary file 1 [file opmi-03-68-s001.pdf]

Supplementary Material (1/4) for "Most People Keep Their Word Rather Than Their Money"

Study 1a, 1b

Jan K. Woike
Max-Planck-Institute for Human Development, Berlin

Patricia Kanngiesser
Freie Universität Berlin

Compiled on May 2nd, 2019

Contents

1 Study 1a: Survey conditions and material 3
1.1 MTurk Specification . . . 3
1.2 Attention checks . . . 3
1.3 Start of the survey . . . 6
1.3.1 Control condition . . . 6
1.3.2 Ask condition . . . 6
1.3.3 Promise (click) condition . . . 6
1.3.4 Promise (write) condition . . . 7
1.3.5 Higher bonus rejected (all conditions) . . . 7
1.4 End of the survey . . . 10
1.4.1 Control condition . . . 10
1.4.2 Ask condition . . . 10
1.4.3 Promise (click) and promise (write) condition . . . 10
1.5 Post-questionnaire . . . 12
1.5.1 Non-promise conditions . . . 12
1.5.2 Promise conditions: promise broken . . . 12
1.5.3 Promise conditions: promise kept . . . 12
1.5.4 All conditions: PANAS scale . . . 12

2 Study 1b: Survey conditions and material 15
2.1 MTurk Specification . . . 15
2.2 Attention checks . . . 15
2.3 Estimation task . . . 17
2.3.1 Both conditions . . . 17

List of Figures

S1 Attention check Study 1a . . . 5
S2 Start of Study 1a: Control . . . 7
S3 Start of Study 1a: Ask . . . 8
S4 Start of Study 1a: Promise (click) . . . 8
S5 Start of Study 1a: Promise (write) . . . 9
S6 End of Study 1a: Promise . . . 11
S7 End of Study 1a: Explanation . . . 13
S8 End of Study 1a: PANAS . . . 14
S9 Attention Check Study 1b . . . 16
S10 Study 1b: Estimation Part 1 . . . 18
S11 Study 1b: Estimation Part 2 . . . 19

1 Study 1a: Survey conditions and material

1.1 MTurk Specification

- **Announced timespan**: 10–20 minutes
- **Fixed compensation**: $1.25
- **Bonus payment**: Up to $ 1.00
- **Time allotted**: 2 hours
- **Auto-approval**: 3 days
- **Filters**: Hit approval rating $\geq$ 95%, Hits > 50, Location: US
- **Attention check:** yes

1.2 Attention checks

Participants had to pass one of two attention checks. The second check was only presented if the first test was failed. See Figure S1 for an example.

> Choices of participants will always be affected by the context of those decisions. Some contexts are less interesting than others. For example, we are not interested in answers that are given in the context of not reading the instructions. Therefore we need to make sure that you are actually taking the time to read these texts. If you read this text then please ignore the following question and do not try to answer it. Instead, just move the last category (knowledge) to the top position and leave everything else unchanged. Thank you very much.
>
> Please order the **following goals in life in order of the importance** that you attach to them (you can drag and drop the rows with your mouse): [*The entries in the list were draggable and could be moved up or down, see Fig. S1*]

1. Fame
2. Achievement
3. Security
4. Happiness
5. Fun
6. Health
7. Wealth
8. Friends
9. Knowledge

If a participant did not follow the instructions, a second test was shown, otherwise they continued to the task.

> Thank you for sharing your goals in life with us. Unfortunately that was not the question we wanted to you to answer. It seems that you have not read the instructions for the last question thoroughly. We will give you a second chance to pass this test. Answer the following question by entering the word instruction without any capitalized letter in the field below, nothing else.
>
> What is your **favorite TV show** at the moment (including documentary formats)? [*Entry box*]

If a participant did not follow the instructions, he would be advised to return the HIT and was given an incorrect password, which was clearly stated in the text. Only participants who submitted incorrect passwords were rejected. Participants who entered "instruction" continued to the main task.

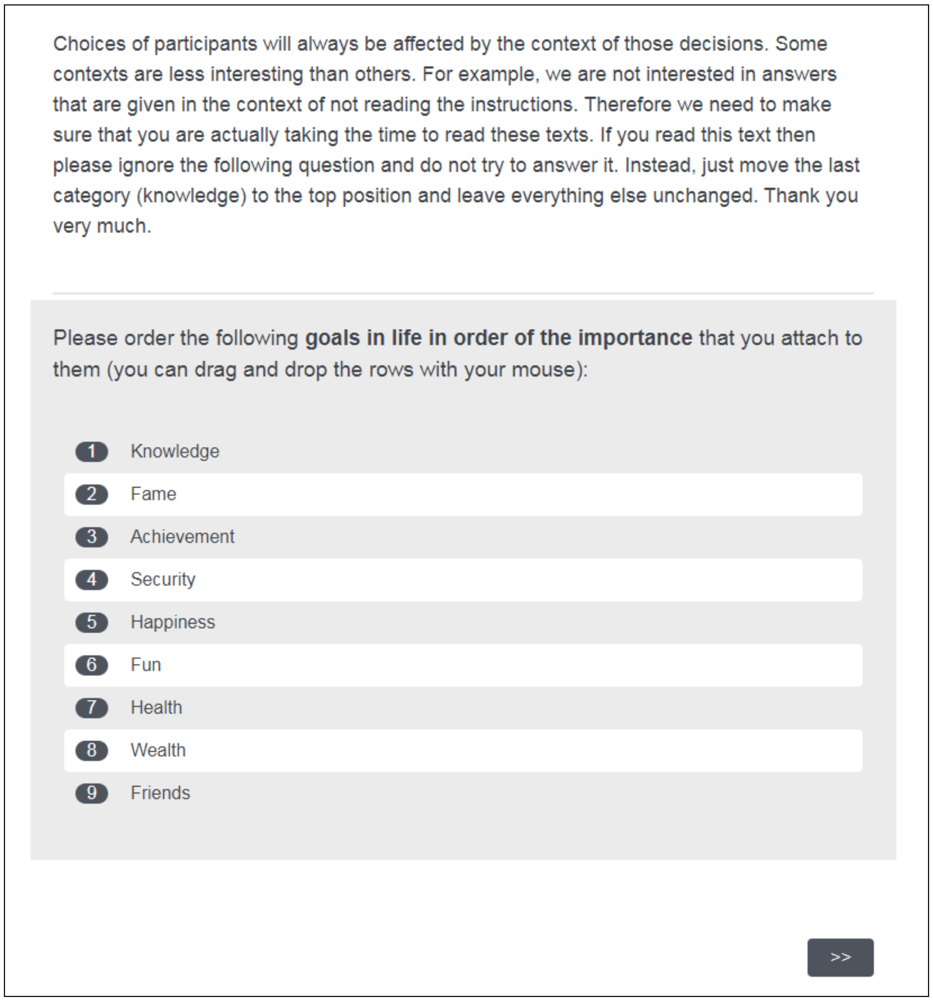

Figure S1. Qualtrics interface of first attention check (Study 1a)

1.3 Start of the survey

1.3.1 Control condition. *See Figure S2 for a screen shot.*

You have two options that have consequences for your bonus payment:

1. You can receive 5 cents without any further consequences.
2. You can receive 15 cents. In this case we would give you a chance to give back 5 cents at the end of the survey. (You will be able to determine how much you want to pay back).

[*Participants were offered two options in check boxes:*]

- I take 5 cents.
- I take 15 cents and I can decide at the end of the survey whether I want to pay back 5 cents.

1.3.2 Ask condition. *See Figure S3 for a screen shot.*

You have two options that have consequences for your bonus payment:

1. You can receive 5 cents without any further consequences.
2. You can receive 15 cents. In this case we would ask you at the end of the survey to give back 5 cents at the end of the survey.

[*Participants were offered two options in check boxes:*]

- I take 5 cents.
- I take 15 cents and I will be asked to pay back 5 cents at the end of the survey.

1.3.3 Promise (click) condition. *See Figure S4 for a screen shot.*

You have two options that have consequences for your bonus payment:

1. You can receive 5 cents without any further consequences.
2. You can receive 15 cents. In this case we would ask you to promise that you will give back 5 cents at the end of the survey. (We would ask you to pay back this money at the end).

[*Participants were offered two options in check boxes:*]

- I take 5 cents.
- I take 15 cents and I promise to pay back 5 cents when asked to at the end of the survey.

1.3.4 Promise (write) condition. *See Figure S5 for a screen shot.*

You have two options that have consequences for your bonus payment:

1. You can receive 5 cents without any further consequences.
2. You can receive 15 cents. In this case we would ask you to promise that you will give back 5 cents at the end of the survey. (We would ask you to pay back this money at the end). *If you choose this option, write "I promise" (without quotation marks) into the text box.*

[*Participants were offered two options in check boxes:*]

- I take 5 cents.
- I take 15 cents and I promise to pay back 5 cents when asked to at the end of the survey. [*Text entry box*]

1.3.5 Higher bonus rejected (all conditions). Please explain why you did not want to make the promise and receive more money. [*Text box*]

[*Participants who reject the higher payment did not encounter the end of survey block*]

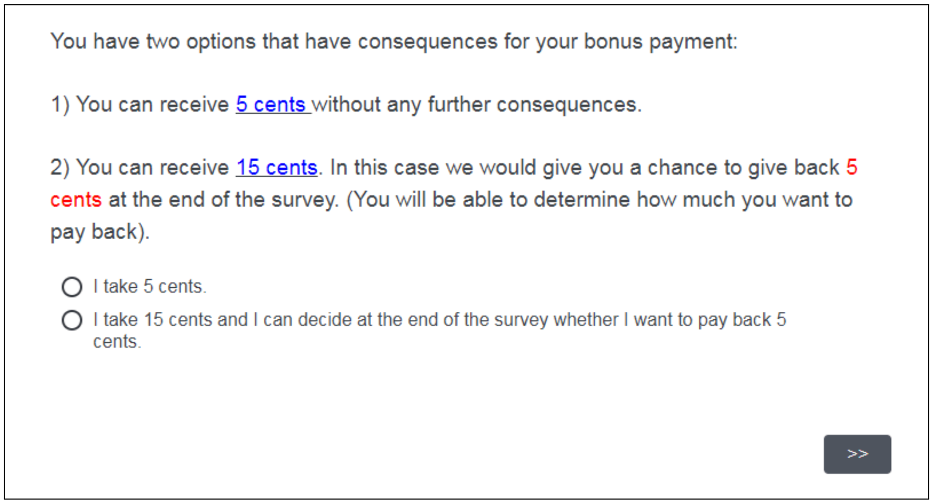

Figure S2. Start of Study 1a in the control condition

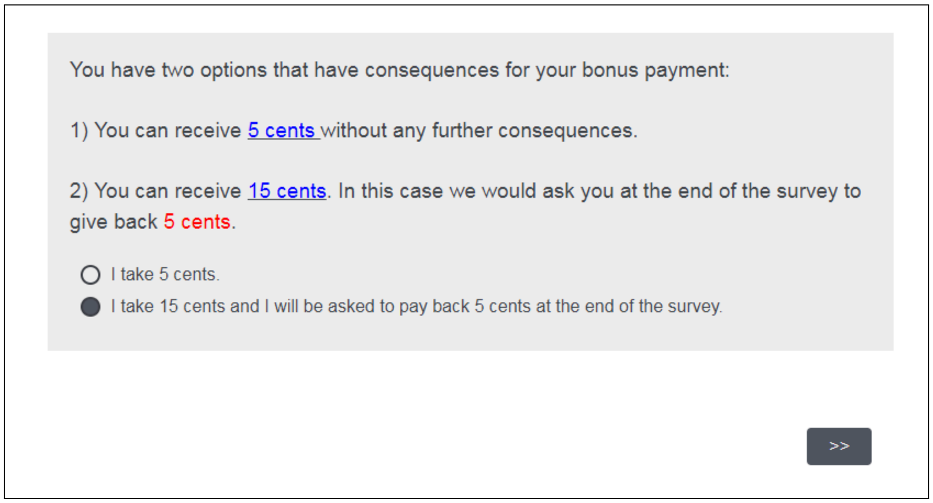

Figure S3. Start of Study 1a in the ask condition: Here the second option was chosen. The gray color change occurred with any focus on the radio box element.

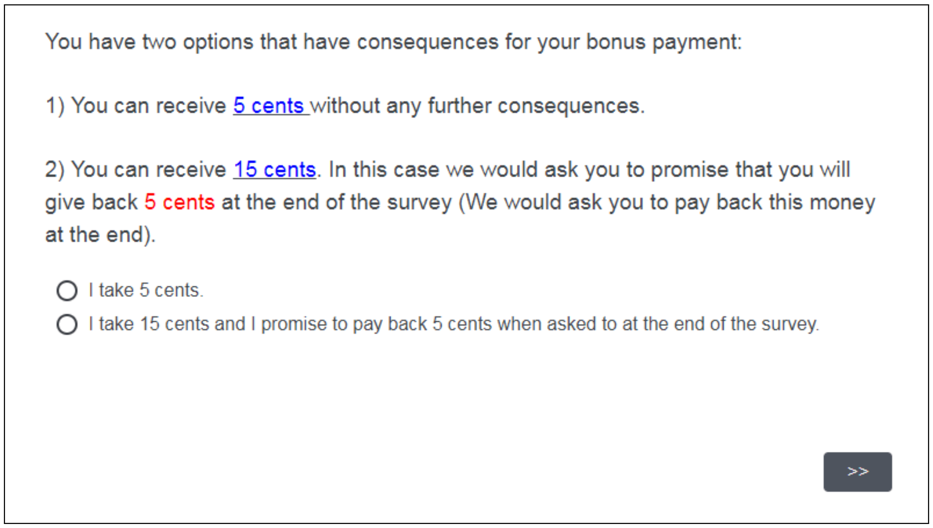

Figure S4. Start of Study 1a in the promise (click) condition

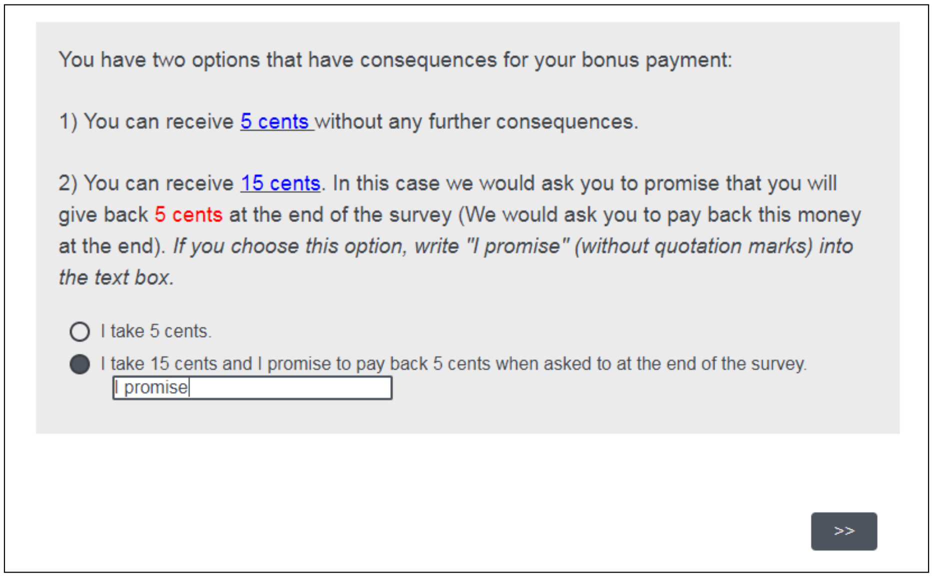

Figure S5. Start of Study 1a in the promise (write) condition: Here an option was chosen and the promise written into the text box (the gray color change occurred with any focus on the radio box element)

1.4 End of the survey

1.4.1 Control condition.

We gave you a choice at the beginning of the survey between taking 5 cents or 15 cents.

You decided to take 15 cents.

You can now decide whether you want to pay back 5 cents. We will not force you to pay any money back.

[*New page*]

How much money (in cents) do you pay back?

1.4.2 Ask condition.

We gave you a choice at the beginning of the survey between taking 5 cents or 15 cents.

You decided to take 15 cents.

We now ask you whether you want to pay back 5 cents. We will not force you to pay any money back.

[*New page*]

How much money (in cents) do you pay back?

1.4.3 Promise (click) and promise (write) condition. *See Figure S6 for a screen shot.*

We gave you a choice at the beginning of the survey between taking 5 cents or 15 cents.

You decided to take 15 cents and promised to pay back 5 cents.

We now ask you whether you want to pay back 5 cents. We will not force you to pay any money back.

How much money (in cents) do you pay back?

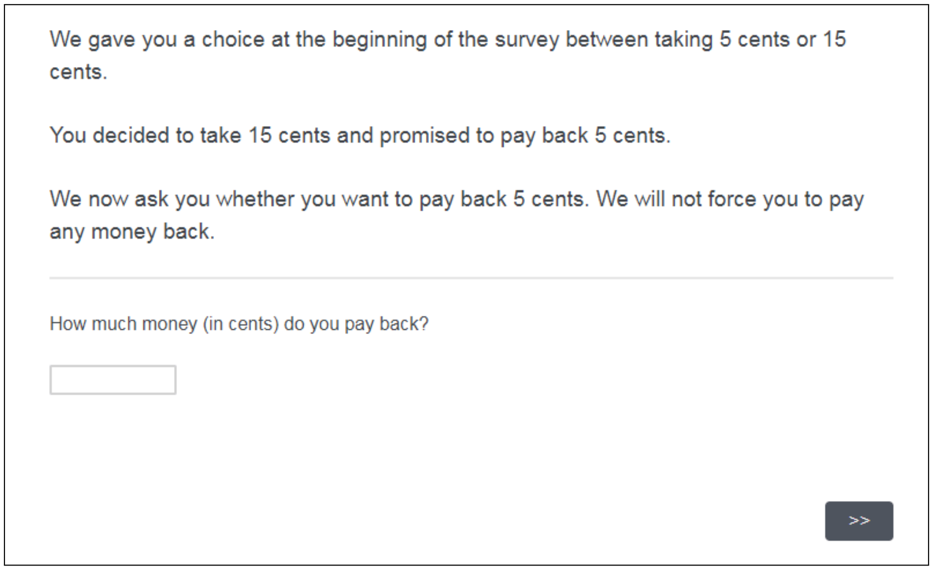

We gave you a choice at the beginning of the survey between taking 5 cents or 15 cents.

You decided to take 15 cents and promised to pay back 5 cents.

We now ask you whether you want to pay back 5 cents. We will not force you to pay any money back.

How much money (in cents) do you pay back?

>>

Figure S6. End of Study 1a in the promise conditions

1.5 Post-questionnaire

1.5.1 Non-promise conditions.

Please explain why you decided to choose the amount you did. [*Text box, 20 characters minimum*]

1.5.2 Promise conditions: promise broken.

See Figure S7 for a screen shot.

Please explain why you decided to break your promise and keep the money. [*Text box, 20 characters minimum*]

1.5.3 Promise conditions: promise kept.

Please explain why you decided to keep your promise and pay back the money. [*Text box, 20 characters minimum*]

1.5.4 All conditions: PANAS scale.

See Figure S8 for a screen shot.

How did you feel when making the decision to pay back or not to pay back the money?

- Distressed (-)
- Hostile (-)
- Proud (+)
- Scared (-)
- Irritable (-)
- Guilty (-)
- Excited (+)
- Upset (-)
- Interested (+)
- Afraid (-)
- Strong (+)
- Alert (+)
- Active (+)
- Jittery (-)
- Inspired (+)
- Nervous (-)

- Determined (+)
- Attentive (+)
- Ashamed (-)
- Enthusiastic (+)

[*All items were answered on a 5-point scale: (0) Very slightly or not at all, (1) A little, (2) Moderately, (3) Quite a bit, (4) Very much. Scores were separately added for positive and negative emotions to obtain two scale values.*]

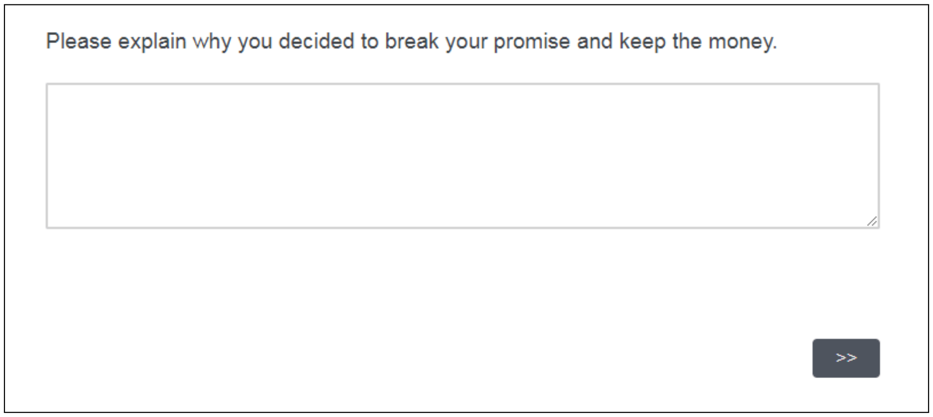

Figure S7. End of Study 1a after a broken promise: Open explanation of behavior

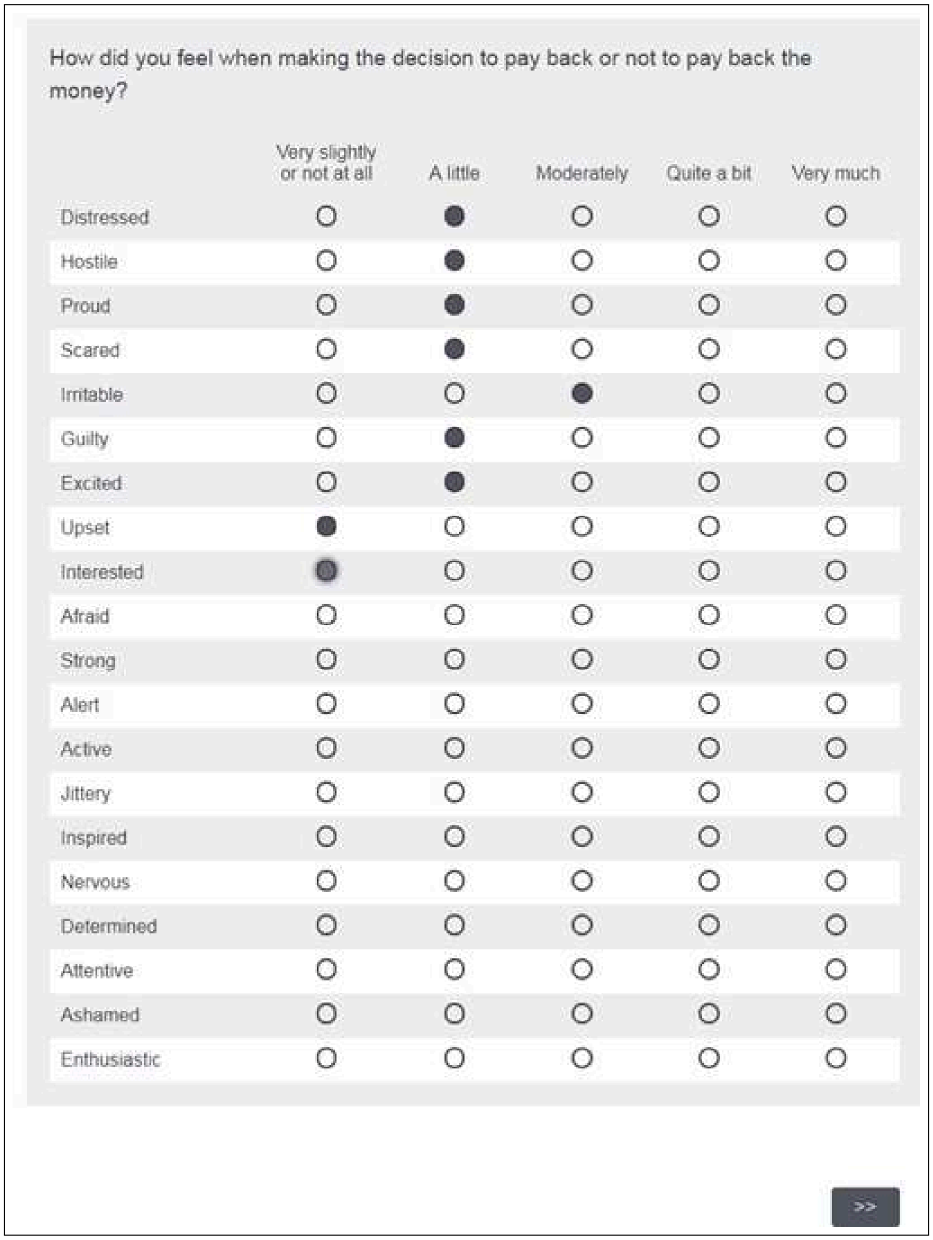

How did you feel when making the decision to pay back or not to pay back the money?

	Very slightly or not at all	A little	Moderately	Quite a bit	Very much
Distressed	○	●	○	○	○
Hostile	○	●	○	○	○
Proud	○	●	○	○	○
Scared	○	●	○	○	○
Irritable	○	○	●	○	○
Guilty	○	●	○	○	○
Excited	○	●	○	○	○
Upset	●	○	○	○	○
Interested	●	○	○	○	○
Afraid	○	○	○	○	○
Strong	○	○	○	○	○
Alert	○	○	○	○	○
Active	○	○	○	○	○
Jittery	○	○	○	○	○
Inspired	○	○	○	○	○
Nervous	○	○	○	○	○
Determined	○	○	○	○	○
Attentive	○	○	○	○	○
Ashamed	○	○	○	○	○
Enthusiastic	○	○	○	○	○

>>

Figure S8. End of Study 1a: PANAS scale partially filled in

2 Study 1b: Survey conditions and material

2.1 MTurk Specification

- **Announced timespan**: 12–22 minutes
- **Fixed compensation**: $1.00
- **Bonus payment**: on average $0.70 to $0.75
- **Time allotted**: 1 day
- **Auto-approval**: 3 days
- **Filters**: Hit approval rating $\geq$ 95%, Hits > 50, Location: US
- **Attention check:** yes

2.2 Attention checks

Participants had to pass one of two attention checks. The second check was only presented if the first test was failed. See Figure S9 for a screen shot.

> Choices of participants will always be affected by their preferences. Some contexts are less interesting than others. For example, we are not interested in answers that are given when not going through the texts. Therefore we need to make sure that you are actually taking the time. If you see this text then please disregard the sentence below. Instead, just move the last category (yellow) to the top position and leave everything else unchanged.
> Thank you very much.
>
> Please order the following **colors in order of your preference**: [*The entries in the list were draggable and could be moved up or down*]

1. white
2. green
3. orange
4. black
5. red
6. blue
7. violet
8. yellow

If a participant did not follow the instructions, a second test was shown, otherwise they continued to the task.

Thank you for sharing your color preferences with us. Unfortunately that was not question we wanted to you to answer. It seems that you have not seen the text above the?last question. We will give you a second chance to pass this test. Answer the following question by entering the word bookworm without any capitalized letter in the field below, nothing else.

What is your **favorite book** at the moment (including non-fiction)? [*Entry box*]

If a participant did not follow the instructions, he would be advised to return the HIT and were advised to return the HIT.

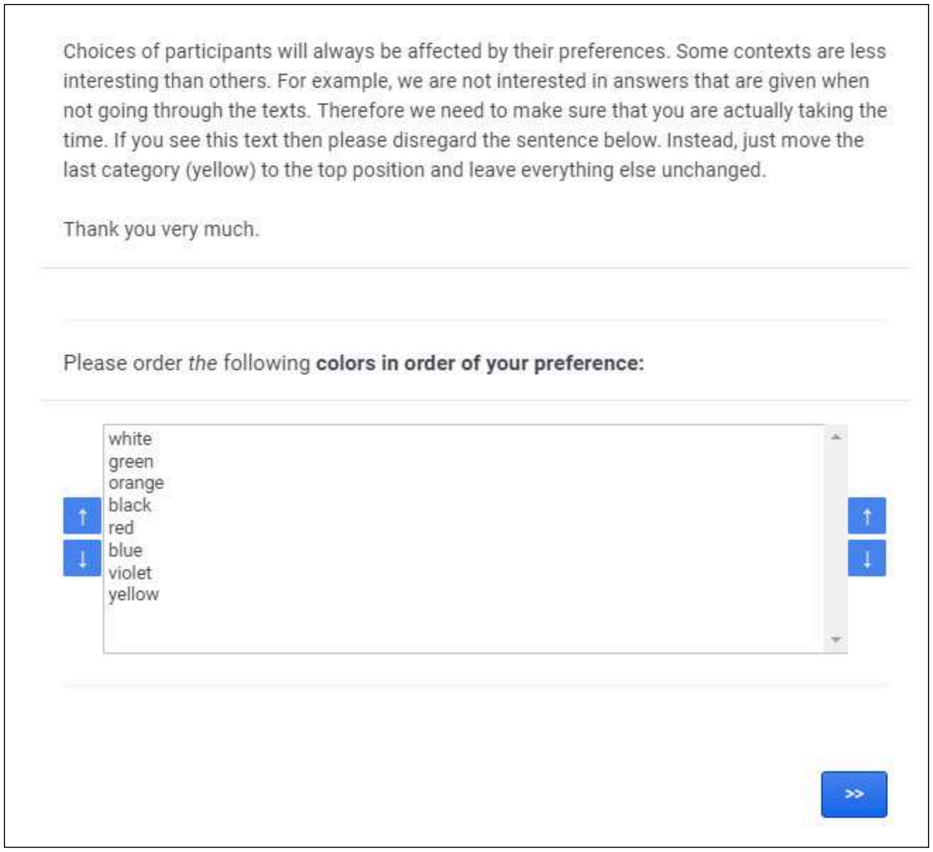

Figure S9. Qualtrics interface of first attention check in Study 1b

2.3 Estimation task

2.3.1 Both conditions. [*Note that there were two conditions in this survey: estimations for the promise (click) condition and the control condition from Study 1a. See Figure S10 and Figure S11 for screen shots.*]

> In this part, we will ask you to estimate how other participants decided in an earlier experiment.
>
> [*New page*]
>
> Participant in an experiment conducted in 2015 were given the choice between the two options shown below. You can receive an increase in bonus money if the estimates you give are close to the values we observed in 2015.
>
> [*Here, a conditions specific image of the choice screen for Study 1a was shown.*]
>
> Out of 100 participants, how many would you estimate took the second option (and received 15 cents)? [*text entry box*]
>
> [*New page*]
>
> At the end of the experiment in 2015, participants were given the chance to pay back money. They could pay back any amount (in cents) that they wanted.
>
> [*Here, a conditions specific image of the payback screen for Study 1a was shown.*]
>
> Out of 100 participants in 2015, how many do you estimate paid back at least 5 cents? [*text entry box*]
>
> How much money on average do you estimate was paid back by participants in 2015 [in cents][1]? [*text entry box*]

[1] This question was dropped from analyses for the following reason: Several respondents responded with values larger than 5 cents, as the sequence of question might have led them to estimate the average for participants who paid back at least 5 cents.

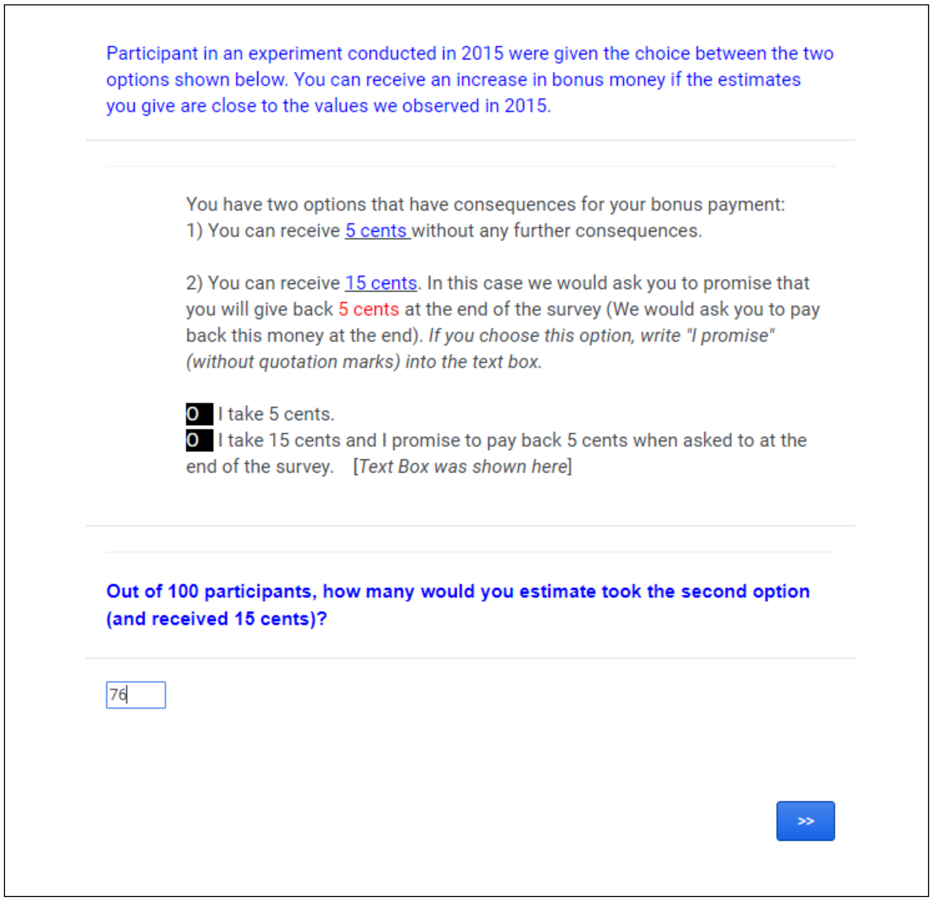

Participant in an experiment conducted in 2015 were given the choice between the two options shown below. You can receive an increase in bonus money if the estimates you give are close to the values we observed in 2015.

You have two options that have consequences for your bonus payment:
1) You can receive 5 cents without any further consequences.

2) You can receive 15 cents. In this case we would ask you to promise that you will give back 5 cents at the end of the survey (We would ask you to pay back this money at the end). *If you choose this option, write "I promise" (without quotation marks) into the text box.*

O I take 5 cents.
O I take 15 cents and I promise to pay back 5 cents when asked to at the end of the survey. [*Text Box was shown here*]

Out of 100 participants, how many would you estimate took the second option (and received 15 cents)?

76

>>

Figure S10. Study 1b: Estimation Interface (Promise condition) Part 1

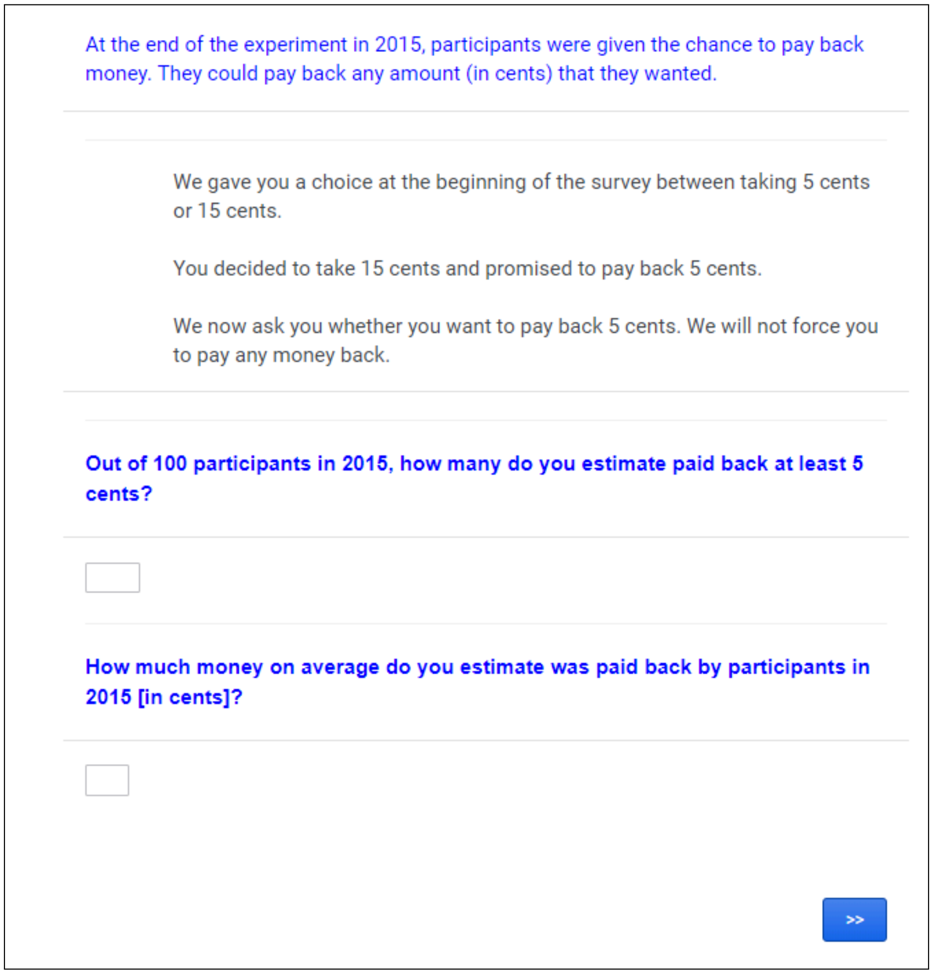

At the end of the experiment in 2015, participants were given the chance to pay back money. They could pay back any amount (in cents) that they wanted.

We gave you a choice at the beginning of the survey between taking 5 cents or 15 cents.

You decided to take 15 cents and promised to pay back 5 cents.

We now ask you whether you want to pay back 5 cents. We will not force you to pay any money back.

Out of 100 participants in 2015, how many do you estimate paid back at least 5 cents?

How much money on average do you estimate was paid back by participants in 2015 [in cents]?

>>

Figure S11. Study 1b: Estimation Interface (Promise condition) Part 2
