# Supplementary material for: Most People Keep Their Word Rather Than Their Money
Source: Open Mind (Camb). 2019 Jul 1;3:68–88. doi: 10.1162/opmi_a_00027 (PMC8412196; doi:10.1162/opmi_a_00027)
Supplement: Supplementary file 2 [file opmi-03-68-s002.pdf]

Supplementary Material (2/4) for "Most People Keep Their Word Rather Than Their Money"

Study 2a, 2b

Jan K. Woike
Max-Planck-Institute for Human Development, Berlin

Patricia Kanngiesser
Freie Universität Berlin

Compiled on May 2nd, 2019

Contents

1 Study 2a: Survey conditions and material 3
1.1 MTurk Specification . . . 3
1.2 Start of the survey . . . 3
1.2.1 Control condition **20-10** . . . 3
1.2.2 Control condition **200-100** . . . 3
1.2.3 Promise (write) condition **20-10** . . . 4
1.2.4 Promise (write) condition **200-100** . . . 4
1.2.5 Higher bonus rejected (all conditions) . . . 4
1.3 End of the survey . . . 7
1.3.1 Control condition **20-10** . . . 7
1.3.2 Control condition **200-100** . . . 7
1.3.3 Promise (write) condition **20-10** . . . 7
1.3.4 Promise (write) condition **200-100** . . . 7
1.4 Post-questionnaire . . . 8
1.4.1 Promise: kept promise . . . 8
1.4.2 Promise: broken promise . . . 8
1.4.3 Control: money paid back . . . 8
1.4.4 Control: money not paid back . . . 9
1.4.5 All conditions: PANAS scale . . . 9
1.5 Comprehension check . . . 10

2 Study 2b: Survey conditions and material 12
2.1 MTurk Specification . . . 12
2.2 Instructions: Quadratic Scoring Rule . . . 12
2.3 Estimation Task . . . 18

List of Figures

S12 Start of Study 2a: Control **20-10** . . . 5
S13 Start of Study 2a: Promise **200-100** . . . 5
S14 Study 2a: Choice explanations . . . 6
S15 Study 2a: Comprehension check . . . 11
S16 Study 2b: QSR Instructions Page 1 . . . 14
S17 Study 2b: QSR Instructions Page 2 . . . 15
S18 Study 2b: QSR Instructions Page 3 . . . 16
S19 Study 2b: QSR Instructions Page 4 . . . 17
S20 Study 2b: Estimation Task 1 . . . 19
S21 Study 2b: Estimation Task 2 . . . 19
S22 Study 2b: Estimation sliders . . . 20
S23 Study 2b: Estimation sliders example . . . 21

1 Study 2a: Survey conditions and material

1.1 MTurk Specification

- **Announced timespan**: 8–12 minutes
- **Fixed compensation**: $1.00
- **Bonus payment**: possibility of bonus payment announced
- **Time allotted**: 1 hour
- **Auto-approval**: 36 hours
- **Filters**: Hit approval rating $\geq$ 95%, Location: US
- **Attention check:** no

1.2 Start of the survey

1.2.1 Control condition 20-10. *See Figure S12 for a screenshot.*

You have two options that have consequences for your bonus payment:

1. You can receive 5 cents without any further consequences.
2. You can receive 20 cents. In this case we would give you a chance to give back 10 cents at the end of the survey. (You will be able to determine how much you want to pay back).

[*Participants were offered two options in check boxes:*]

- I take 5 cents.
- I take 20 cents and I can decide at the end of the survey whether I want to pay back 10 cents.

1.2.2 Control condition 200-100.

You have two options that have consequences for your bonus payment:

1. You can receive 50 cents without any further consequences.
2. You can receive 200 cents. In this case we would give you a chance to give back 100 cents at the end of the survey. (You will be able to determine how much you want to pay back).

[*Participants were offered two options in check boxes:*]

- I take 50 cents.
- I take 200 cents and I can decide at the end of the survey whether I want to pay back 100 cents.

1.2.3 Promise (write) condition 20-10.

You have two options that have consequences for your bonus payment:

1. You can receive 5 cents without any further consequences.
2. You can receive 20 cents. In this case we would ask you to promise that you will give back 10 cents at the end of the survey. (We would ask you to pay back this money at the end). *If you choose this option, write "I promise" (without quotation marks) into the text box.*

[*Participants were offered two options in check boxes:*]

- I take 5 cents.
- I take 20 cents and I promise to pay back 10 cents when asked to at the end of the survey. [*Text entry box*]

1.2.4 Promise (write) condition 200-100.

See Figure S13 for a screen shot.

You have two options that have consequences for your bonus payment:

1. You can receive 50 cents without any further consequences.
2. You can receive 200 cents. In this case we would ask you to promise that you will give back 100 cents at the end of the survey. (We would ask you to pay back this money at the end). *If you choose this option, write "I promise" (without quotation marks) into the text box.*

[*Participants were offered two options in check boxes:*]

- I take 50 cents.
- I take 200 cents and I promise to pay back 100 cents when asked to at the end of the survey. [*Text entry box*]

1.2.5 Higher bonus rejected (all conditions). [*For participants who declined the higher payment, the following list of questions was presented in a decision matrix. All items were answered on a 5-point scale: Strongly disagree-Somewhat disagree-Neither agree nor disagree-Somewhat agree-Strongly agree. The order of questions was randomized for each participant. See Figure S14 for a screen shot.*]

How much do you agree with the following statements?

- I did not want to make a promise.
- I chose the simpler option.
- I was concerned about negative consequences of my decision.
- I chose the certain option.

[*Participants who reject the higher payment did not encounter the end of survey block*]

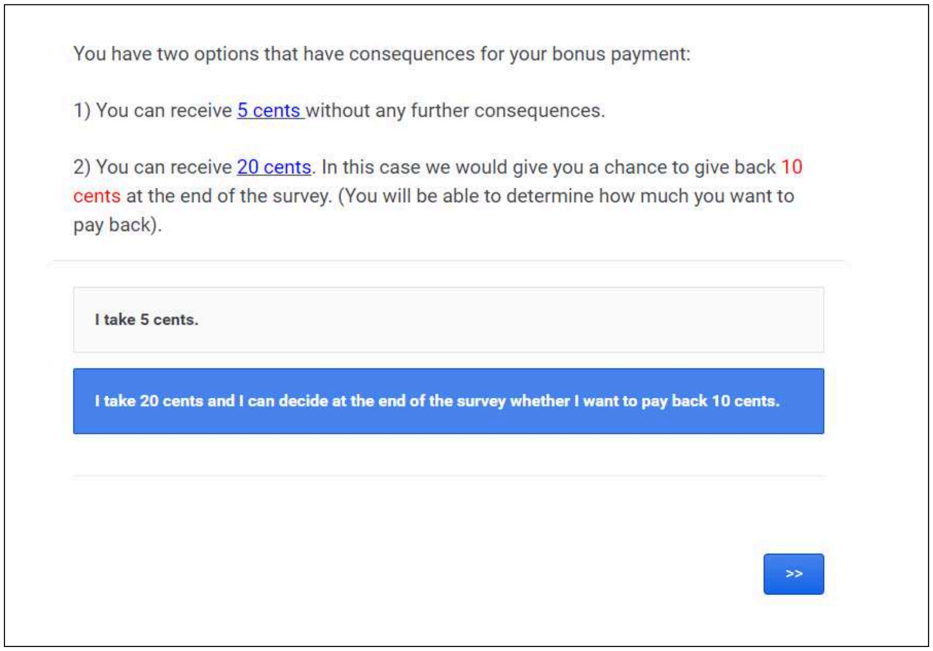

Figure S12. Start of Study 2a in the control **20-10** condition

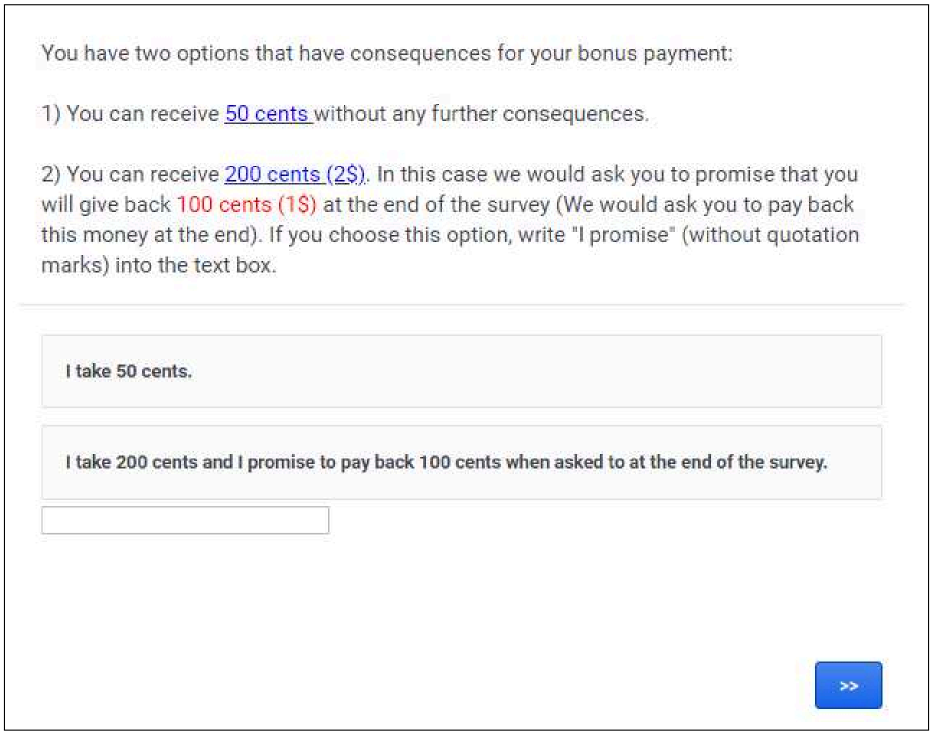

Figure S13. Start of Study 2a in the promise **200-100** condition

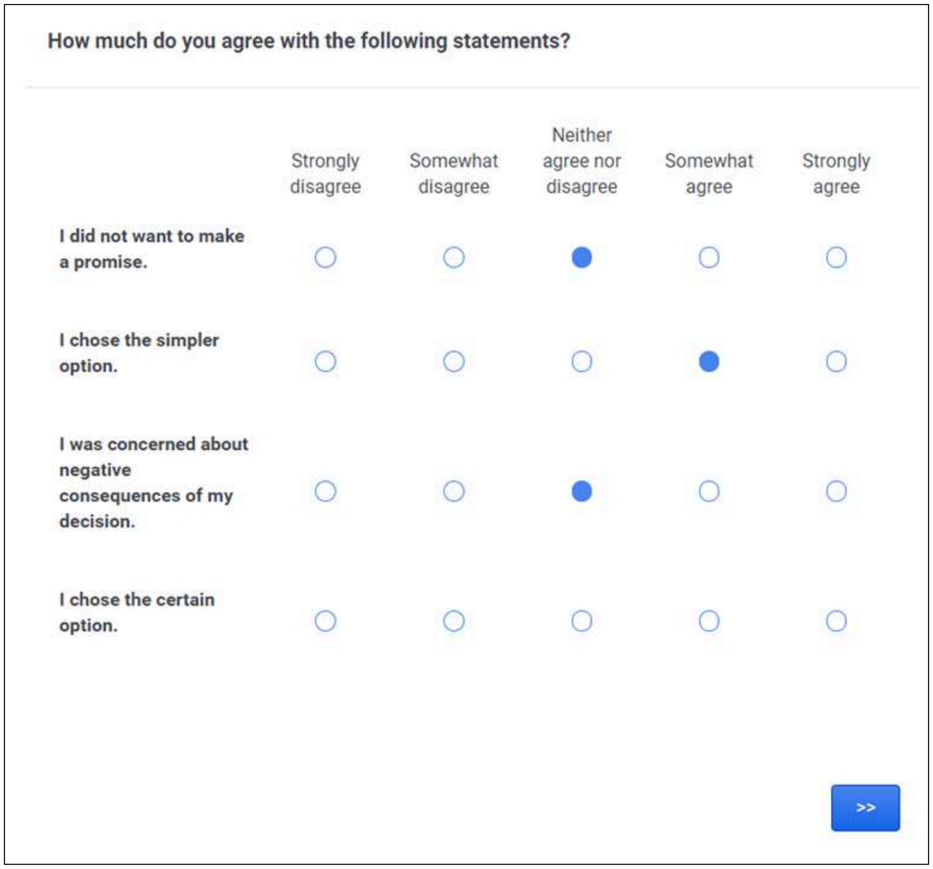

Figure S14. Choice explanations in Study 2a after rejecting the higher payment

1.3 End of the survey

1.3.1 Control condition 20-10.

We gave you a choice at the beginning of the survey between taking 5 cents or 20 cents.

You decided to take 20 cents.

You can now decide whether you want to pay back 10 cents.?We will not force you to pay any money back.

How many cents do you pay back? (Please enter an amount between 0 and 20)

1.3.2 Control condition 200-100.

We gave you a choice at the beginning of the survey between taking 50 cents or 200 cents.

You decided to take 200 cents.

You can now decide whether you want to pay back 100 cents.?We will not force you to pay any money back.

How many cents do you pay back? (Please enter an amount between 0 and 200)

1.3.3 Promise (write) condition 20-10.

We gave you a choice at the beginning of the survey between taking 5 cents or 20 cents.

You decided to take 20 cents and promised to pay back 10 cents.

We now ask you whether you want to pay back 10 cents.?We will not force you to pay any money back.

How many cents do you pay back? (Please enter an amount between 0 and 20)

1.3.4 Promise (write) condition 200-100.

We gave you a choice at the beginning of the survey between taking 50 cents or 200 cents.

You decided to take 200 cents and promised to pay back 100 cents.

We now ask you whether you want to pay back 100 cents.?We will not force you to pay any money back.

How many cents do you pay back? (Please enter an amount between 0 and 200)

1.4 Post-questionnaire

1.4.1 Promise: kept promise. [*For participants in the promise conditions who paid back at least the promised amount, the following list of questions was presented in a decision matrix. All items were answered on a 5-point scale: Strongly disagree-Somewhat disagree-Neither agree nor disagree-Somewhat agree-Strongly agree. The order of questions was randomized for each participant.*]

How much do you agree with the following statements?

- I paid back the money because one should keep promises.
- I paid back the money because I am a person who always keeps promises given to another.
- I paid back the money because I wanted to do the right thing.
- I paid back the money because the amount was small.

1.4.2 Promise: broken promise. [*For participants in the promise conditions who paid back less than the promised amount, the following list of questions was presented in a decision matrix. All items were answered on a 5-point scale: Strongly disagree-Somewhat disagree-Neither agree nor disagree-Somewhat agree-Strongly agree. The order of questions was randomized for each participant.*]

How much do you agree with the following statements?

- I did not want to make a promise.
- I chose the simpler option.
- I was concerned about negative consequences of my decision.
- I chose the certain option.

1.4.3 Control: money paid back. [*For participants in the control conditions who paid back at least the reference amount, the following list of questions was presented in a decision matrix. All items were answered on a 5-point scale: Strongly disagree-Somewhat disagree-Neither agree nor disagree-Somewhat agree-Strongly agree. The order of questions was randomized for each participant.*]

How much do you agree with the following statements?

- I paid back some money, because I wanted to be nice to the requester.
- I paid back some money, because I expect to earn more money as a consequence.
- I paid back some money, because I am a generous person.

1.4.4 Control: money not paid back.

[*For participants in the control conditions who paid back less than the reference amount, the following list of questions was presented in a decision matrix. All items were answered on a 5-point scale: Strongly disagree-Somewhat disagree-Neither agree nor disagree-Somewhat agree-Strongly agree. The order of questions was randomized for each participant.*]

How much do you agree with the following statements?

- I did not pay back money, because there was no reason to do so.
- I did not pay back money, because I worked hard for it.

1.4.5 All conditions: PANAS scale.

How did you feel when making the decision to pay back or not to pay back money?

- Distressed (-)
- Hostile (-)
- Proud (+)
- Scared (-)
- Irritable (-)
- Guilty (-)
- Excited (+)
- Upset (-)
- Interested (+)
- Afraid (-)
- Strong (+)
- Alert (+)
- Active (+)
- Jittery (-)
- Inspired (+)
- Nervous (-)
- Determined (+)
- Attentive (+)
- Ashamed (-)

- Enthusiastic (+)

[*All items were answered on a 5-point scale: (0) Very slightly or not at all, (1) A little, (2) Moderately, (3) Quite a bit, (4) Very much. Scores were separately added for positive and negative emotions to obtain two scale values.*]

1.5 Comprehension check

See Figure S15 for a screen shot.

The money amount I chose in this part was...[*answer options were presented as radio boxes*]

- given to another participant
- taken from another participant
- paid back to the requester [*always correct response*]
- imaginary money

In the beginning of this task... [*answer options were presented as radio boxes*]

- I made a promise [*correct response in promise conditions*]
- I did not make a promise [*correct response in control conditions*]

How much bonus money have you earned in this part (in cents)? (*The answer is the amount of money you were given in the beginning minus the amount you have just paid back in the end. The bonus money does not include the $1.00 fixed payment for the HIT.*). [*text entry box*]

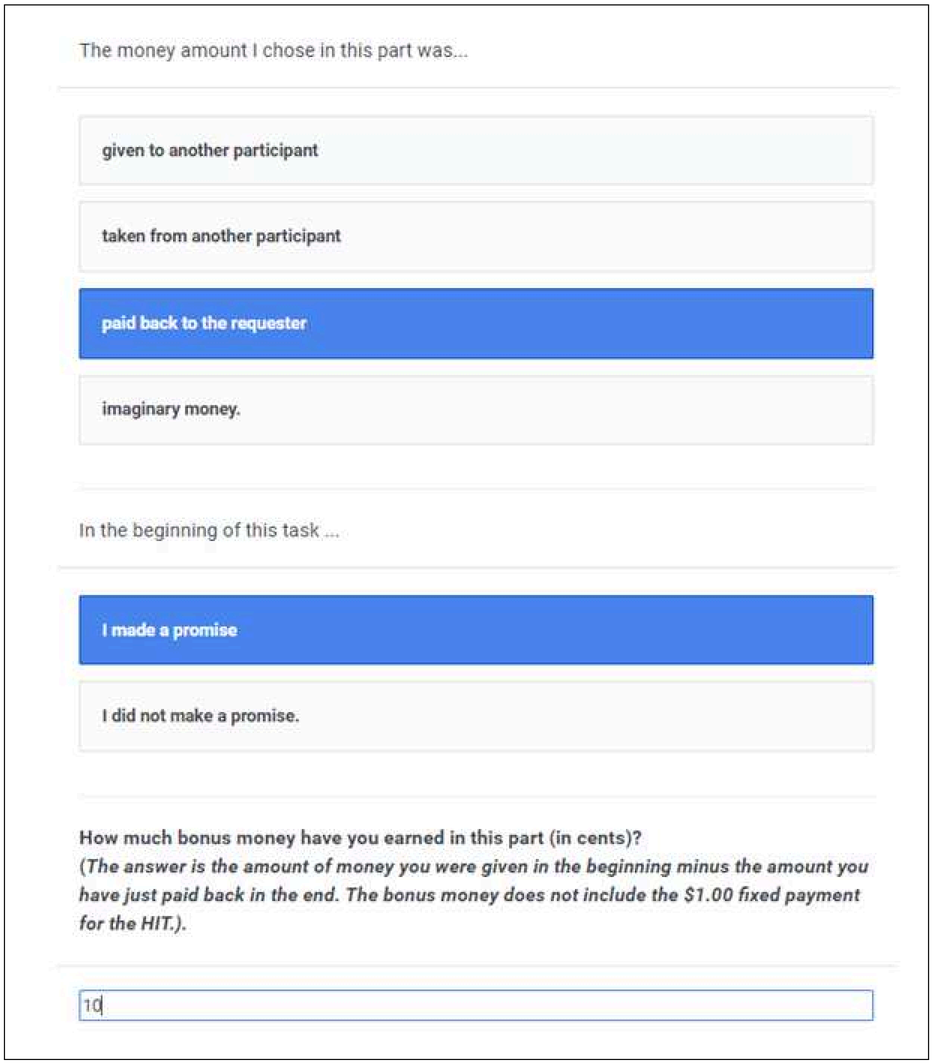

The money amount I chose in this part was...

given to another participant

taken from another participant

paid back to the requester

imaginary money.

In the beginning of this task ...

I made a promise

I did not make a promise.

How much bonus money have you earned in this part (in cents)?
(The answer is the amount of money you were given in the beginning minus the amount you have just paid back in the end. The bonus money does not include the $1.00 fixed payment for the HIT.).

10

Figure S15. Comprehension check at the end of Study 2a with answers entered

2 Study 2b: Survey conditions and material

2.1 MTurk Specification

- **Announced timespan**: 10–20 minutes
- **Fixed compensation**: $1.20
- **Bonus payment**: Size of bonus payment depends on decisions
- **Time allotted**: 90 minutes
- **Auto-approval**: 3 days
- **Filters**: Hit approval rating > 97%, Hits > 100, Location: US
- **Attention check:** no

2.2 Instructions: Quadratic Scoring Rule

See Figure S16 for a screen shot.

In this task, we will ask you to estimate which percentage of participants reacted in certain ways. We will not ask you for a single number, as we would like to see the range of values you judge plausible.

We will use a format as seen below (with more intervals). You can assign 100 points across the rows. You should assign more points in rows corresponding to intervals that you think are more plausible. You can play around with the abstract example below.

Assign points by clicking on one of the rows. If you have already assigned 100 points, you need to reduce the number of points in other rows (again by clicking on the rows, left of the previous click).

Continue if you think you feel comfortable with the format.

You need to have assigned 100 points.

[*Participants interacted with 5 sliders labeled "Interval 1" to "Interval 5" shown below the text that could represent values between 0 and 100 (with a reference scale and an explicit number stating the current value). A maximum allocation of 100 points across all sliders was enforced by the element, a minimum amount of 100 by the validation logic.*]

[*New page: See Figure S17 for a screen shot.*]

For this task, we will give you 50 cents of additional bonus money.

Depending on your answers, you can gain or lose money. Specifically, you can gain up to 50 cents more, or lose up to the 50 cents we just gave you (so that in the worst case your bonus would be 0 cents for this part).

How much you gain or lose depends on your distribution of points (in relation to the true correct answer). This slightly complex procedure is chosen to encourage you to distribute points according to what you believe (you cannot expect to gain from gambling, here).

Imagine that you put all points into one interval.

You would get the following returns if the true value is in one of the ten intervals:

[*Ten non-interactive sliders were shown with one slider set to 100, the other sliders to 0. On the left side of the slider, the monetary amounts received were shown in case the true value corresponded to the respective slider.*]

You would receive 50 cents if the true value was in the chosen interval, but you would lose 50 cents if not, no matter how far off your estimate was from the true value.

[*New page: See Figure S18 for a screen shot.*]

If you chose to distribute points into two intervals, then you would receive 25 cents if the true value was in either interval, but you would lose 25 cents otherwise.

[*Ten non-interactive sliders were shown with two sliders set to 50, the other sliders to 0. Monetary results were shown on the left side.*]
[*New page: See Figure S19 for a screen shot.*]

If you distribute points across five intervals as shown below, you would receive 27 for the category with 40 points, 7 points for the intervals with 20 points, and lose 3 cents for the intervals with 10 points. You would lose 13 points for the intervals without points.

So you could win up to 27 cents or lose up to 13 cents depending on the true value.

[*Ten non-interactive sliders were shown with five sliders in order set to 10,20, 40, 20, and 10. The other sliders were set to 0. Monetary results were shown on the left side.*]

As a final example: If you distribute points across more evenly and across more intervals, you could lose up to 1 cent and gain up to 10 cents, as shown below.

[*Ten non-interactive sliders were shown that were in order set to 5,10,10,10, 15, 15, 10, 10, 10, and 5. Monetary results were shown on the left side, in order: -1 cent, 5 cents, 5 cents, 5 cents, 10 cents, 10 cents, 5 cents, 5 cents, 5 cents, -1 cents.*]

We will now explain the question that we want you to answer in the format discussed above.

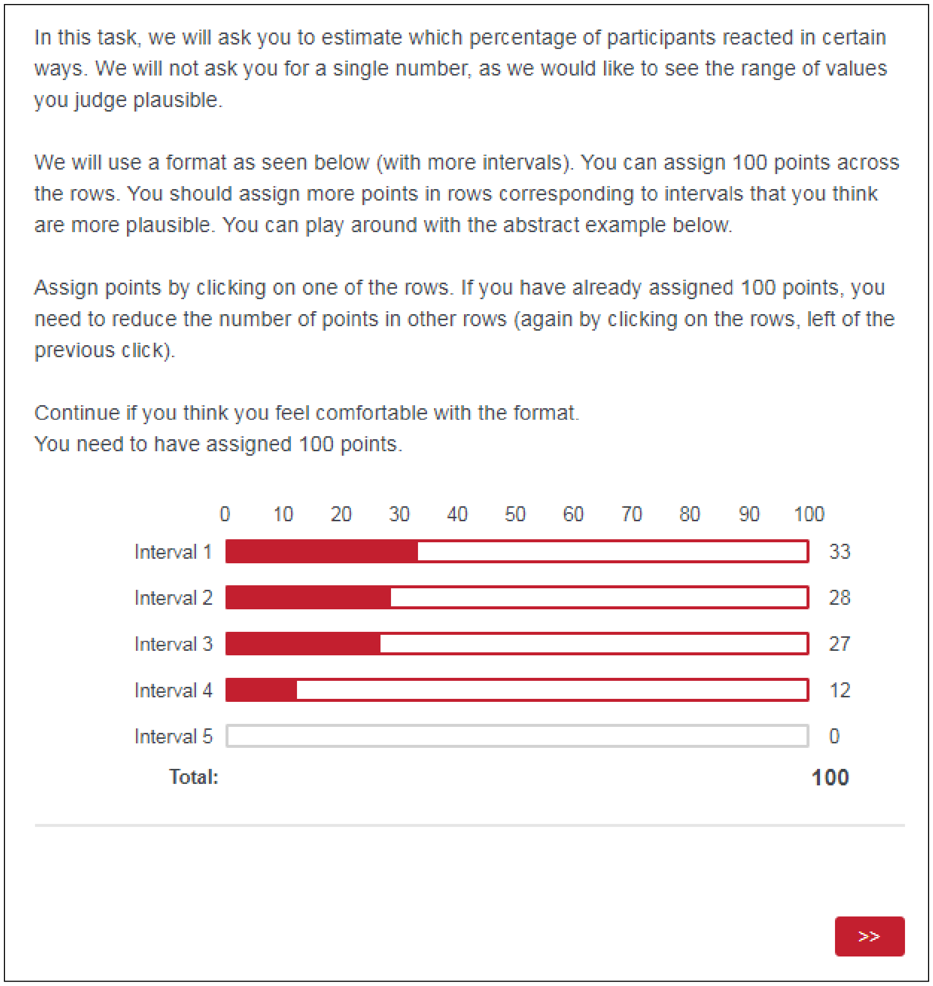

In this task, we will ask you to estimate which percentage of participants reacted in certain ways. We will not ask you for a single number, as we would like to see the range of values you judge plausible.

We will use a format as seen below (with more intervals). You can assign 100 points across the rows. You should assign more points in rows corresponding to intervals that you think are more plausible. You can play around with the abstract example below.

Assign points by clicking on one of the rows. If you have already assigned 100 points, you need to reduce the number of points in other rows (again by clicking on the rows, left of the previous click).

Continue if you think you feel comfortable with the format.
You need to have assigned 100 points.

Figure S16. Instructions for quadratic scoring rule in Study 2b (page 1) with points assigned

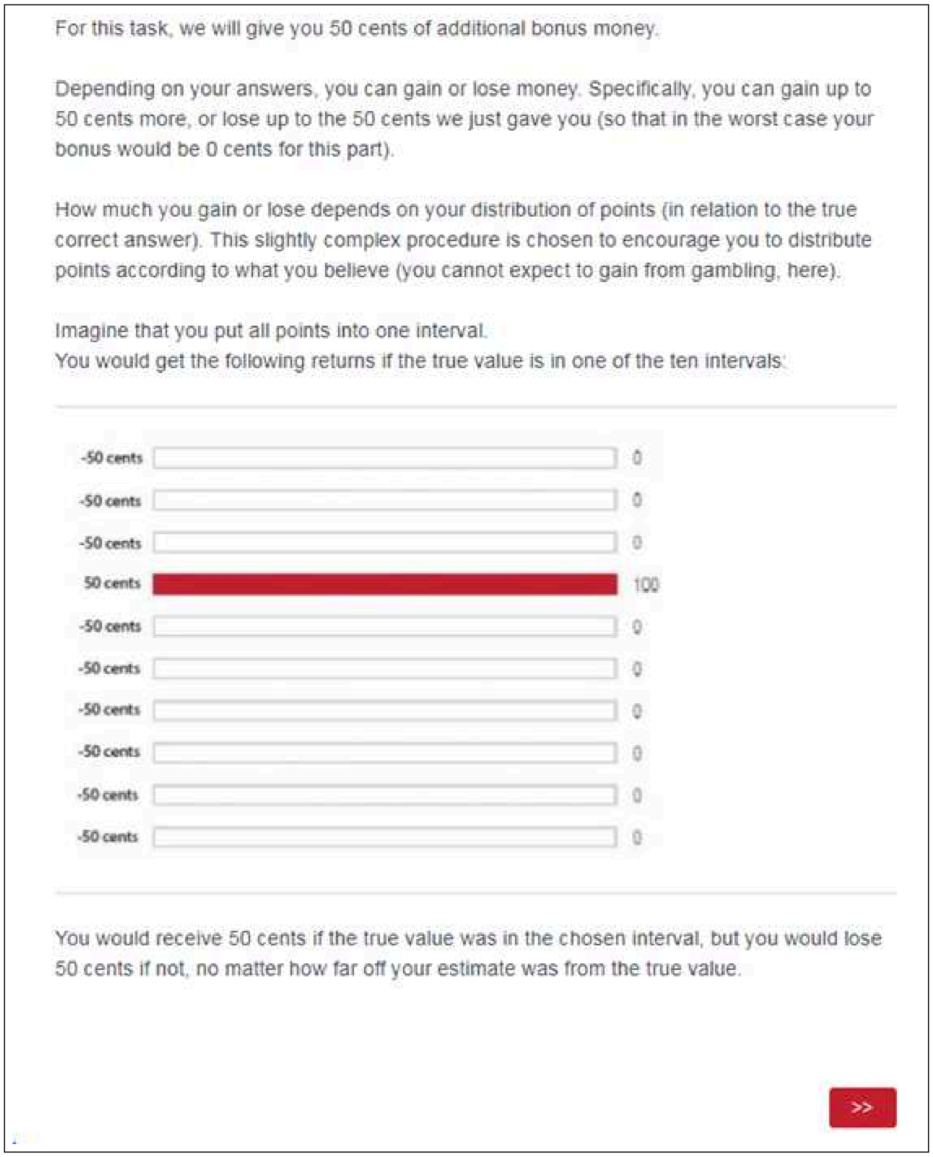

Figure S17. Instructions for quadratic scoring rule in Study 2b (page 2)

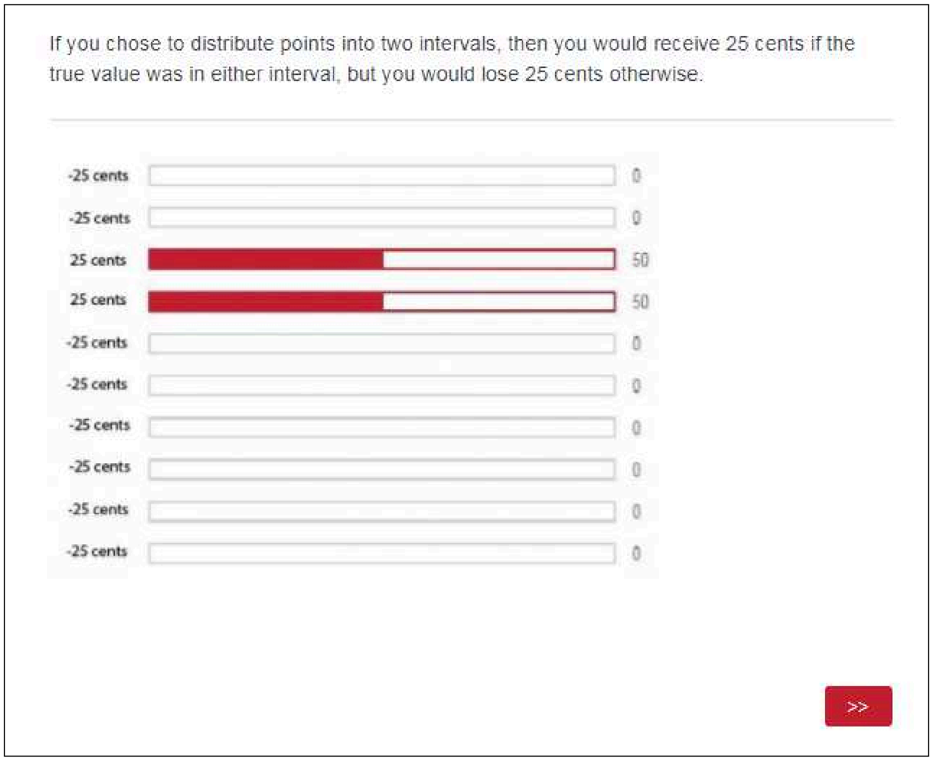

Figure S18. Instructions for quadratic scoring rule in Study 2b (page 3)

If you distribute points across five intervals as shown below, you would receive 27 for the category with 40 points, 7 points for the intervals with 20 points, and lose 3 cents for the intervals with 10 points. You would lose 13 points for the intervals without points.

So you could win up to 27 cents or lose up to 13 cents depending on the true value.

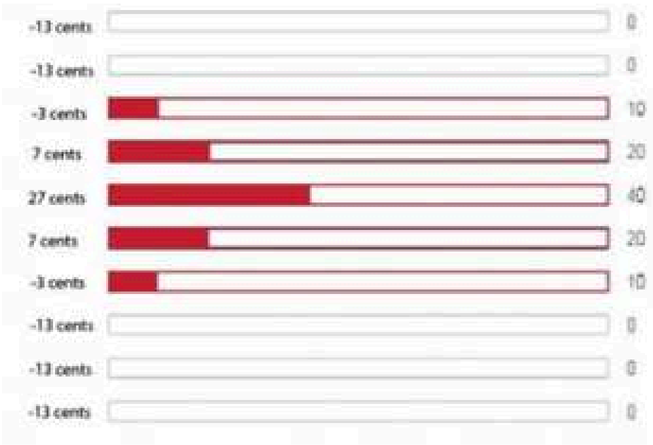

As a final example: If you distribute points across more evenly and across more intervals, you could lose up to 1 cent and gain up to 10 cents, as shown below.

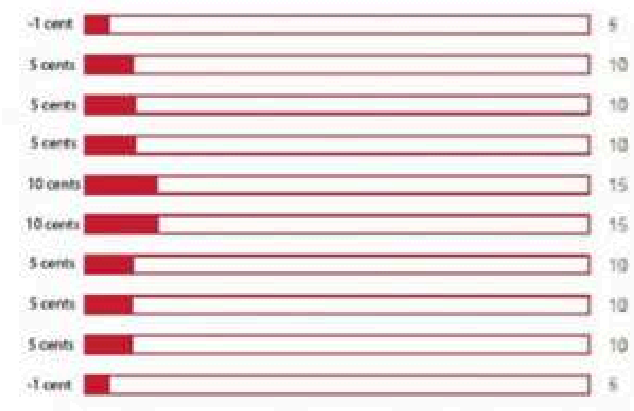

We will now explain the question that we want you to answer in the format discussed above.

Figure S19. Instructions for quadratic scoring rule in Study 2b (page 4)

2.3 Estimation Task

[*This task was presented in four conditions: (Control **20-10**, Control **200-100**, Promise **20-10**, and Promise **200-100**. The conditions varied regarding the screen shots taken from interfaces in Study 2a. Figure S20 shows an example for the first page.*]

Participants in an earlier survey were given two options at the start of the survey:

[*A condition specific image of the first choice in Study 2a was shown.*]

All participants who chose the second option, were asked at the end of the survey how much money they wanted to pay back.

[*A condition specific image of the final decision in Study 2a was shown. Figure S21 shows an example for the second page.*]

[*New page*]

> On the next page, we would like you to estimate, which percentage of participants who had chosen the second option paid back [**10/100**] or more cents at the end of the survey. [*The amount corresponded to the condition: 10 for Control **20-10** and Promise **20-10**, 100 for Control **200-100** and Promise **200-100**.*]

[*New page: See Figure S22 for a screen shot of the interface before inputs and Figure S23 for a screen shot of the interface after an exemplary input.*]

> Please distribute 100 points according to which intervals (shown in rows) you think are likely to contain the true percentage of participants.

[*Participants interacted with 22 sliders labeled with percentage intervals. The intervals started with [0%-0.01%], [>0.01%-5%], [5%%-10%], then in 5% intervals up to [>90%-95%]and finally [>95%-99.99%] and [>99.99%-100%]. The value between 0 and 100 for each slider was shown on the right of the slider. A maximum total allocation of 100 points across all sliders was enforced by the element, a minimum amount of 100 by the validation logic.*]

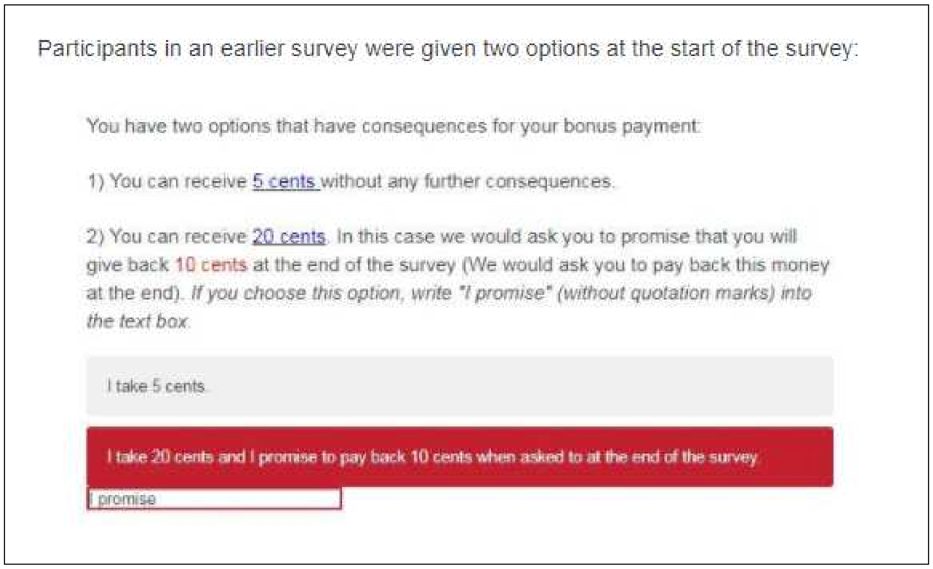

Figure S20. First image for the promise **20-10** condition in the estimation part of Study 2b

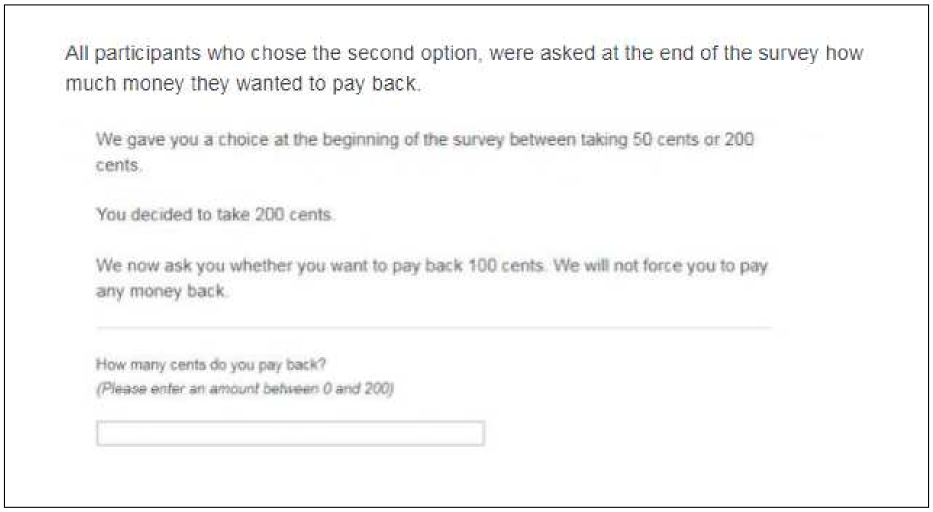

Figure S21. Second image for the control **200-101** condition in the estimation part of Study 2b

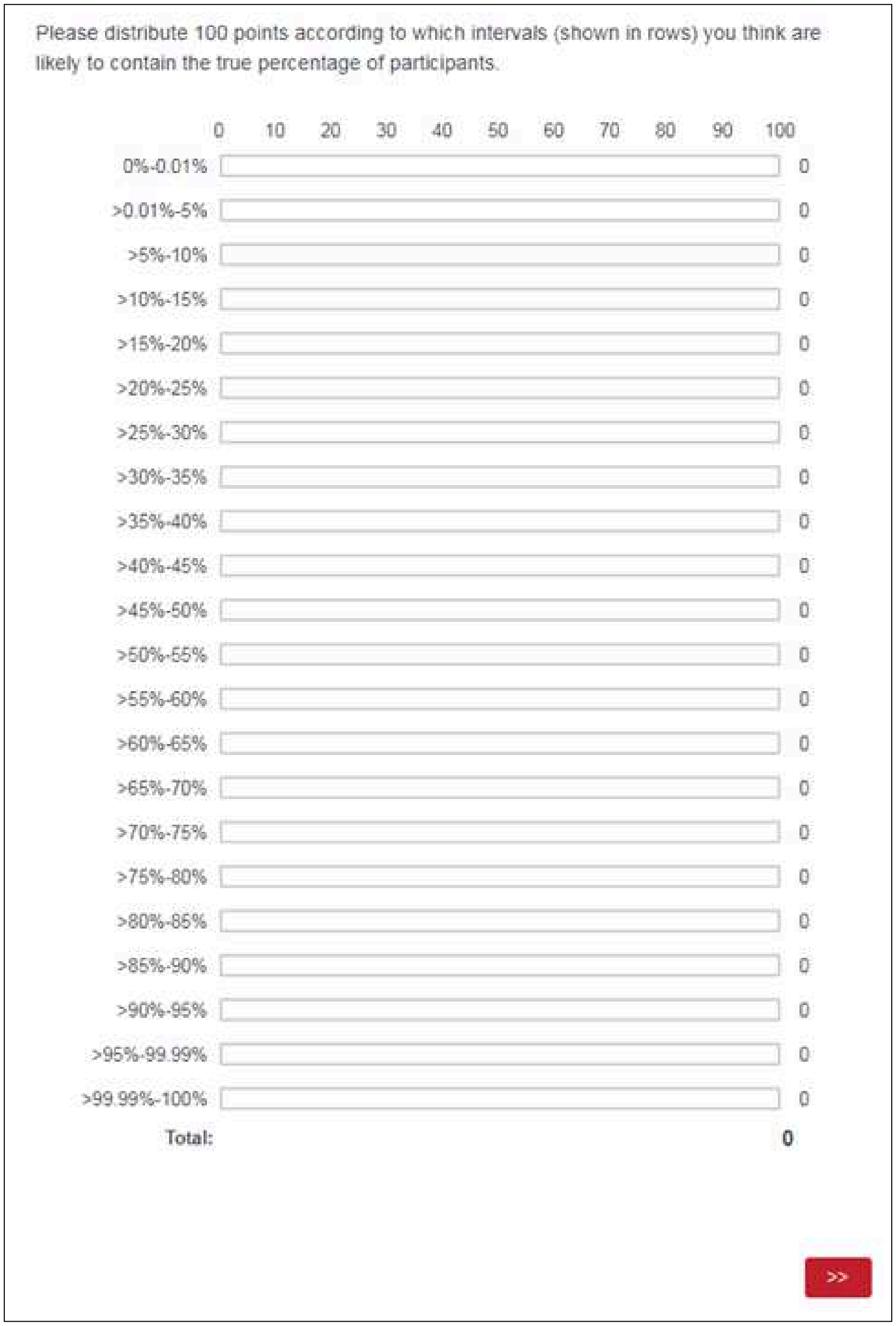

Figure S22. Estimation sliders for Study 2b, default positions

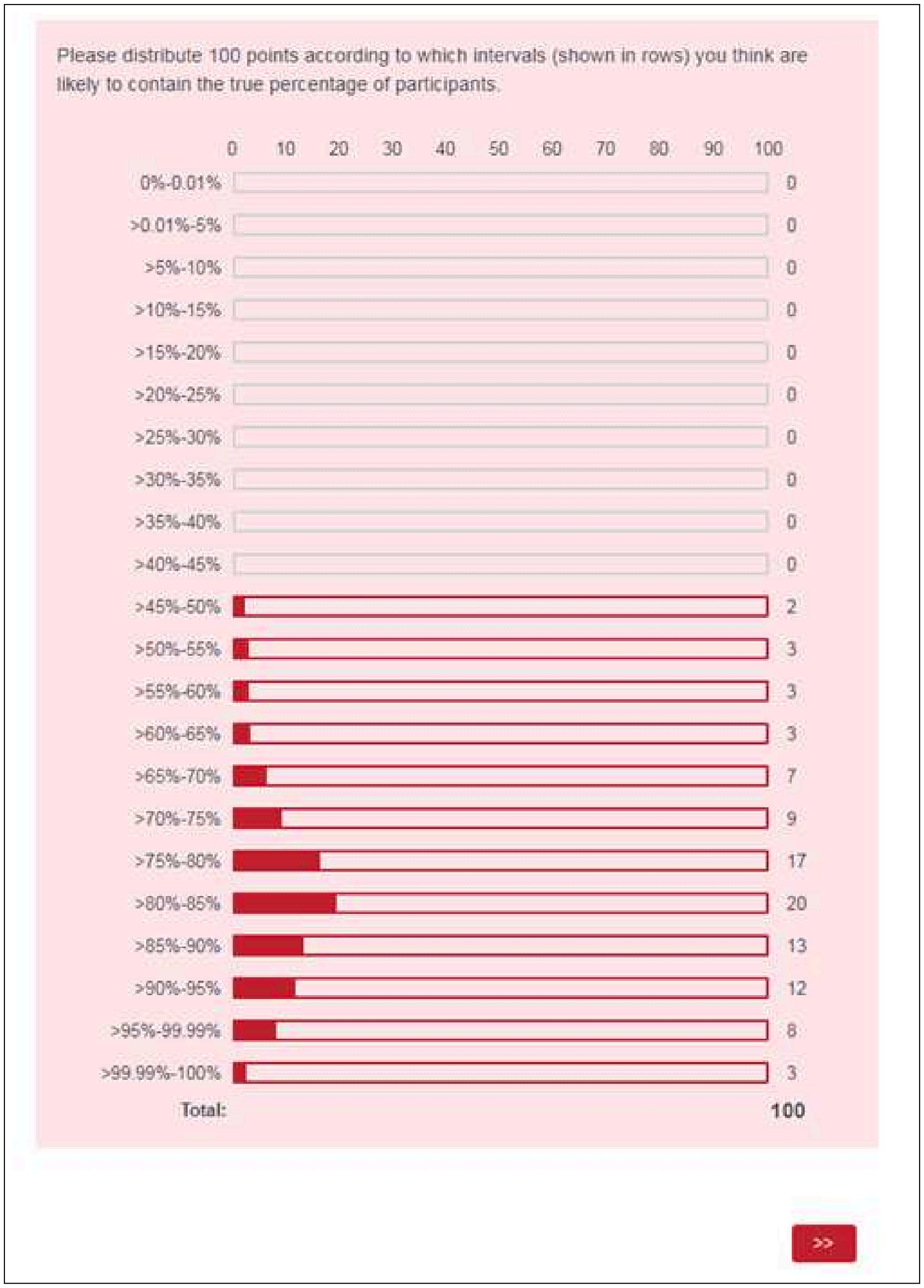

Figure S23. Estimation sliders for Study 2b, example of distribution
