# Supplementary material for: Most People Keep Their Word Rather Than Their Money
Source: Open Mind (Camb). 2019 Jul 1;3:68–88. doi: 10.1162/opmi_a_00027 (PMC8412196; doi:10.1162/opmi_a_00027)
Supplement: Supplementary file 3 [file opmi-03-68-s003.pdf]

Supplementary Material (3/4) for "Most People Keep Their Word Rather Than Their Money"

Study 3

Jan K. Woike
Max-Planck-Institute for Human Development, Berlin
Patricia Kanngiesser
Freie Universität Berlin

Compiled on May 2nd, 2019

Contents

1 Study 3: Survey conditions and material 3
1.1 MTurk Specification . . . 3
1.2 Attention checks . . . 3
1.2.1 AC1 . . . 3
1.2.2 AC2 . . . 3
1.2.3 AC3 . . . 4
1.3 Start of the survey . . . 7
1.3.1 Control/Experimenter conditions . . . 7
1.3.2 Promise/Experimenter conditions . . . 7
1.3.3 Control/Peer conditions . . . 8
1.3.4 Promise/Peer conditions . . . 8
1.4 Guessing game: obfuscated conditions . . . 11
1.5 End of the survey . . . 12
1.5.1 Experimenter/Clear conditions . . . 12
1.5.2 Experimenter/Obfuscated conditions . . . 12
1.5.3 Peer/Clear conditions . . . 13
1.5.4 Peer/Obfuscated conditions . . . 13
1.6 Manipulation check . . . 16

List of Figures

S24 Attention check 1 Study 3 . . . 5
S25 Attention check 2 Study 3 . . . 6
S26 Attention check 3 Study 3 . . . 6
S27 Start of Study 3: Promise/Experimenter conditions . . . 9
S28 Start of Study 3: Control/Peer conditions . . . 10
S29 Study 3: Guessing game . . . 11
S30 Study 3: Payback decision (Experimenter/Visible) . . . 14
S31 Study 3: Payback decision (Peer/Obfuscated) . . . 15
S32 Study 3: Manipulation check . . . 16

1 Study 3: Survey conditions and material

1.1 MTurk Specification

- **Announced timespan**: 10–15 minutes
- **Fixed compensation**: $1.50
- **Bonus payment**: $0.00 to $0.20
- **Time allotted**: 2 hours
- **Auto-approval**: 36 hours
- **Filters**: Hit approval rating $\geq$ 96%, Location: US
- **Attention check:** yes (two out of three correct to pass)
- **VPS/VPN/proxy check:** yes (both false locations and use of server farms prevented via iphub.info)

1.2 Attention checks

Participants had to pass two out of three attention checks (see Fig. S24, Fig. S25, and Fig. S26 for screenshots.

1.2.1 AC1.

How do you call the fruit shown above?

- Tomato
- Aubergine *(accepted as correct)*
- Brinjal
- Pineapple
- Berenjena
- Berinjela
- Eggplant *(accepted as correct)*
- Potato

1.2.2 AC2.

Please check all options that are NOT names of US states.

- Maryland
- New Hampshire
- North Dakota
- New Wyoming *
- New York
- New Jersey

- New Mexico
- North Carolina
- North Montana *
- Idaho

Participants passed the check only if "New Wyoming" and "North Montana" were checked, but nothing else.

1.2.3 AC3.

This is a test of your actually reading the questions. To pass this test, answer the following question by entering the word bookbinder without any capitalized letter in the field below, nothing else (no spaces). As you can see, we are interested in certain reading habits.

What is your **favorite book** at the moment (including non-fiction)?

Participants passed the check if the entry text contained the string "bookbinder". This also counted the following answers as correct "word bookbinder", "the word bookbinder" or "theword-bookbinder".

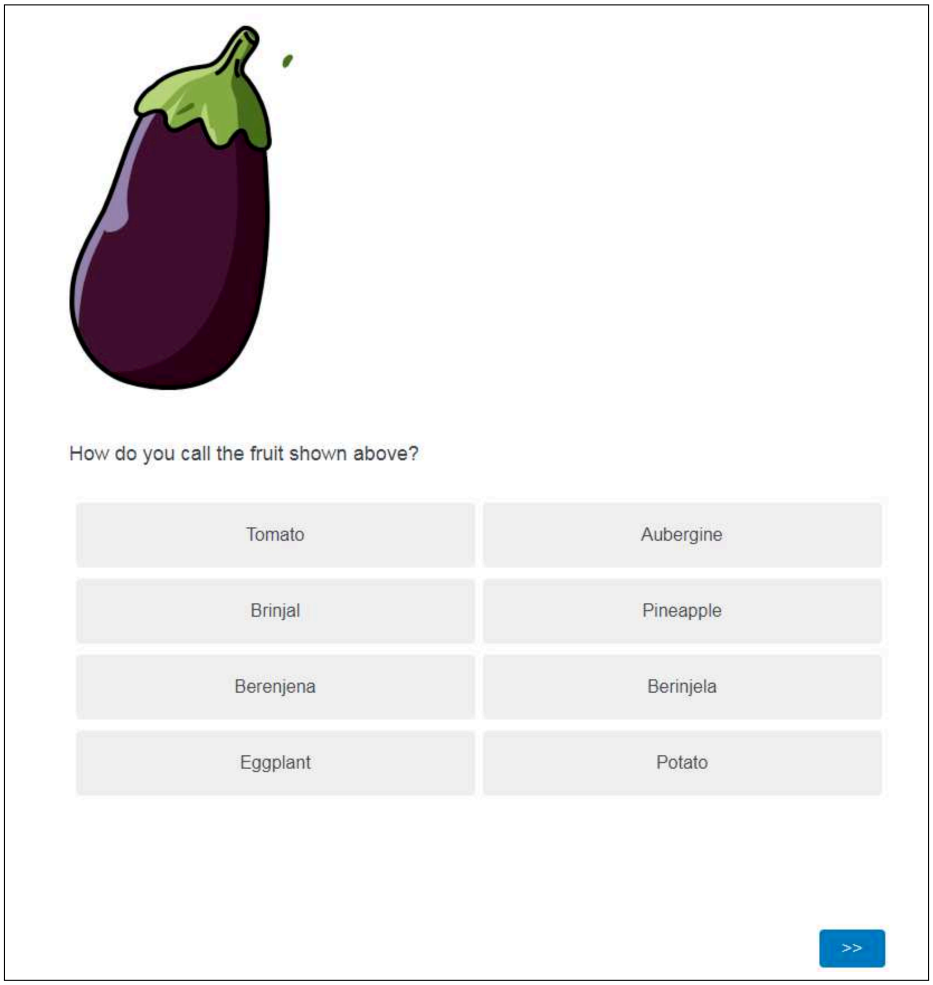

Figure S24. First attention check in Study 3: Aubergine and Eggplant were accepted as correct answers.

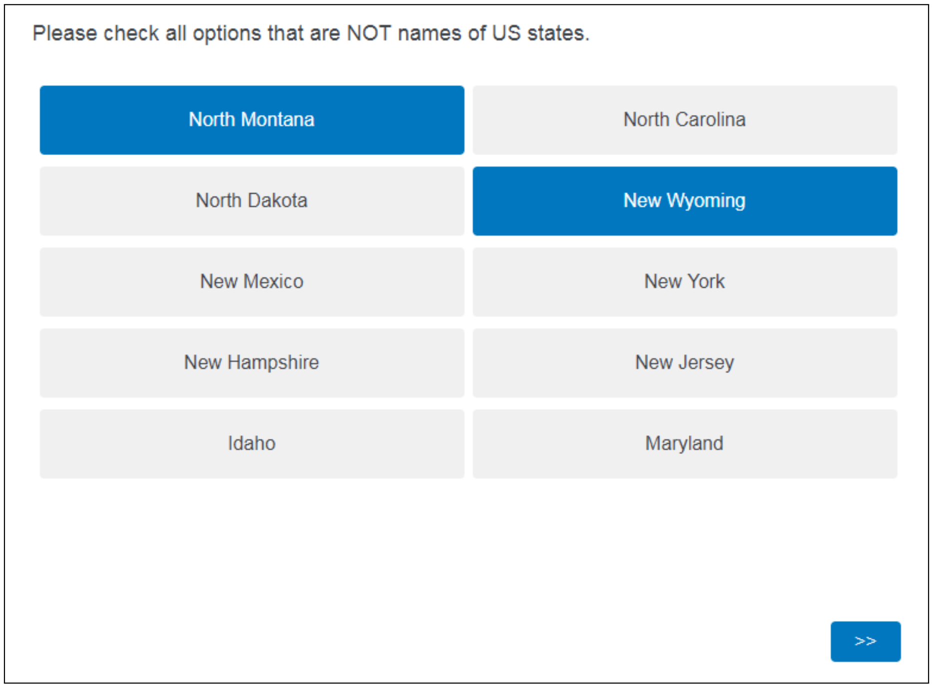

Figure S25. Second attention check in Study 3: State names were presented in randomized order.

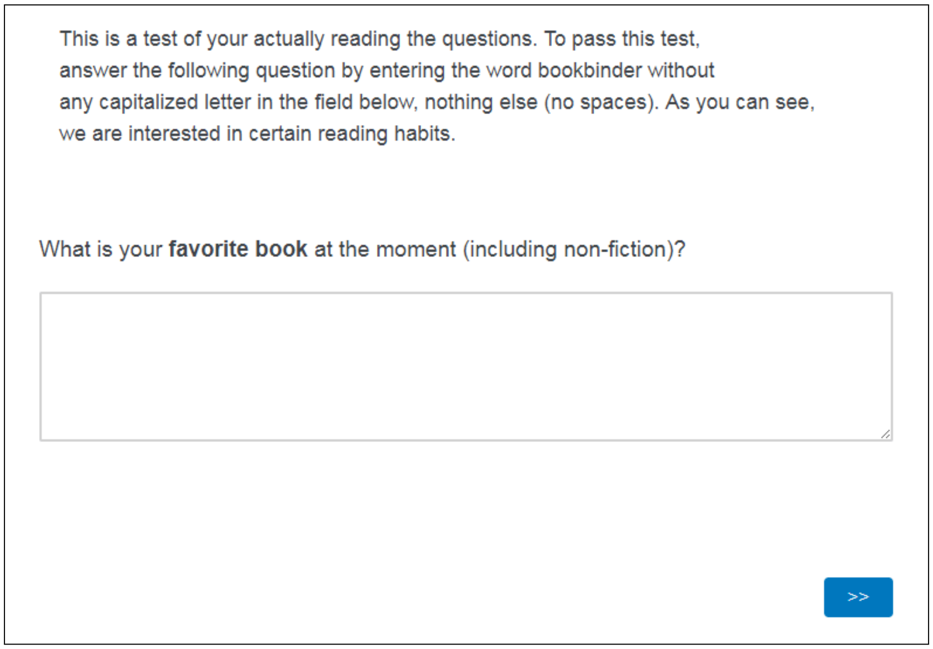

Figure S26. Third attention check in Study 3: The text block was presented as an image file.

1.3 Start of the survey

[Note that the start of the study is independent of the level of the obfuscation factor].

1.3.1 Control/Experimenter conditions.

You have two options that have consequences for your bonus payment. You can choose between two amounts of money. The money you receive will be taken from the requester and paid as bonus payment.

1. You can now receive 5 cents from the requester without any further consequences.
2. You can now receive 20 cents from the requester. In this case we would give you a chance to give back 10 cents to the requester at the end of this study. (You will be able to determine how much you want to pay back).

Your current bonus money is shown at the top of the screen and paid out after submitting the HIT.

[*Participants were offered two options in check boxes:*]

- I take 5 cents from the requester.
- I take 20 cents from the requester and I can decide later, at the end of this study, whether I want to pay back 10 cents to the requester.

1.3.2 Promise/Experimenter conditions. *See Figure S27 for a screen shot.*

You have two options that have consequences for your bonus payment. You can choose between two amounts of money. The money you receive will be taken from the requester and paid as bonus payment.

1. You can now receive 5 cents from the requester without any further consequences.
2. You can now receive 20 cents from the requester. In this case we would ask you to promise that you will give back 10 cents to the requester at the end of this study. (You will be able to determine how much you want to pay back).

Your current bonus money is shown at the top of the screen and paid out after submitting the HIT.

[*Participants were offered two options in check boxes:*]

- I take 5 cents from the requester.
- I take 20 cents from the requester and I promise to pay back 10 cents to the requester later, at the end of this study.

1.3.3 Control/Peer conditions. *See Figure S28 for a screen shot.*

For this task, you will be paired with another Turker who will take part in a different study.

You have two options that have consequences for your bonus payment and the bonus payment of the other Turker (whose decisions cannot change your own bonus payment). You can choose between two amounts of money. The money you receive will be taken from the bonus payment of the other Turker.

1. The other Turker will start with 10 cents of extra bonus payment. You can now receive 5 cents from the other Turker without any further consequences.
2. The other Turker will start with 20 cents of extra bonus payment. You can now receive 20 cents from the other Turker. In this case we would give you a chance to give back 10 cents to the other Turker at the end of this study. (You will be able to determine how much you want to pay back).

The other Turker will be informed about the bonus money at the start, the money you take and the money you will pay back. Your current bonus money is shown at the top of the screen and paid out after submitting the HIT.

[*Participants were offered two options in check boxes:*]

- I take 5 cents from the other Turker.
- I take 20 cents from the other Turker and Ican decide later, at the end of this study, whether I want to pay back 10 cents to the other Turker.

1.3.4 Promise/Peer conditions.

For this task, you will be paired with another Turker who will take part in a different study.

You have two options that have consequences for your bonus payment and the bonus payment of the other Turker (whose decisions cannot change your own bonus payment). You can choose between two amounts of money. The money you receive will be taken from the bonus payment of the other Turker.

1. The other Turker will start with 10 cents of extra bonus payment. You can now receive 5 cents from the other Turker without any further consequences.
2. The other Turker will start with 20 cents of extra bonus payment. You can now receive 20 cents from the other Turker. In this case we would ask you to promise that you will give back 10 cents to the other Turker at the end of this study. (You will be able to determine how much you want to pay back).

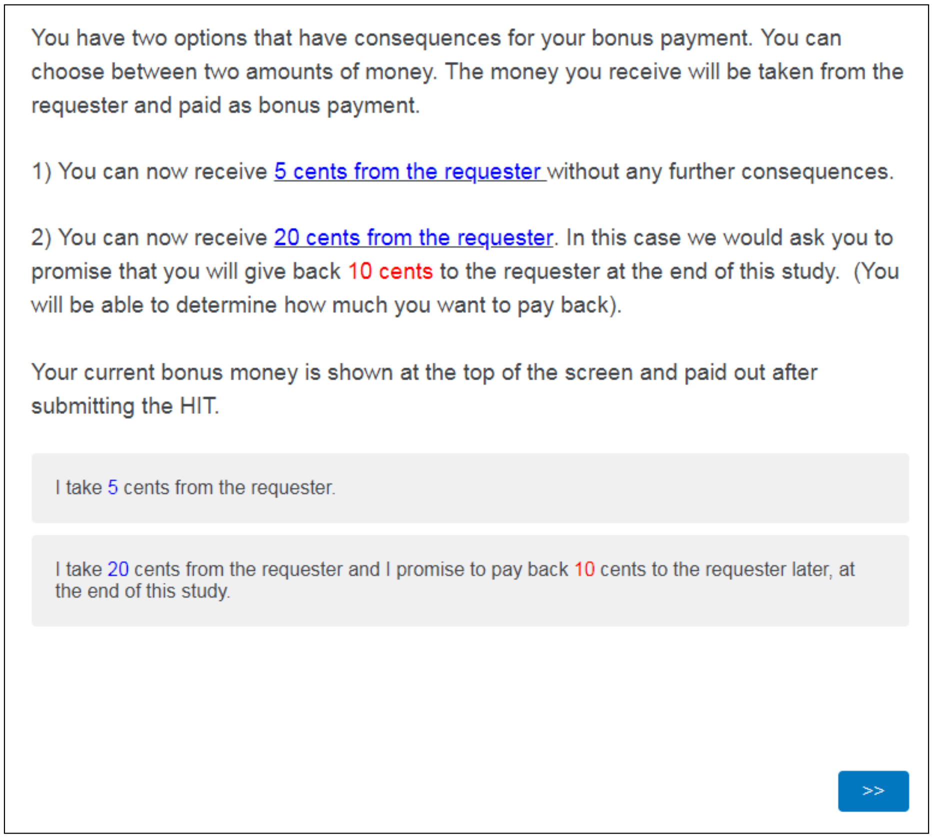
You have two options that have consequences for your bonus payment. You can choose between two amounts of money. The money you receive will be taken from the requester and paid as bonus payment.

1) You can now receive 5 cents from the requester without any further consequences.

2) You can now receive 20 cents from the requester. In this case we would ask you to promise that you will give back 10 cents to the requester at the end of this study. (You will be able to determine how much you want to pay back).

Your current bonus money is shown at the top of the screen and paid out after submitting the HIT.

I take 5 cents from the requester.

I take 20 cents from the requester and I promise to pay back 10 cents to the requester later, at the end of this study.

>>

Figure S27. Start of Study 3 in the Promise/Experimenter conditions

The other Turker will be informed about the bonus money at the start, the money you take, your promise (if you decide to make it) and the money you will pay back. Your current bonus money is shown at the top of the screen and paid out after submitting the HIT.

[*Participants were offered two options in check boxes:*]

- I take 5 cents from the other Turker.
- I take 20 cents from the other Turker and I promise to pay back 10 cents to the other Turker later, at the end of this study.

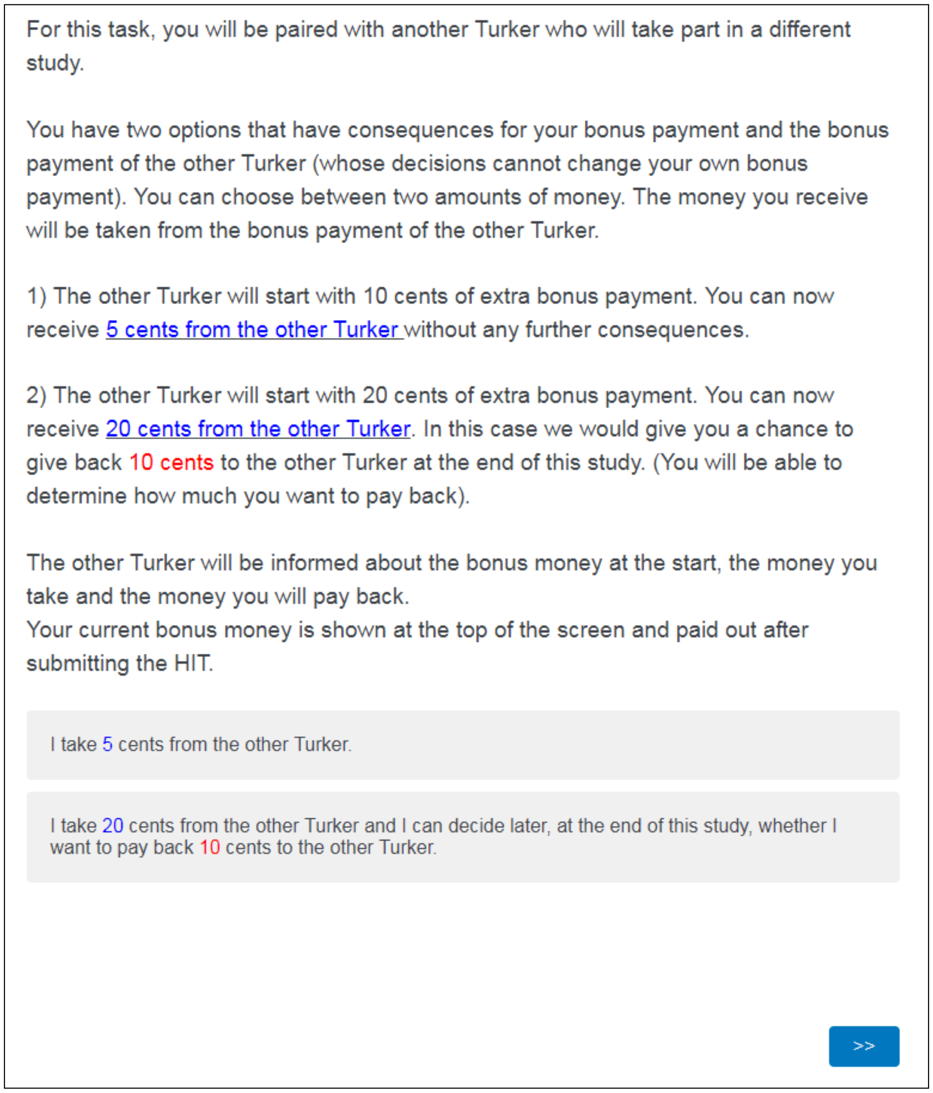

For this task, you will be paired with another Turker who will take part in a different study.

You have two options that have consequences for your bonus payment and the bonus payment of the other Turker (whose decisions cannot change your own bonus payment). You can choose between two amounts of money. The money you receive will be taken from the bonus payment of the other Turker.

1) The other Turker will start with 10 cents of extra bonus payment. You can now receive 5 cents from the other Turker without any further consequences.

2) The other Turker will start with 20 cents of extra bonus payment. You can now receive 20 cents from the other Turker. In this case we would give you a chance to give back 10 cents to the other Turker at the end of this study. (You will be able to determine how much you want to pay back).

The other Turker will be informed about the bonus money at the start, the money you take and the money you will pay back.
Your current bonus money is shown at the top of the screen and paid out after submitting the HIT.

I take 5 cents from the other Turker.

I take 20 cents from the other Turker and I can decide later, at the end of this study, whether I want to pay back 10 cents to the other Turker.

>>

Figure S28. Start of Study 3 in the Control/Peer conditions

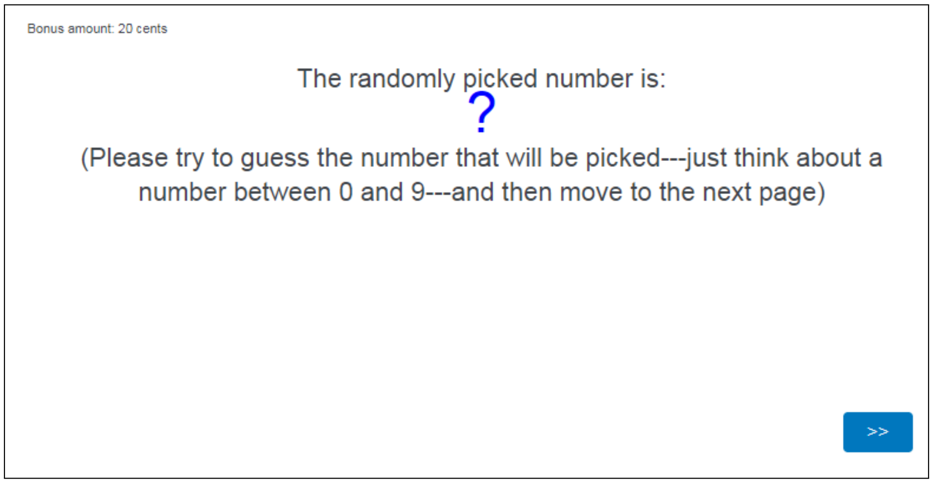

Figure S29. Guessing game in Study 3

1.4 Guessing game: obfuscated conditions

[See Figure S29 for a screenshot.]

In this part, we include a guessing game that involves a random number generator. Your task is to guess which number between 0 and 9 (0, 1, 2, 3, ..., 9) will be chosen by the number generator. We do not ask you to enter the number you choose, so you are the only one to know (and we have no way of knowing) whether you have guessed correctly or not.

[*New Page*]

The randomly picked number is:
?
(Please try to guess the number that will be picked—just think about a number between 0 and 9—and then move to the next page)

[*New Page, the number is drawn randomly between 0 and 9.*]

The randomly picked number is:
3

[*New Page*]

If you guessed correctly, then remember that you have won the guessing game.
If you have not guessed correctly, then remember that you have lost the guessing game.
We will not ask you whether you have won or lost the game.

1.5 End of the survey

[Note that the end of the study is independent of the level of the Promise/Control factor].

1.5.1 Experimenter/Clear conditions. *[See Figure S30 for a screenshot.]*

We gave you a choice at the beginning of the study between taking 5 cents or 20 cents from the requester.

You decided to take 20 cents from the requester.

We now ask you whether you want to pay back money to the requester. We will not force you to pay back any money, you can pay back any amount between 0 cents and 20 cents.

How many cents do you pay back to the other requester?
(Please enter a number between 0 and 20.)
[Entry box for number]

Note that we collect data from more than a hundred Turkers in each variant of this experiment and we are interested in responses across the whole group.
Your response does not have any consequences for your bonus payment other than those described: There are no unannounced payments and no further tasks influencing your bonus payment.

1.5.2 Experimenter/Obfuscated conditions.

We gave you a choice at the beginning of the study between receiving 5 cents or 20 cents from the requester.

You decided to take 20 cents from the requester.

We now ask you whether you want to pay back money to the requester. We will not force you to pay back any money, you can pay back any amount between 0 cents and 20 cents.

If you have won the guessing game, just enter 0 below. You will not pay back any money.

How many cents do you pay back to the other requester?
(Please enter a number between 0 and 20.)
[Entry box for number]

Note that we collect data from more than a hundred Turkers in each variant of this experiment and we are interested in responses across the whole group.
Your response does not have any consequences for your bonus payment other than those described: There are no unannounced payments and no further tasks influencing your bonus payment.

1.5.3 Peer/Clear conditions.

We gave you a choice at the beginning of the study between taking 5 cents or 20 cents from the requester.

You decided to take 20 cents from the requester.

We now ask you whether you want to pay back money to the requester. We will not force you to pay back any money, you can pay back any amount between 0 cents and 20 cents.

How many cents do you pay back to the other Turker?
(Please enter a number between 0 and 20.)
[Entry box for number]

Note that we collect data from more than a hundred Turkers in each variant of this experiment and we are interested in responses across the whole group.
Your response does not have any consequences for your bonus payment other than those described: There are no unannounced payments and no further tasks influencing your bonus payment.

1.5.4 Peer/Obfuscated conditions. *[See Figure S31 for a screenshot.]*

We gave you a choice at the beginning of the study between taking 5 cents or 20 cents from another Turker.

You decided to take 20 cents from the other Turker.

We now ask you whether you want to pay back money to the other Turker. We will not force you to pay back any money, you can pay back any amount between 0 cents and 20 cents.

If you have won the guessing game, just enter 0 below. You will not pay back any money. The other Turker will be informed about the money transfers as described before and about the possible consequences of the guessing game.

How many cents do you pay back to the other Turker?
(Please enter a number between 0 and 20.)
[Entry box for number]

Note that we collect data from more than a hundred Turkers in each variant of this experiment and we are interested in responses across the whole group.
Your response does not have any consequences for your bonus payment other than those described: There are no unannounced payments and no further tasks influencing your bonus payment.

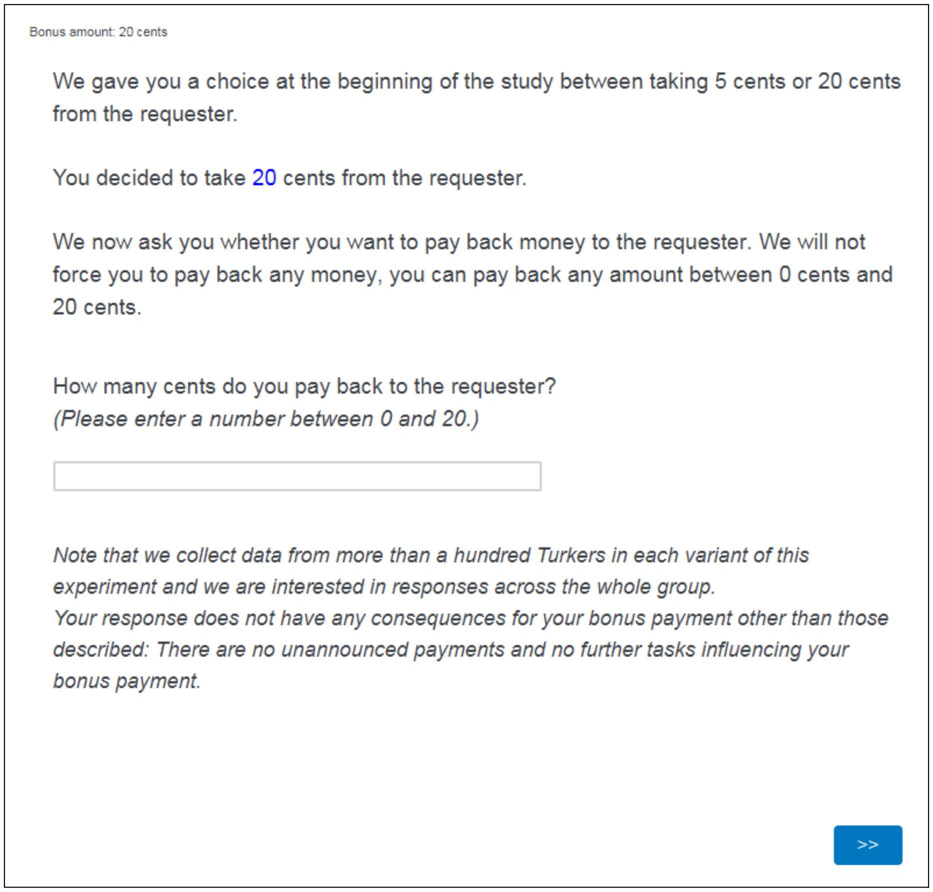

Bonus amount: 20 cents

We gave you a choice at the beginning of the study between taking 5 cents or 20 cents from the requester.

You decided to take 20 cents from the requester.

We now ask you whether you want to pay back money to the requester. We will not force you to pay back any money, you can pay back any amount between 0 cents and 20 cents.

How many cents do you pay back to the requester?
(Please enter a number between 0 and 20.)

Note that we collect data from more than a hundred Turkers in each variant of this experiment and we are interested in responses across the whole group.
Your response does not have any consequences for your bonus payment other than those described: There are no unannounced payments and no further tasks influencing your bonus payment.

>>

Figure S30. Payback decision in Experimenter/Clear conditions

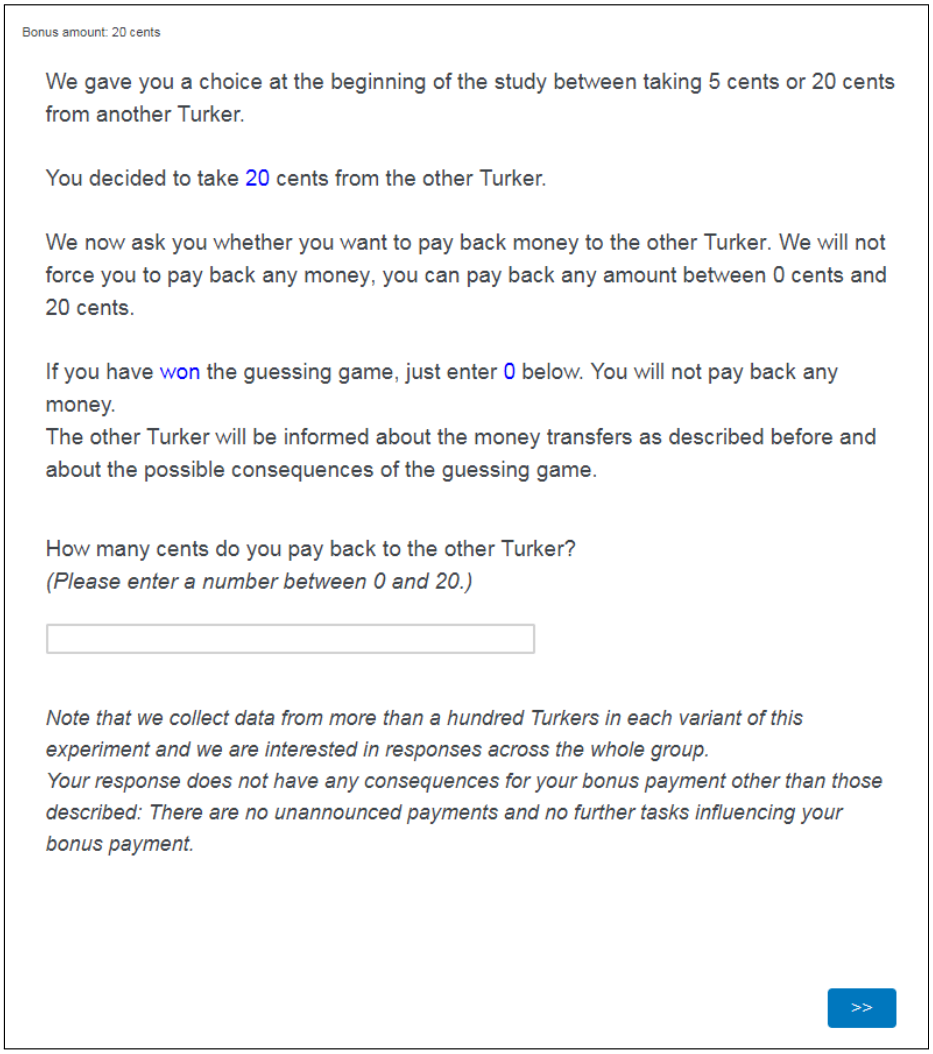

Figure S31. Payback decision in the Peer/Obfuscated conditions

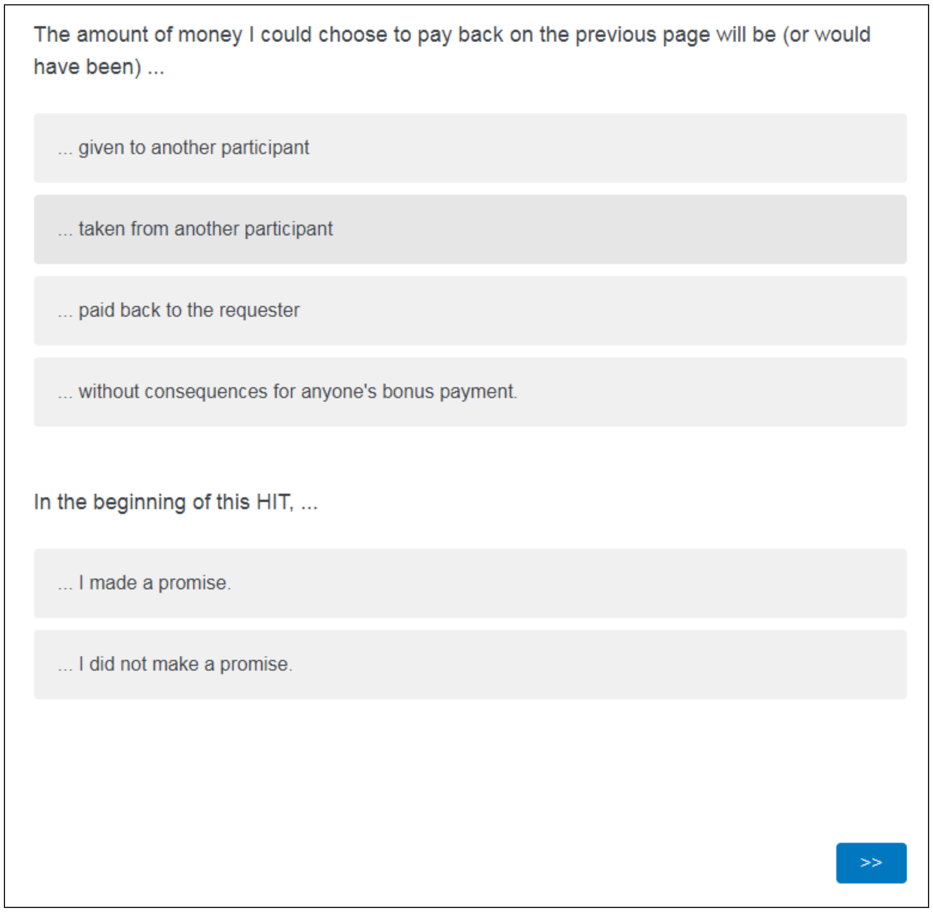

Figure S32. Manipulation check in Study 3

1.6 Manipulation check

See Figure S32 for a screen shot.

The amount of money I could choose to pay back on the previous page will be (or would have been) ...[*answer options were presented as radio boxes*]

- ... given to another participant [*correct response in peer conditions*]
- ... taken from another participant
- ... paid back to the requester [*correct response in experimenter conditions*]
- ... without consequences for anyone's bonus payment.

In the beginning of this task... [*answer options were presented as radio boxes*]

- I made a promise [*correct response in promise conditions*]
- I did not make a promise [*correct response in control conditions*]
