# Supplementary material for: Most People Keep Their Word Rather Than Their Money
Source: Open Mind (Camb). 2019 Jul 1;3:68–88. doi: 10.1162/opmi_a_00027 (PMC8412196; doi:10.1162/opmi_a_00027)
Supplement: Supplementary file 4 [file opmi-03-68-s004.pdf]

Supplementary Material (4/4) for "Most People Keep Their Word Rather Than Their Money"

Scoring Rule, Qualitative Data, Additional Figures

Jan K. Woike
Max-Planck-Institute for Human Development, Berlin

Patricia Kanngiesser
Freie Universität Berlin

Compiled on May 2nd, 2019

Contents

1 Specification of the quadratic scoring rule **4**

2 Qualitative data coding in Study 1a **6**
2.1 Coding method and reliability . . . 6
2.2 Categories and examples . . . 6
2.2.1 Choosing the lower payment . . . 7
2.2.1.1 Examples for main category 31: Avoid consequences . . . 7
2.2.1.2 Examples for main category 32: Various . . . 7
2.2.2 Paying back 5 cents . . . 7
2.2.2.1 Examples for category 11: Norms . . . 7
2.2.2.2 Examples for category 12: Moral evaluation . . . 10
2.2.2.3 Examples for category 13: Personal values . . . 10
2.2.2.4 Examples for category 14: Anticipated emotions . . . 13
2.2.2.5 Examples for category 15: Other-regarding preferences . . 13
2.2.2.6 Examples for category 16: Money-focused reasons . . . 13
2.2.2.7 Examples for category 17: Consequences . . . 13
2.2.2.8 Examples for category 18: Atonement . . . 13
2.2.3 Not paying back 5 cents . . . 13
2.2.3.1 Examples for category 21: Free choice . . . 13
2.2.3.2 Examples for category 23: No moral problem . . . 16
2.2.3.3 Examples for category 25: No reason . . . 16
2.2.3.4 Examples for category 28: Surprise . . . 16
2.3 Coding results . . . 17

3 Selected comments after Study 3 **20**

4 Supplementary analyses and figures **22**
4.1 Distribution of payback amounts across Studies 1a, 2a and 3 . . . 22
4.2 Responses to the post-questionnaire in Study 2a . . . 23
4.3 PANAS results . . . 27

List of Figures

S33 QSR example in Study 2b . . . 5
S34 Distribution of payback amounts across studies . . . 22
S35 Postquestionnaire: Not accepted . . . 24
S36 Postquestionnaire: Promise kept . . . 25
S37 Postquestionnaire: Promise broken . . . 25
S38 Postquestionnaire: Paid back money . . . 26
S39 Postquestionnaire: Did not pay back money . . . 26
S40 Means and CIs for PANAS in Study 1a . . . 28
S41 Means and CIs for PANAS in Study 2a . . . 29

List of Tables

S1 Quadratic scoring rule examples 4
S2 Coding statistics 6
S3 Codes and categories: Not accepted 8
S4 Codes and examples: Not accepted 9
S5 Codes and categories: Paid back 11
S6 Codes and examples: Paid back 12
S7 Codes and categories: Not paid back 14
S8 Codes and examples: Not paid back 15
S9 Categorizations: Not accepted 17
S10 Categorizations: Paid back 18
S11 Categorizations: Not paid back 19

1 Specification of the quadratic scoring rule

The quadratic scoring rule assigns values between -1 and +1 to k intervals with values $v_1, ..., v_k$ and $\sum_i v_k = 1$. An interval with value v_i is assigned the score

$$S_i = 2 \cdot v_i - \sum_j v_j{}^2. \quad (1)$$

Table S1 shows a number of examples for value distributions and corresponding scores and allows to illustrate some properties of the score: Scores are independent of the number of intervals with zero values (see Ex.2 vs. Ex.3, Ex.8 vs. Ex. 9) or the order of values (see Ex.4 vs. Ex.5). Moving away from an equal distribution of points (Ex. 1 and Ex. 14) increases the score of alternatives with higher than average values, but decreases the score of alternatives with lower than average values even more. The sum of scores is at a maximum for equal distributions, and the more values are concentrated, the sharper the contrast becomes (see Ex.14 up to Ex.9).

This translates into the following desirable properties for the task. If the participant subjectively believes that all intervals are equally likely, then the expected value would be at a maximum for an equal distribution of points. There is an expected loss for gambling on certain values over others. For a loss-averse participant, the expected utility is at a maximum when the point allocation corresponds to the subjective probability distribution.

To determine payoffs, the distributed points in each interval were divided by 100, and the scores were multiplied by \$0.50 . Example 7 corresponds to the distribution of values shown in Figure S33 (omitting the intervals with zero points assigned), which was shown to participants. The payment amounts shown in the figure correspond to $S_i \cdot \$0.50$, which explains the shown payment amounts of -3 cents ($-0.06 \cdot \$0.50 = \$-0.03$), 7 cents ($0.14 \cdot \$0.50 = \$0.07$), and 27 cents ($0.54 \cdot \$0.50 = \$0.27$).

Table S1
Quadratic scoring rule examples

		Values					Scores				
Ex.	k	v_1	v_2	v_3	v_4	v_5	S_1	S_2	S_3	S_4	S_5
1	5	0.2	0.2	0.2	0.2	0.2	0.2	0.2	0.2	0.2	0.2
2	5	0.5	0.5	0	0	0	0.5	0.5	-0.5	-0.5	-0.5
3	3	0.5	0.5	0	-	-	0.5	0.5	-0.5	-	-
4	5	0	0.1	0.4	0.5	0	-0.42	-0.22	0.38	0.58	-0.42
5	5	0.5	0.4	0.1	0	0	0.58	0.38	-0.22	-0.42	-0.42
6	5	1	0	0	0	0	1	-1	-1	-1	-1
7	5	0.1	0.2	0.4	0.2	0.1	-0.06	0.14	0.54	0.14	-0.06
8	2	0.9	0.1	-	-	-	0.98	-0.62	-	-	-
9	3	0.9	0.1	0	-	-	0.98	-0.62	-0.82	-	-
10	3	0.9	0.05	0.05	-	-	0.985	-0.715	-0.715	-	-
11	3	0.8	0.1	0.1	-	-	0.94	-0.46	-0.46	-	-
12	3	0.5	0.3	0.2	-	-	0.65	0.25	-0.15	-	-
13	3	0.4	0.3	0.3	-	-	0.46	0.26	0.26	-	-
14	3	$0.\bar{3}$	$0.\bar{3}$	$0.\bar{3}$	-	-	$0.\bar{3}$	$0.\bar{3}$	$0.\bar{3}$	-	-

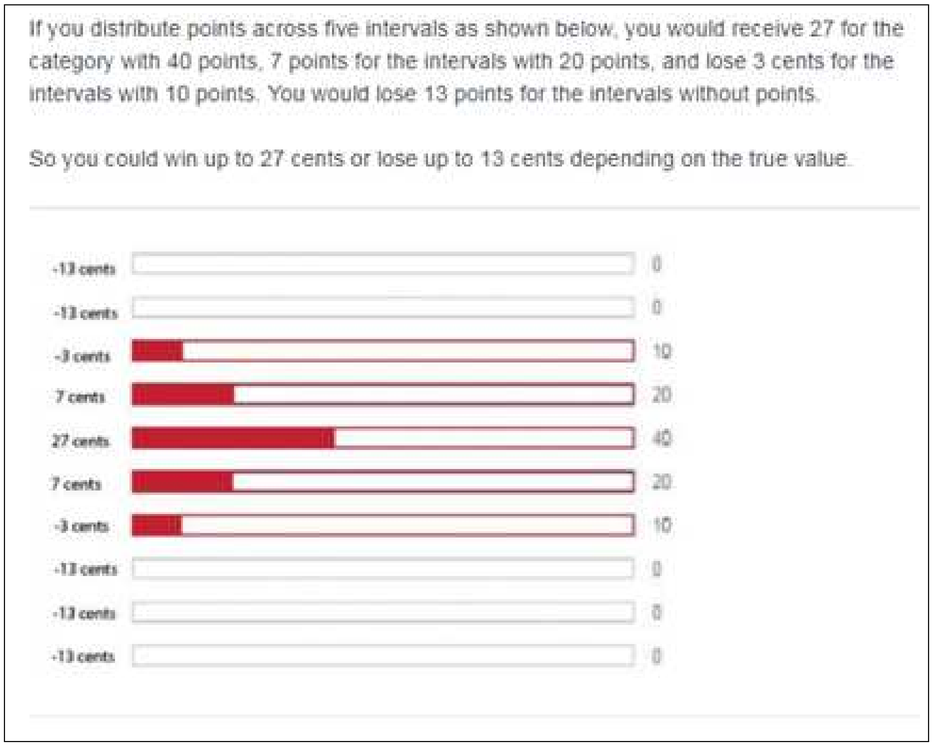

Figure S33. Quadratic scoring rule example in Study 2b

2 Qualitative data coding in Study 1a

All comments given by participants were sorted into three categories based on the participants' decisions: (1) Comments given after choosing the lower payments (and therefore skipping the final part, see Table S3), (2) Comments given after paying back 5 cents or more, (3) Comments given after paying back less than 5 cents. Comments were collected across conditions, but two independent coders worked on comment lists that did not contain condition information (only category and payment information). A preliminary coding scheme was developed and extended by the first coder, where necessary. The coding scheme had main categories and subcategories for each of the three comment types.

2.1 Coding method and reliability

All comments were split into the three categories as detailed above. Two independent coders classified all comments without access to the experimental condition the participants were in. A categorization scheme was developed by the authors and modified by the first coder, where necessary. Each coder could classify each comment into up to three categories consisting of main category and subcategory. The first author went through the complete list of codings. Table S2 summarizes the overlap of categorizations between the two coders and how disputes were resolved by the first author.

Table S2
Coding statistics

Type of match	N	Perc.	Third party decision
Complete mismatch	27	5.1%	dropped
No codes chosen	3	0.6%	dropped
Only one code	13	2.4%	dropped
Only primary code match	27	5.1%	one supplementary chosen
Additional primary category	88	16.5%	additional dropped
Additional secondary category	32	6.0%	additional dropped/decision for 12 cases
Complete match	342	64.3%	unchanged
Total	532	100%	

For 64.3% of the cases, both coders agreed on (all) main and sub-categories used. In about 23% of the cases, at least one set of primary and secondary category matched. In these cases, additional main categories or subcategories offered by only one coder were dropped. If a joint subcategory was lacking (in 5% of the cases), this subcategory was chosen by the first author. A complete mismatch occurred for 5.1% of the comments. For these cases, no categories were coded.

2.2 Categories and examples

Here, we present the lists of categories that were used to categorize the open statements in the three categories. For each category, we present tables of categories with their

definitions and examples given to coders. This is followed by lists of examples chosen among participants' comments that were categorized in the respective category.

2.2.1 Choosing the lower payment. Table S3 shows codes and definitions for categorizing comments from participants that did not accept the higher payment. Table S4 presents examples that were given to coders as anchors for each subcategory.

In the following, we present selective examples for main categories (omitting empty or near-empty categories).

2.2.1.1 Examples for main category 31: Avoid consequences.

- I go for the sure thing
- Because I was not sure that I would receive the extra money.
- there really wasnt much of a difference and i also know i m guaranteed the .05
- I would prefer the set amount and not have to worry, or decide to give some back.
- If I took the five cents I knew exactly what I was getting. It seemed like maybe I should pick the option where I paid back, but I was wondering if there was some task that would involve me losing more money than that, and not having the five cents to give back.
- You're asking me to remember to do something for what is basically a 5 cent bonus. What if I forget? Is there another penalty for forgetting? I'll pass on the risk, the reward is too small. :)
- I did not want to make the promise and receive more money because I am not aware of the consequences that are involved with the 15 cents. I feel like the 5 cents was a "no strings attached" deal.
- I felt like there may be strings attached later – preferred the option with no consequences.
- Theres a catch always a catch

2.2.1.2 Examples for main category 32: Various.

- I didn't want to forget about paying the five cents back and risk getting my HIT rejected.
- It sounded more complicated than what I wanted to deal with.
- I just figured that it would be simpler to just take the 5 cents and leave it at that.
- It's less complicated.
- I hate owing people because I may not have the money to pay back in the end.
- WHY BECAUSE I LIKE THE EASIER WAY BETTER I DONT LIKE TO MAKE A PROMISE

2.2.2 Paying back 5 cents. Table S5 shows codes and definitions for categorizing comments from participants that paid back 5 cents at the end of the survey. Table S6 presents examples that were given to coders as anchors for each subcategory.

In the following, we present selective examples for main categories (omitting empty or near-empty categories).

2.2.2.1 Examples for category 11: Norms.

- Because I made a promise, therefore that money isn't mine.
- I made a promise and I intend to keep it; it is the morally and ethically correct thing to do
- I've always felt it was better to keep my word on promises as that makes me feel like my morals are in check.
- I made a promise to pay this amount and I will not go back against my word. When I give my word, I will not be swayed to do the contrary.
- Promises should not be broken, no matter how insignificant they may appear to be.

Table S3

Codes and category descriptions for open answers of participant who did not accept the higher payment

ID1	Main category	ID2	Sub category	Longer Description
**31**	Avoid possible consequences	31.1	Prefers certainty/ the sure thing	Participants state that they preferred the sure thing and/or wanted to avoid risks.
		31.2	wants to avoid consequences	Participants state that they thought there would be/wanted to avoid consequences.
		31.3	Suspects hidden agenda	Participants state that they feared there was a hidden agenda or strings attached.
**32**	Various reasons	32.1	fear of forgetting to pay back	Participants state that they feared that they would forget paying back the money.
		32.2	option is easier/less troublesome	Participants state that they wanted the easy option, no hazzle or trouble.
		32.3	not enough incentive for larger amount	Participants state that the incentive was too small to take the larger amount
**33**	Avoid commitment	33.1	does not want to owe money	Participants state that they did not want to (or like to) owe money.
		33.2	does not want to make promise	Participants state that they didn?t want to promise.
**34**	Unknown why	34		Participants state they don't know why they chose the option.
**35**	Technical/personal error	35		Participants state that they accidentally chose the option.

Table S4

Codes and category examples for open answers of participant who did not accept the higher payment

ID1	Main category	ID2	Sub category	Examples
31	Avoid possible consequences	31.1	Prefers certainty/ the sure thing	I wanted the sure thing.”
		31.2	wants to avoid consequences	‘ “I didn’t want any negative consequences.”
		31.3	Suspects hidden agenda	“I thought there would be strings attached.” “I thought there was a hidden agenda.”
32	Various reasons	32.1	fear of forgetting to pay back	“I may forget.” “I thought I will not remember.”
		32.2	option is easier/less troublesome	“It sounded too complicated.”, “I wanted the easy option.”
		32.3	not enough incentive for larger amount	“The difference was too small.” “5 was enough.”
33	Avoid commitment	33.1	does nto want to owe money	“I don’t like owing money.”
		33.2	does not want to make promise	“I don’t want to promise.”
34	Unknown why	34		“I don’t know.”
35	Technical/personal error	35		“the page wouldn’t let me forward.”

- A promise is a promise. I don't break promises. That's how you keep someones trust, and how you maintain healthy relationships with whoever you come across.
- It is simpler, on many levels, to keep promises, than to live with the schism of the integrity of my self that is created by breaking a promise. The same goes for telling the truth as best I can, as the first person that gets lied to by a liar is themselves and at the point they've convinced themselves to some degree that the lie is true enough to convince others they are telling the truth, they have chosen to divorce themselves from reality. But since a part of them knows what really happened, they have fractured their own integrity.
- I always keep my promises!
- Mutual agreements are the basis of social contracts. A social agreement, once agreed to, ought to be binding regardless of the circumstances – with very rare exceptions indeed. Once I had agreed, it's true that there was no force upon me to bind me, except my own moral values – but because I believe in the necessity of living up to one's word, and I wanted to remain consistent within myself, I chose to honor the agreement that I had made.
- I agreed to give back 0.05 so I will.
- i take what i state in these surveys seriously and i stated that i would pay the 5 cents back
- Honesty is important to me, I said I would so I will.
- I entered into a covenant with you of good faith and fair dealing. I don't want to breach the contract I made with you and violate any 'legal duties' or 'implied duties' to you.
- Because i explicitly made a promise to return the money i took so it was my obligation to give the money back.

2.2.2.2 *Examples for category 12: Moral evaluation.*

- I thought it would be morally right to pay back the amount asked.
- I was raised by my parents, relatives, teachers, and community to be an ethical person with a good moral compass. I would rather forego monetary gain to finish the test with a clear conscience than to take on the guilt of not keeping a promise in exchange for monetary gain. And my choice will remain the same, no matter whether the other participants in the game act honestly or dishonestly.
- It seemed like the right thing to do.
- It only seems fair to keep my word.
- All is fair. A deal is a deal.
- It was the right thing to do

2.2.2.3 *Examples for category 13: Personal values.*

- I am a person of integrity–if I say I'll do something, then I do it.
- Those are the terms I agreed to and I believe in holding up my end of the bargain as an honest person
- it is important for society that people be honest with each other
- That's the kind of person I am. I like to keep my word.
- I am a honest person.
- Because I am nothing if I cannot give my word and stand by it.
- I think it's always important to keep a promise. Even though it's a small amount, it's not the amount that's important, it's the principle behind keeping a promise or not. I want to be a person who can be counted on to keep a promise.

Table S5

Codes and category descriptions for open answers of participant who paid back money

ID1	Main category	ID2	Sub category	Longer Description
**11**	Covenant/norms as reason for paying back	**11.1**	Promise	Participant mentions promise/promise keeping
		**11.2**	Agreement	Participant mentions agreement
		**11.3**	Keep word	Participant mentions wanting to keep their word
		**11.4**	Contract	Participant mentions contract/deal
		**11.5**	Obligation	Participant mentions obligation/duty to pay back
		**11.6**	Rules	Participant mentions wanting to play by the rules
		**11.7**	Request	Participant mentions being asked/expected to do it, (task requirement)
**12**	Moral evaluation	**12.1**	Moral	Participant explicitly mentions morality/moral
		**12.2**	Right thing	Participant mentions that it was the right/correct thing to do
		**12.3**	Fairness	Participant mentions fairness or fair behaviour
**13**	Personal values	**13.1**	Integrity	Participant refers to personal integrity
		**13.2**	Honour	Participant refers to personal honour
		**13.3**	Honesty	Participant refers to (personal) honesty
		**13.4**	Word	Participant refers to being someone who keeps his/her word
		**13.5**	Trust	Participant mentions personal trust/trustworthiness
		**13.6**	Principles	Participant mentions having personal principles or values
		**13.7**	Generosity	Participant mentions personal generosity
**14**	Anticipated/felt emotions as reasons	**14.1**	Guilt	Participant mentions feeling guilty if s/he would break agreement/promise
		**14.2**	Pleasure	Participant mentions it felt good/pleasurable to keep the promise
		**14.3**	Disappointment	Participant mentions wanting to avoid feeling disappointed
**15**	Other regarding preferences	**15**		Participant mentions wanting to share or giving to someone who may need it
**16**	money-focused reason	**16.1**	good deal (even with payback)	Participant mentions that they are happy they ended up with more etc.
		**16.2**	not enough money to break promise	Participant mentions that it was not enough money to be tempted to break word
**17**	Fear of negative/hope for positive consequences	**17.1**	Anticipated punishment	Participant mentions that they feared negative consequences or punishment
		**17.2**	Anticipated reward	Participants mention they thought there may be a reward
		**17.3**	Playing it safe	Participant mentions they did not know what to expect
		**17.4**	Karma	Participant mentions believing in good karma, that it would pay back eventually etc.
**18**	"Atonement"/balancing out past behavior	**18**		Participant mentions wanting to make up for earlier errors and/or behaviour

Table S6

Codes and category examples for open answers of participant who paid back money

ID1	Main category	ID2	Sub category	Examples
**11**	Covenant/norms as reason	**11.1**	Promise	"I promised.", "I keep my promises."
	for paying back	**11.2**	Agreement	"I entered an agreement with you.", "I agreed to it."
		**11.3**	Keep word	"I always keep my word.", "I like to follow through on what I said"
		**11.4**	Contract	"We had a deal." "There was a contract."
		**11.5**	Obligation	"It was my obligation to pay it back."
		**11.6**	Rules	"I wanted to follow the rules."
		**11.7**	Request	"You asked me to.", "I was asked to.", "It was a requirement."
**12**	Moral evaluation	**12.1**	Moral	"It's the moral thing to do.", "I want to keep my morals in check."
		**12.2**	Right thing	"It's the right thing to do."
		**12.3**	Fairness	"It's the fair thing to do.", "I thought it was fair.", "It's the nice thing to do."
**13**	Personal values	**13.1**	Integrity	"I am a person of integrity."
		**13.2**	Honour	"I am an honourable individual."
		**13.3**	Honesty	"I prefer to be honest.", "I don't like to lie.",
		**13.4**	Word	"I am a woman of my word."
		**13.5**	Trust	"I believe in being trustworthy.", "I don't like breaking trust."
		**13.6**	Principles	"I have my principles.", "It's part of my values."
		**13.7**	Generosity	"I am a generous person."
**14**	Anticipated/felt emotions	**14.1**	Guilt	"I would feel guilty for not giving it back."
	as reasons	**14.2**	Pleasure	"It feels good to keep a promise.", "I take pleasure from keeping a promise."
		**14.3**	Disappointment	"I would feel disappointed about myself."
**15**	Other regarding preferences	**15**		"I wanted to share.", "I wanted to make sure others got some funds, too."
**16**	money-focused reason	**16.1**	good deal (even with payback)	"Happy to have 10 instead of 5."
		**16.2**	not enough money to break promise	"It wasn't much money, so easy to keep word. "
**17**	Fear of negative/hope	**17.1**	Anticipated punishment	"I was afraid there would be consequences.", "I thought there would be strings attached."
	for positive consequences	**17.2**	Anticipated reward	"I hoped for a reward...'
		**17.3**	Playing it safe	"I didn't know what was coming."
		**17.4**	Karma	"It's worth good karma."
**18**	"Atonement"/balancing	**18**		"I thought I was greedy in other parts of the survey."
	out past behavior			

2.2.2.4 *Examples for category 14: Anticipated emotions.*

- I would feel guilty if I did not keep the promise of paying the money back.
- I'd feel guilty lying after making an agreement.

2.2.2.5 *Examples for category 15: Other-regarding preferences.*

- just in case it is needed to someone else
- To make sure others get funds too

2.2.2.6 *Examples for category 16: Money-focused reasons.*

- it is all money i didn't have before, no need to be greedy
- costless....it's only 0.05. not worth being dishonest about it.
- 5 cents is well below the threshold for the amount of monetary gain that it would take for me to intentionally break my word.
- My honor is worth more than 5 cents.

2.2.2.7 *Examples for category 17: Consequences.*

- Because I felt that you might decline the bonus if I did not.
- Because I thought it might be taken away otherwise
- I'd like to say that I'm a noble person, but really I was just hoping for an additional bonus.

2.2.2.8 *Examples for category 18: Atonement.*

- I don't know. Maybe I thought I was a little greedy in other parts of the study.
- I feel I didn't do as well as I thought I was going to do.

2.2.3 Not paying back 5 cents. Table S7 shows codes and definitions for categorizing comments from participants that did not pay back 5 cents at the end of the survey. Table S8 presents examples that were given to coders as anchors for each subcategory.

In the following, we present selective examples for main categories (omitting empty or near-empty categories).

2.2.3.1 *Examples for category 21: Free choice.*

- I usually do the fair thing when it comes to decisions like this. But I wanted to see what would happen if I chose the greedy route.
- Well, duh......if given a choice, I won't give any money back. I was under the impression at the beginning of the study that I HAD to give 5 cents back. But since I don't, I won't.
- because i was given the choice to keep it.
- You freed me up. You said there was no obligation to pay it back (most recently).
- I was not required to pay anything back so I refused to pay anything back.
- If I am not being force to pay the money back, I would like to keep it.
- If I have the choice not to pay it back, why not? If there was a charge or reason why I needed to, I would have considered that. Since there wasn't, why not?

Table S7

Codes and category descriptions for open answers of participant who did not pay back money

ID1	Main category	ID2	Sub category	Longer Description
**21**	Free choice	21.1	Choice whether to pay or not	Participant mentions being given a choice, it being up to them etc.
		21.2	Not forced to pay back	Participant mentions not being forced to give it back
		21.3	does not want to pay	Participant mentions not wanting to give it back
		21.4	no penalty for not paying	Participants mention there being no penalty or negative consequences for keeping the money
		21.5	no obligation to pay	Participant mentions being under no obligation or duty to pay it back
**22**	No moral problem	22		Participants mention their behaviour not being wrong/ amoral/unethical/harmful
**23**	Self-regarding preferences	23.1	Need	Participant mentions needing the money
		23.2	Desert	Participant mentions deserving the money, working hard for it, etc.
		23.3	Selfishness/greed	Participant mentions being selfish or self-interested
**24**	Personal values	24		Participant cites personal values for decision.
**25**	No reason	25.1	lack reason	Participant mentions not being given a reason for paying back money
		25.2	lack incentive	Participant mentions seeing no benefit or incenctive in giving back money
		25.3	lack recipient	Participants mention there being no opportunity to donate the money or share with someone
**26**	Guilt	26		Participant mentions feeling guilty for keeping money
**27**	Frustration	27		Participant mentions being frustrated by other tasks
**28**	Surprise	28		Participants mention being wrongly accused of breaking their word/promise

Table S8

Codes and category examples for open answers of participant who did not pay back money

ID1	Main category	ID2	Sub category	Examples
**21**	Free choice	21.1	Choice whether to pay or not	“I was given a choice.”, “It was my choice.”
		21.2	Not forced to pay back	“I wasn’t forced to give anything back.”
		21.3	does not want to pay	“I do not want to pay anything back.”
		21.4	no penalty for not paying	“If there are no consequences for my decision...”
		21.5	no obligation to pay	“I wasn’t obliged to.”,
**22**	No moral problem	22		“There is nothing wrong.”, “No ethical issue keeping it....”, “...no harm to someone else...”
**23**	Self-regarding preferences	23.1	Need	“I needed it.”
		23.2	Desert	“I worked for it.”, “I deserved it, the survey was hard work.”
		23.3	Selfishness/greed	“I was selfish.”, “I acted on my own self-interest.”, “I wanted to keep more
**24**	Personal values	24		“I am a person who tries to use every situation to gain the maximum possible.”
**25**	No reason	25.1	lack reason	“There was no reason given...”, “No reason not to keep it all.”
		25.2	lack incentive	“There was no incentive given...”,
		25.3	lack recipient	“No one to share with...”, “
**26**	Guilt	26		“I do feel a small amount of guilt for keeping it.”
**27**	Frustration	27		“Felt a bit frustrated and decided to keep all....”
**28**	Surprise	28		“I tried to give it back.”, “I didn’t, I gave back 0.5.”

2.2.3.2 *Examples for category 23: No moral problem.*

- Academic research is usually paid for by grants. While it is possible that some of the cost is being paid for by the researchers, if I knew that this was the case then I would give back the .15, it seems more likely that the money if coming from an uncarig outside source and I am currently running low on money.
- I need the money, I turk entirely for survival and to save so I can pay down interest and rent an apartment at the end of my stay at university. I have to keep as much money as possible.
- I'm poor and unemployed.
- The additional money is helpful to me.
- I could really use all the money I can get !
- I need money to help out at home
- I'm trying to save up money for my sister's birthday present
- Honestly, I have spent a lot of time on this survey and I feel like I deserve to keep the money. Also, there is no consequence for the action of breaking my promise.
- tediousness of the work justified my keeping it
- I worked for the 15 cents
- I've worked hard for those five cents.
- I DO NOT WANT TO PAY ANYTHING BACK
- I want the entire amount
- I want to maximize profit for myself if there are not consequences for anybody else.

2.2.3.3 *Examples for category 25: No reason.*

- I don't see why I would choose to pay any money back.
- I wasn't given a reason to pay it back. If there was a reason, I would have considered it.
- I am not sure why I would give some of it back
- There did not appear to be any benefit in returning five cents. No ethical issue with keeping it.
- I didn't see any incentive to give the money back.
- I cannot remember any explanation as to why I might want to give it back...for instance, is it going to some worthy cause, or a partner? You didn't say. So I will keep it.

2.2.3.4 ***Examples for category 28: Surprise.*** *This category C28 was not reported in the manuscript, as participants' intention diverged from their literal decision. These participants had entered a decimal dollar value instead of a cent amount (and 0.05 cents led them to a prompt asking them to justify a broken promise/the non-payment of money). As participants were treated as having paid above threshold, percentages for this condition in the manuscript are relative to the remainder of the comments.*

- I didn't break my promise, I wrote .5 for five cents payback!
- I did not keep the money. I elected to pay back the .05 and typed that amount in the blank space. I don't go back on my promises, even in a game. You need to check the previous page, if you can, and I am assuming you can.
- I didn't break my promise. I stated .05
- I thought I typed in .05 on the previous screen–perhaps, I misunderstood. I did not intend to break my promise.

- I didnt, i gave back .05
- I actually tried to give back the money but I accidentally typed in the money in dollars (.05). I am still willing to give up the 5 cents.

2.3 Coding results

Here, we show the original percentages of codes including subcategories for each of the three groups of comments. Each comment could be classified into more than one category and not all comments could be categorized. The percentages are based on 58 comments for participants that rejected the higher payment (with 64 codes), 328 comments for participants who paid back the money (with 381 codes), and 146 comments of participants who did not pay back the money (with 141 codes).

Table S9 shows proportions in the first category separately for each experimental condition (that was unknown to the coders) and also collapsed across conditions (total column). Table S10 and Table S11 show the respective proportions for the second and third groups of comments.

Table S9

Proportion of comments in categories across conditions for participants not accepting the higher payment

Main	Sub	Promise (write)	Promise (click)	Ask	Control	Total
**31**	**31.1**	26.1	17.6	36.4	42.9	27.6
	**31.2**	17.4	17.6	18.2	28.6	15.5
	**31.3**	4.3	11.8	27.3	0.0	8.6
**32**	**32.1**	17.4	11.8	0.0	0.0	10.3
	**32.2**	21.7	23.5	9.1	42.9	25.9
	**32.3**	4.3	5.9	0.0	14.3	6.9
**33**	**33.1**	4.3	11.8	0.0	0.0	5.2
	**33.2**	0.0	17.6	0.0	0.0	5.2
**34**	**34**	0.0	0.0	9.1	0.0	1.7
**35**	**35**	8.7	0.0	0.0	0.0	3.4

For the manuscript, we selected and combined categories in the following way. In the first group, we selected categories 31 and 33, selected 32.1 and 32.2 into a third category and summarized all other codes as "other". In the second group, we selected categories 21, 23, and 25, and summarized all other codes as "other". In the third group, we selected categories 21, 23, and 25 and summarized others (except for the "surprise" category, as discussed above), as "other".

Table S10

Proportion of comments in categories across conditions for participants paying back the money

Main	Sub	Promise (write)	Promise (click)	Ask	Control	Total
**11**	**11.1**	45.3	36.9	7.1	0.0	28.0
	**11.2**	3.8	12.6	44.7	8.8	17.7
	**11.3**	16.0	18.4	9.4	2.9	13.7
	**11.4**	0.9	1.9	5.9	0.0	2.4
	**11.5**	0.9	1.0	2.4	0.0	1.2
	**11.6**	0.0	1.0	1.2	0.0	0.6
	**11.7**	2.8	1.9	14.1	5.9	5.8
**12**	**12.1**	2.8	1.9	1.2	0.0	1.8
	**12.2**	9.4	8.7	3.5	8.8	7.6
	**12.3**	1.9	2.9	7.1	14.7	4.9
**13**	**13.1**	1.9	1.0	1.2	0.0	1.2
	**13.2**	0.0	0.0	3.5	2.9	1.2
	**13.3**	5.7	5.8	2.4	0.0	4.3
	**13.4**	7.5	5.8	2.4	0.0	4.9
	**13.5**	1.9	0.0	0.0	0.0	0.6
	**13.6**	1.9	0.0	0.0	0.0	0.6
	**13.7**	0.9	0.0	0.0	2.9	0.6
**14**	**14.1**	0.9	1.0	1.2	0.0	0.9
	**14.2**	0.9	1.0	0.0	0.0	0.6
	**14.3**	0.0	0.0	0.0	0.0	0.0
**15**	**15**	0.9	0.0	0.0	14.7	1.8
**16**	**16.1**	3.8	3.9	5.9	26.5	6.7
	**16.2**	3.8	4.9	1.2	5.9	3.7
**17**	**17.1**	1.9	0.0	4.7	2.9	2.1
	**17.2**	0.0	1.9	0.0	0.0	0.6
	**17.3**	0.0	0.0	1.2	2.9	0.6
	**17.4**	1.9	1.9	0.0	0.0	1.2
**18**	**18**	0.0	1.0	0.0	2.9	0.6

Table S11

Proportion of comments in categories across conditions for participants not paying back the money

Main	Sub	Promise (write)	Promise (click)	Ask	Control	Total
**21**	**21.1**	10.0	6.7	2.4	1.3	2.7
	**21.2**	20.0	6.7	19.5	1.3	8.2
	**21.3**	0.0	0.0	0.0	0.0	0.0
	**21.4**	0.0	0.0	4.9	5.0	4.1
	**21.5**	0.0	6.7	9.8	0.0	3.4
**22**	**22**	0.0	0.0	2.4	5.0	3.4
**23**	**23.1**	10.0	6.7	9.8	10.0	9.6
	**23.2**	0.0	6.7	2.4	11.3	7.5
	**23.3**	10.0	20.0	17.1	21.3	19.2
**24**	**24**	0.0	0.0	2.4	1.3	1.4
**25**	**25.1**	0.0	0.0	7.3	20.0	13.0
	**25.2**	0.0	0.0	0.0	7.5	4.1
	**25.3**	0.0	0.0	0.0	6.3	3.4
**26**	**26**	0.0	0.0	0.0	1.3	0.7
**27**	**27**	0.0	0.0	2.4	1.3	1.4
28	***28***	40.0	*40.0*	*19.5*	*3.8*	*14.4*

3 Selected comments after Study 3

[Comments are presented as received.]

- I said it in the survey but the choice of breaking a promise and not following instructions from the requester or keep my promise but not follow instructions from the requester? I'll choose to listen to the requester.
- Well, I just want to say that I did guess the same number as the computer!!!!
- Yes, I was shocked but I did actually get the guessing game right. However, I did say I would pay the 10 back so I lied and did not put 0 but instead gave them the 10.
- Won the guess, didn't feel fair to take their bonus though.
- Very interesting study. I actually did guess 9 and that's what it generated!
- The questions were different from ones I've seen before. Thanks for the survey though! Sorry for keeping the twenty cents. I need it.
- That's a little odd phrasing since in the beginning it said that I promised I would pay 10 cents to the other participant but then in the instructions I had to enter 0 if I guessed the number correctly. So my options was to break a promise and not follow instructions or follow instructions and keep a promise? So what do you learn from the guessing game and the return of ten cents if you don't know how the game turned out? If someone lost and entered zero or someone won and gave back money anyway, you wouldn't know.
- I wish I could bring myself to lie about winning but it's only 10 cents that I'm losing so I suppose I will get over it.
- I was originally going to give back 10 cents to the other person, but at the end it said if I guessed the number correctly to put 0. It didn't feel right, and I almost decided to lie, but I didn't, because I don't like to lie. Even though no one would have known what I picked, I would have known. I still don't feel right that I wasn't allowed to give anything to the other player.
- i was confused on what to do. 0 or 10 cents. ahhhhh i'm trying to be good.
- I was confused about the instructions on the donation decision page. I had planned to donate 10 cents to the person, however I had won the guessing game. I wasn't sure which instructions took priority so I just put a 0.
- I swear I won the guessing game. 8 is my lucky number!
- I am now second guessing myself on whether I did or did not make a promise. For some reason, .10 is an amount that I seem to remember from the beginning of the study. I almost gave back .10 to the requester, but changed my mind at the last minute.
- I always try to keep my promises!

- Got damn, I don't remember if I made a promise to give back the ten cents or only had the option too. But strangely enough I did get the guessing game right.

4 Supplementary analyses and figures

4.1 Distribution of payback amounts across Studies 1a, 2a and 3

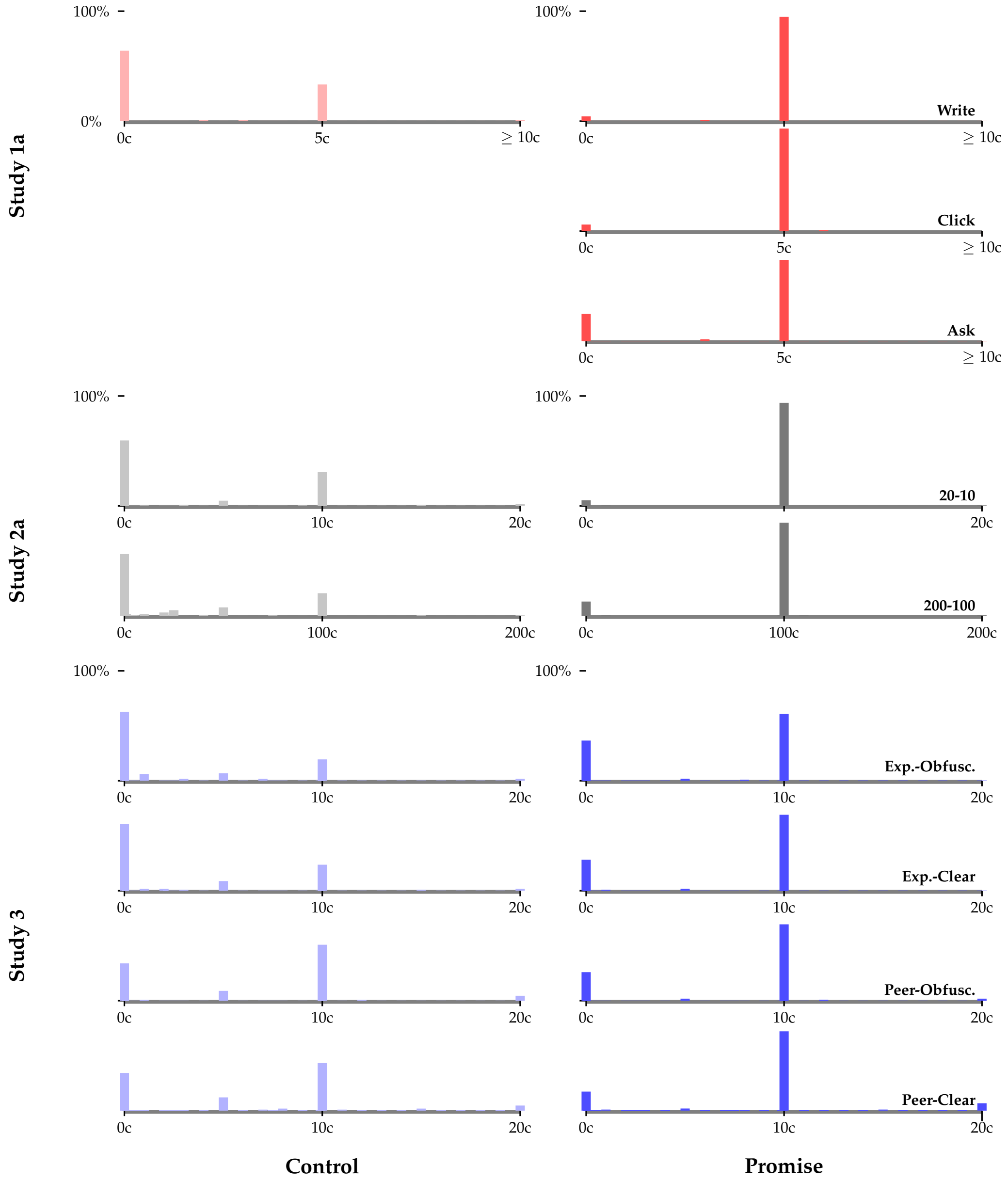

Figure S34. Distribution of payback amounts across Studies 1a, 2a and 3: Each histogram shows the distribution of individual payback amounts in each of the 16 conditions across studies. Bars represent relative percentages per condition among those participants who chose the higher amount. Condition labels on the right side for Study 2a and Study 3 refer to the entire rows, Study 1a has a single control condition.

4.2 Responses to the post-questionnaire in Study 2a

There were five different versions of the post-questionnaire in Study 2a. Participants saw one of these version, depending on the experimental conditions and actions taken:

1. Participants who did not accept the higher payment, answered questions regarding the reasons for non-acceptance. This was independent of experimental condition and results are shown in Figure S35.

2. Participants in one of the two promise conditions who did pay back the promised money amount, answered questions regarding reasons for keeping the promise. Results are presented in Figure S36.

3. Participants in one of the two promise conditions who did not pay back the promised money amount, answered questions regarding reasons for breaking the promise. Results are presented in Figure S37.

4. Participants in one of the two control conditions who did not pay back the suggested amount of money, answered questions regarding reasons for not paying back money. Results are presented in Figure S38.

5. Participants in one of the two control conditions who did pay back the suggested amount of money, answered questions regarding reasons for paying back money. Results are presented in Figure S39.

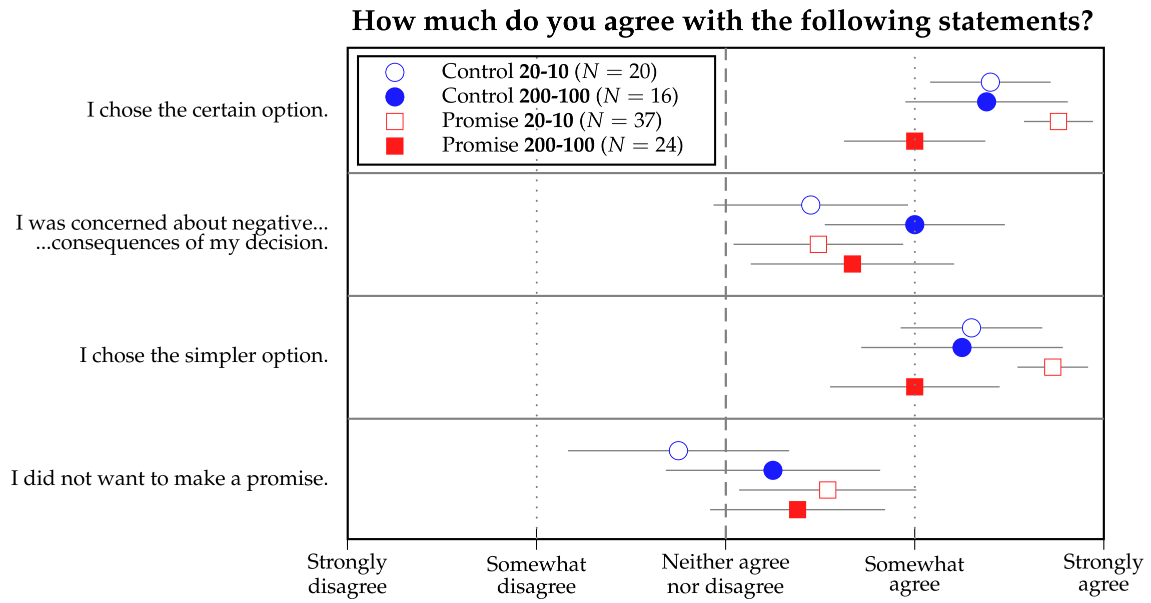

Figure S35. Means and 95% confidence intervals for responses to postquestionnaire questions from participants who rejected the higher payment split by experimental condition

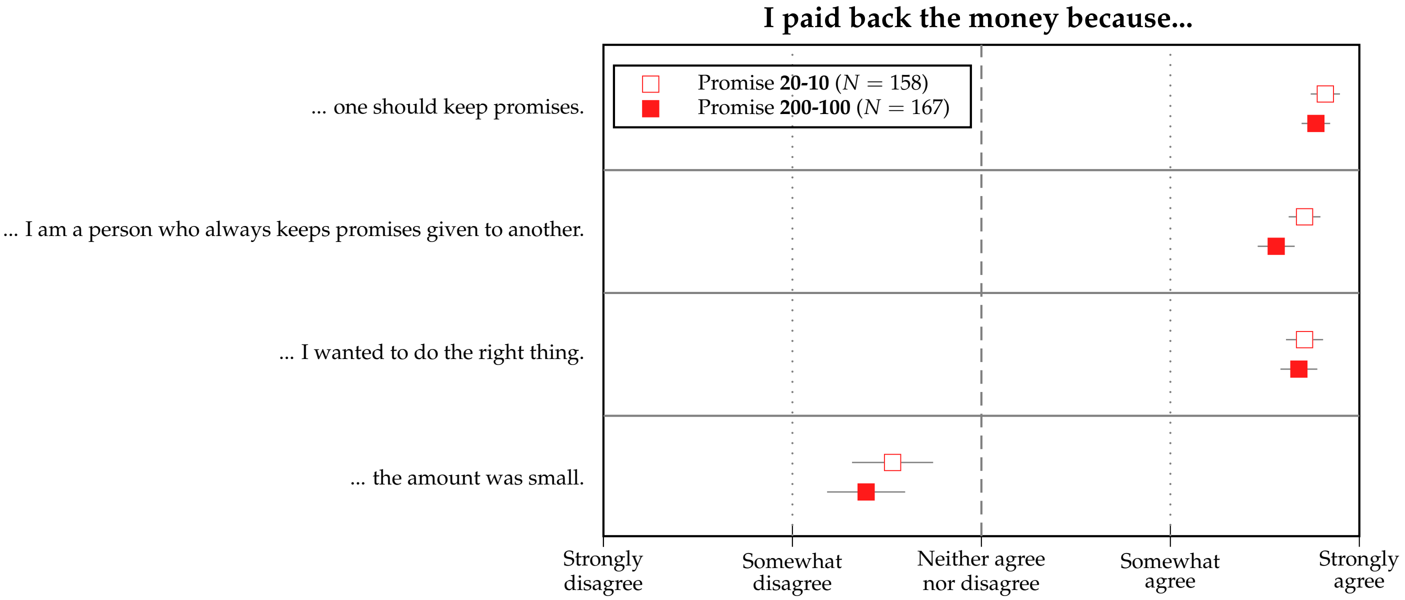

Figure S36. Means and 95% confidence intervals for responses to postquestionnaire questions from participants who kept their promise split by experimental condition

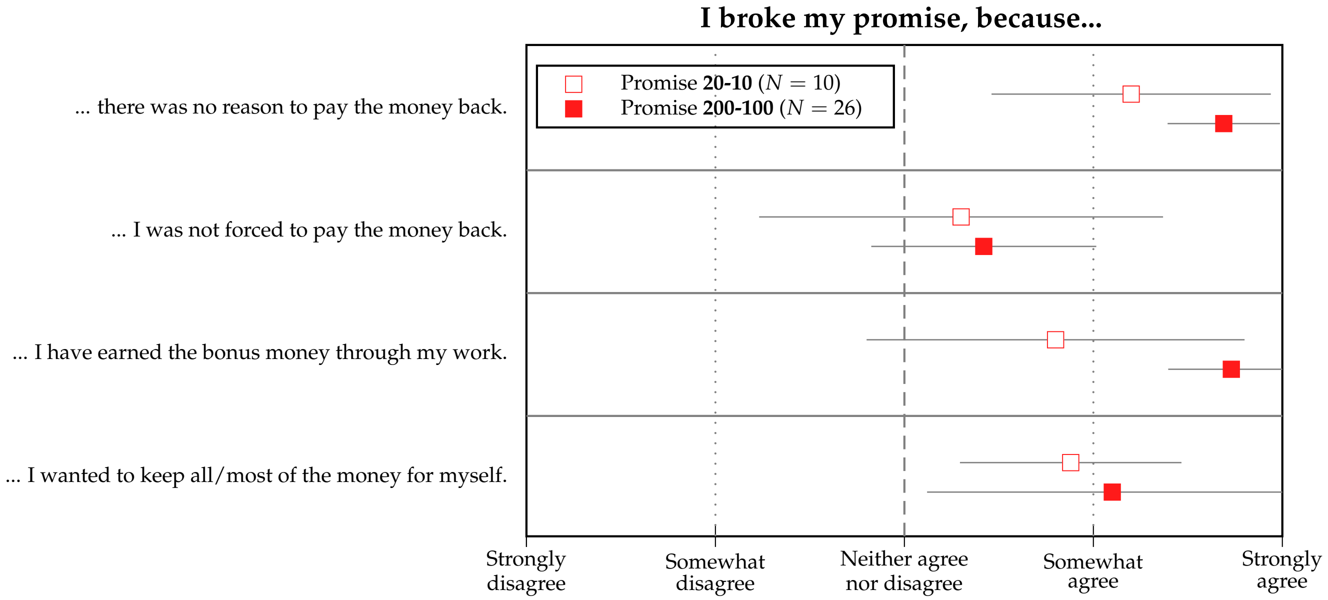

Figure S37. Means and 95% confidence intervals for responses to postquestionnaire questions from participants who broke their promise split by experimental condition

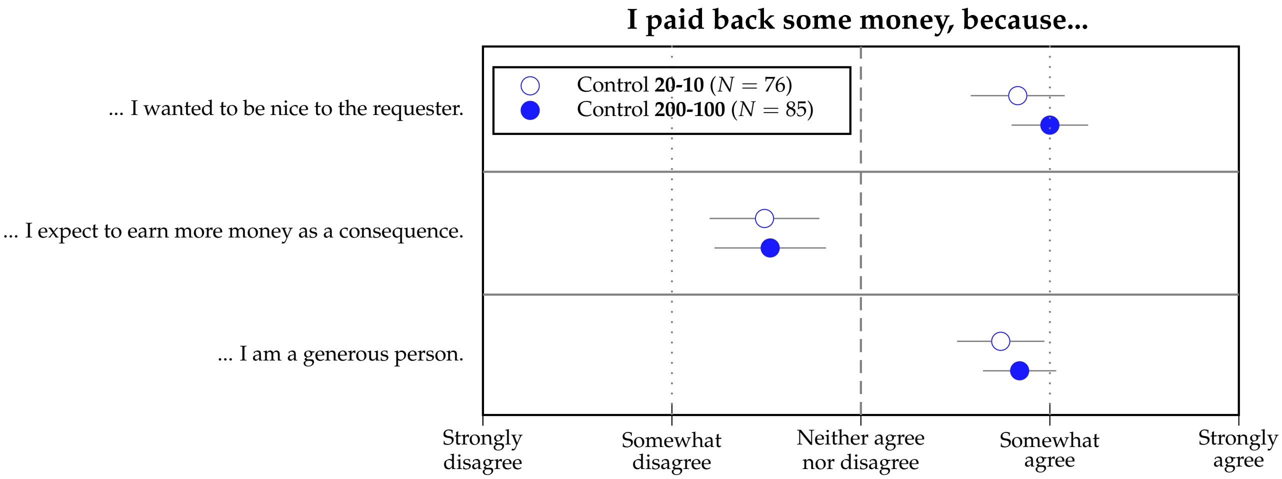

Figure S38. Means and 95% confidence intervals for responses to postquestionnaire questions from participants who paid back money for the two control conditions

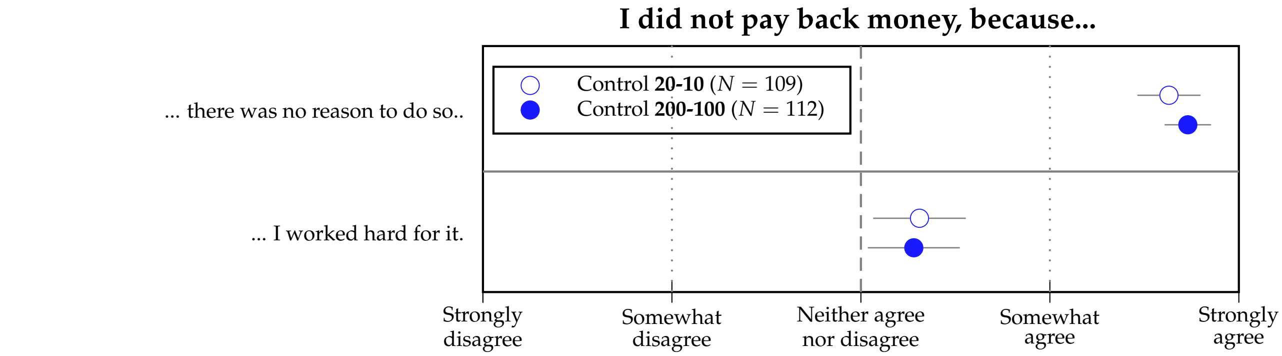

Figure S39. Means and 95% confidence intervals for responses to postquestionnaire questions from participants who did not pay back the suggested amount for the two control conditions

4.3 PANAS results

Figure S40 shows PANAS sub-scale means and CIs for Study 1a, Figure S41 the respective statistics for Study 2a.

In the paper, we report the results for the lenient filter. Applying the strict filter (and reducing the power of the analysis), some of the reported effects are no longer significant. For positive affect, we still observe the significant effect for the binary payback factor ($F(1, 443) = 7.73, p = .006$). This is also true for negative affect ($F(1, 443) = 14.58, p < .001$), but there is no longer a significant effect of promise condition ($F(1, 443) = 3.75, p = .054$) and no significant interaction effect for these variables ($F(1, 443) = 2.04, p = .47$). Figure S41 shows results with the strict filter applied.

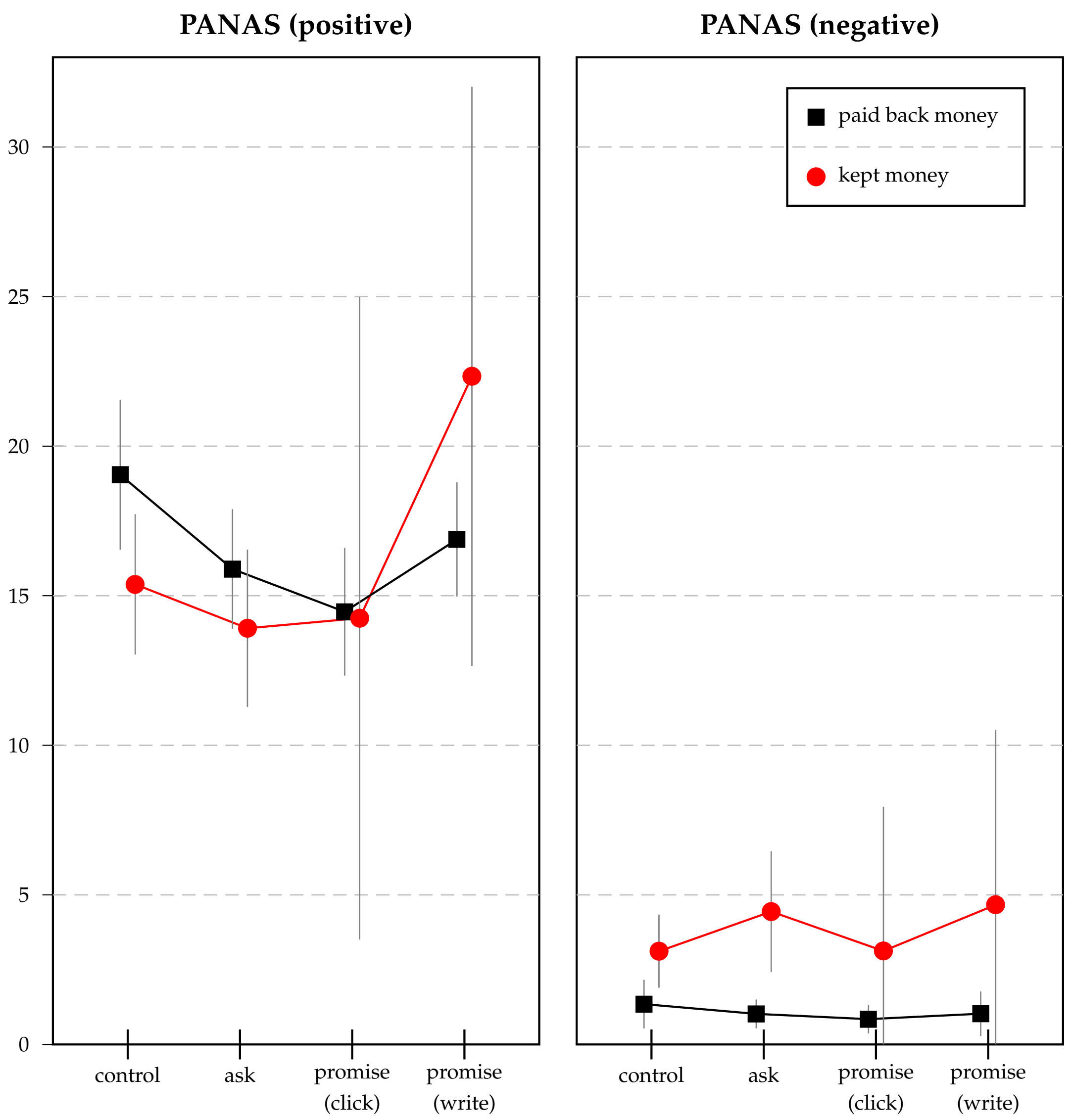

Figure S40. Means and 95% confidence intervals for the PANAS subscales across conditions in Study 1a: Means are presented separately for participants who paid back at least 5 cents (black squares) and participants who kept at least some money (red circles)

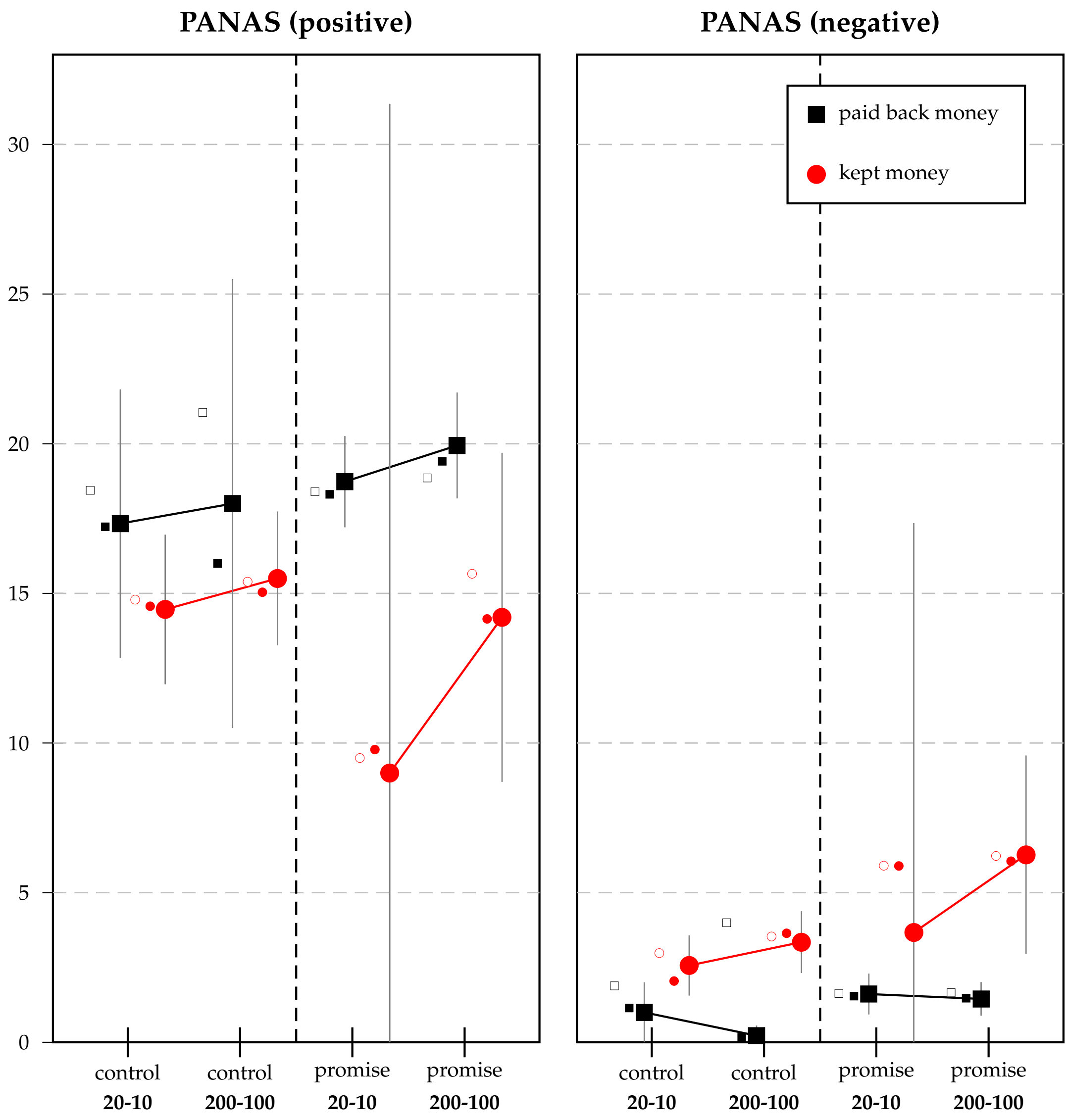

Figure S41. Means and 95% confidence intervals for the PANAS subscales across conditions in Study 2a: Means are presented separately for participants who paid back at least 5 cents (black squares) and participants who kept at least some money (red circles). CIs and means are shown for the strict filter condition, means for the lenient filter are given as smaller filled symbols, means for the unfiltered data as smaller unfilled symbols.
